# Development and application of high resolution SPEI drought dataset for Central Asia

**DOI:** 10.1038/s41597-022-01279-5

**Published:** 2022-04-14

**Authors:** Karim Pyarali, Jian Peng, Markus Disse, Ye Tuo

**Affiliations:** 1 Chair of Hydrology and River Basin Management, Technical University of Munich, Arcisstrasse 21, 80333 Munich, Germany; 2 Department of Remote Sensing, Helmholtz Centre for Environmental Research−UFZ, Permoserstrasse 15, 04318 Leipzig, Germany; 3 Remote Sensing Centre for Earth System Research, Leipzig University, 04103 Leipzig, Germany

**Keywords:** Hydrology, Natural hazards

## Abstract

Central Asia is a data scarce region, which makes it difficult to monitor and minimize the impacts of a drought. To address this challenge, in this study, a high-resolution (5 km) Standardized Precipitation Evaporation Index (SPEI-HR) drought dataset was developed for Central Asia with different time scales from 1981–2018, using Climate Hazards group InfraRed Precipitation with Station’s (CHIRPS) precipitation and Global Land Evaporation Amsterdam Model’s (GLEAM) potential evaporation (E_p_) datasets. As indicated by the results, in general, over time and space, the SPEI-HR correlated well with SPEI values estimated from coarse-resolution Climate Research Unit (CRU) gridded time series dataset. The 6-month timescale SPEI-HR dataset displayed a good correlation of 0.66 with GLEAM root zone soil moisture (RSM) and a positive correlation of 0.26 with normalized difference vegetation index (NDVI) from Global Inventory Monitoring and Modelling System (GIMMS). After observing a clear agreement between SPEI-HR and drought indicators for the 2001 and 2008 drought events, an emerging hotspot analysis was conducted to identify drought prone districts and sub-basins.

## Abstract (summary)

**Table:** 

Measurement(s)	Drought index
Technology Type(s)	Remote sensing products
Factor Type(s)	Standardized Precipitation Evapotranspiration Index
Sample Characteristic - Environment	drought
Sample Characteristic - Location	Central Asia

## Background & Summary

**Fig. 1:**
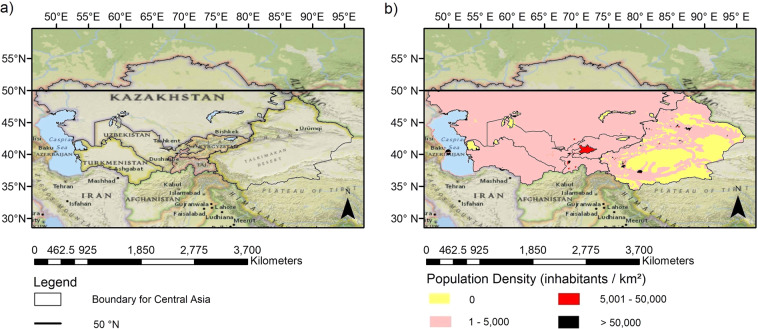
Part (**a**) outlines the study area and part (**b**) presents its 2015 population distribution in the figure. Both parts (**a**) and (**b**) are bounded by 50°N latitude, due to unavailability of high resolution precipitation data above 50°N. The maps also show the countries and topographical features which lie within the region of interest.

The study area, as shown in Fig. 1, comprises of six different countries which are Tajikistan, Kazakhstan, Turkmenistan, Uzbekistan, parts of China, and Kyrgyzstan. The precipitation data used in this study are only available for regions below 50°N therefore the study area had to be clipped accordingly. Central Asia covers approximately 5.65 million km^2^ of land^1^ and the topography of the region is very diverse from mountainous terrain to low lying basins and from deserts to grasslands^2^. The study area is very far away from an ocean and the mountain ranges in the south-east Asia further blocks the amount of moisture reaching Central Asia. Therefore, mostly arid conditions prevail in the regions with a typical temperate continental climate^3^. The main source of water for the region are the glaciers of Tianshan Mountains^4^. The population density of the region is low compared to neighbouring Southeast Asia. Figure 1 shows very few locations which have a large population of more than 50,000 people per square km^5^. According to Zhi Li *et al*.^6^, the temperature of Central Asia has increased sharply since 1997 and as expected the decade of 2007 to 2017 was the warmest period for region’s recorded history. The poor management of water resources and effects of increasing temperature and varying precipitation patterns due to climate change have led to an increase in severity of water resource deficit. The effects of increase in temperature, exacerbated by human activities, were very clear when the Aral Sea shrank over a very short time period^2^.

### Background/Introduction

A drought is an environmental disaster characterised by a prolonged dry period and is caused by a lack of precipitation, which can take place anywhere on land^7,8^. The definition of a drought varies in the academic literature. However, there is a consensus that an anomaly in temperature or precipitation that persists for a long period of time across a region reduces the volume of soil moisture, groundwater, and surface runoff^9^. The impacts of droughts are non-structural and difficult to quantify due to its slow creeping nature, but a lack of water could lead to crop failure, multiple consecutive crop failures could start a famine and result in human migrations^7^. In last half century multiple droughts have occurred around the world, for example, the drought of 1988 in United States costed its economy $40 billion worth in damages^10^, the drought of 2005 across Spain and Portugal decreased the cereal yields of European Union (EU) by ten percent^11^, a multi-year drought in Central and Southwest Asia affected 60 million people during 1999 – 2000^12^, in Australia the drought of 2006 caused an estimated $3.5 Billion worth of damages to the local economy^13^ and in Africa severe droughts in 1980s, 1970s and 1960s were followed by famines in the region^8^. Therefore, understanding the socio-economic and ecological aspects of drought is as important as the hydrologic or meteorological aspects of the event. It is key to note that a drought itself is not a disaster, but the lack of resilience of a community to cope with its impacts makes it one^7^.

Generally, droughts are categorized into four types: meteorological (indicator lack of precipitation), agricultural (indicator low soil moisture), hydrological (indicator low runoff or ground water table) and socio-economic drought (social indicators income and access to water)^8,14,15^. Furthermore, each drought can be characterized according to its severity, duration, and intensity. The quantification of the different types of droughts depends on the time scale at which the water deficit is accumulated. The different time scales respond to different source of accessible water. Therefore, to estimate the different types of droughts multiple drought indices have been developed. One of the most widely accepted drought indices is Standardized Precipitation Index (SPI) which has a simple application and can characterize different types of droughts by varying the time scales. SPI is recommended by World Meteorological Organization (WMO) and it only needs precipitation data for estimation^8,14,16^. Vicente *et al*.^17^, argued that SPI ignores the influence of temperature on water deficit, which leads to misrepresentation of actual drought conditions specifically in arid regions. Additionally, rising average global temperature due to climate change will amplify the role of temperature on drought propagation. Thus, Standardized Precipitation Evaporation index (SPEI) was developed, which is similar to SPI, except it uses water deficit values instead of precipitation for better representation of drought conditions^17^.

There are two widely used global SPEI datasets, which are SPEIbase^18^ and Global Precipitation Climatology Centre Drought Index (GPCC-DI)^19^. Their spatial resolutions are 0.5° (≈ 50 km) and 1° (≈ 110 km), respectively. SPEI-base was developed using CRU Time Series and GPCC-DI was developed using GPCC precipitation data and Climate Prediction Center’s temperature data. A lack of finer resolution data meant that most studies could only be conducted at regional levels^2,3,20^ because the resolution of the input dataset is too coarse to apply drought indices at district or sub basin level^21^. However, recently developed high-resolution precipitation data^22^ and evapotranspiration data^23^ made it possible to estimate SPEI at a spatial resolution of 5 km. As benefits, some researchers prepared high-resolution SPEI data for the whole continent of Africa^15^, which presented reliable performances in characterizing drought events. Similar studies are missing but could be valuable in the data scarce continental region of Central Asia. What performance these high-resolution data will have for drought research of another domain with different climate and topographic characteristics is an open question.

In this study, a high-resolution (5 km) SPEI drought index was prepared for the entirety of Central Asia. The temperature of the region is currently the warmest it has ever been in the recorded history^24^, it is prone to droughts^25^ and studies have found a significant drying pattern between 2003–2015^2^. Furthermore, the available observed meteorological data of the region is not continuous^1^ and very scarce. As a result, more research is required to understand how sensitive the region is to climate change. The high-resolution SPEI dataset produced in this study will help water managers, policy makers and local stake holders to improve their risk analysis and plan their response accordingly. Lastly, to enhance the available knowledge for this region an emerging hotspot analysis is conducted for SPEI values of 6-month and 48-month time scale, where a time scale indicates the period of water deficit accumulation.

### Summary

The region of Central Asia is data scarce therefore, a high-resolution SPEI drought dataset was developed in this study for the region. The input data used were CHIRPS’s precipitation dataset and GLEAM’s potential evaporation dataset, both remote sensing products. SPEI dataset for forty-eight different time scales were produced and the time period ranged from 1981–2018, a total of 38 years. The results were validated using CRU’s SPEI data, GLEAM’s root zone soil moisture and GIMMS’s NDVI data. Overall, the high-resolution SPEI dataset displayed high spatial and temporal correlation with CRU’s SPEI vales, high to moderate correlation with GLEAM’s root zone soil moisture, and satisfactory to low correlation with GIMMS’s NDVI. The evaluation of the dataset indicated that SPEI-03 (three month time scale), SPEI-06 and SPEI-09 performed best in capturing RSM values, while SPEI-06, SPEI-09 and SPEI-12 gave the highest NDVI values. The overall performance of the dataset is considered good; therefore, an emerging hotspot analysis was conducted on the dataset to observe drought conditions on a district or basin scale. The emerging hotspot analysis identified regions with oscillating hot and cold spot patterns for the time scales of 1, 3, 6, 9, 12, 24, 36, and 48-month, but it failed to provide any other conclusive pattern due to the periodic nature of the drought indices. Lastly, the high-resolution (5 km) of the SPEI-HR dataset produced in this study, to our knowledge, is the best available resolution for a drought index in Central Asia.

## Methods

### High-Resolution SPEI calculation

In this study, following the work of Peng *et al*.^15^, a high-resolution drought index dataset containing SPEI values for Central Asia was prepared for 48 different time scales using the method proposed by Vicente-Serrano *et al*.^17^. The input data used was CHIRPS precipitation dataset, which has a monthly temporal resolution and a 5 km spatial resolution, and the GLEAM evaporation data, which was downscaled from 25 km resolution to 5 km, using bilinear interpolation, and has a monthly temporal resolution.

To compute SPEI we require water deficit values (D), which are calculated by subtracting E_p_ from precipitation (P) values using the following equation:

1 $${D}_{i}={P}_{i}-E{p}_{i}$$

Please note that different E_p_ methods could result in different SPEI estimations^26–28^. To improve the regional drought assessment, such impacts should be addressed by further studies when regional data will be available for verification. It requires a standalone research and beyond the scope this Data Descriptor paper.

The water deficit values are aggregated depending on the time scale before being standardized using the log-logistic distribution with the following probability density function:

2 $$f\left(x\right)=\frac{\beta }{\alpha }\left(\frac{x-\gamma }{\alpha }\right){\left[1+\left(\frac{x-\gamma }{\alpha }\right)\right]}^{-2}$$

where the parameters are β, γ and α representing shape, origin and scale. The probability distribution function for log logistic distribution, is given by,

3 $$F\left(x\right)={\left[1+{\left(\frac{\alpha }{x-\gamma }\right)}^{\beta }\right]}^{-1}$$

Then the SPEI values can be estimated by standardizing the $F\left(x\right)$ values using the following equation

4 $$SPEI=W-\frac{{C}_{0}+{C}_{1}W+{C}_{2}{W}^{2}}{1+{d}_{1}W+{d}_{2}{W}^{2}+{D}_{3}{W}^{3}},$$

where C_0_ = 2.515517, C_1_ = 0.802853, C_3_ = 0.010328, d_1_ = 1.432788, d_2_ = 0.189269, d_3_ = 0.001308. The value of *W* depends on the probability of exceedance *P* as shown below

5 $$W=\sqrt{-2ln\left(P\right)},$$

where $P=1-F(x)$ when $P\le 0.5$, but in case $P > 0.5$, then the *P* is replaced by $1-P$ and the sign of SPEI is reversed^17^.

The drought index values within the datasets vary from extremely wet to extremely dry and two SPEI ranges, or thresholds were found in the literature, as shown in Table 1.

**Table 1:** Classification of SPEI values based on two different thresholds found in the literature.

SPEI^15,53,54^	SPEI^52^	Category
2 and above	1.83 and above	Extremely wet
1.5 to 1.99	1.43 to 1.82	Very wet
1.0 to 1.49	1.0 to 1.42	Moderately wet
−0.99 to 0.99	−0.99 to 0.99	Near Normal
−1.0 to −1.49	−1.0 to −1.42	Moderately dry
−1.5 to −1.99	−1.43 to −1.82	Severely dry
−2 and less	−1.83 and less	Extremely dry

Due to low hydroclimatic variability the SPEI results are not reliable for sparsely vegetated and barren areas, therefore during evaluation the SPEI values were masked for these two specific land covers^18,29^ using Moderate Resolution Imaging Spectroradiometer (MODIS) land cover type product (MCD12Q1)^30^.

### Evaluation Criteria

The results were evaluated by comparing high-resolution SPEI results with CRU SPEI results for some of the time scales (i.e. 1, 3, 6, 9, 12, 24, 36, & 48 months). The high-resolution results were upscaled to 50 km to ensure we have consistent data for comparison. The correlation between high-resolution SPEI and CRU SPEI were evaluated both temporally and spatially. Furthermore, to observe the performance of high-resolution SPEI dataset, the SPEI-06 was compared with NDVI and root zone soil moisture (RSM). Then the spatial mean or area mean of both high and coarse resolution SPEI-06 were compared with the area mean of NDVI and RSM over the eight different time scales aforementioned. The reason behind using SPEI-06 was the findings of Törnros and Menzel, (2014)^31^, who observed that the 6-month SPEI (or SPEI-06) has the highest correlation with soil moisture and best captures the variations of NDVI.

The NDVI is a measure of health of the vegetation and the RSM is an indicator for agricultural droughts. The NDVI values were obtained from GIMMS dataset (1981–2015) and RSM data was collected from GLEAM (1981–2018). The high-resolution of SPEI results had to be resampled according to the product (NDVI or RSM) it was compared to. The NDVI and RSM products were standardized before being compared to the resampled SPEI. For standardization, the time series of each pixel were ordered according to months and then the time series of each month were standardized using its mean and standard deviation as shown in the following equation, suggested by Meng Zhao *et al*.^29^, where i is month and j is the year.

6 $$standardized\;{X}_{\left(i,j\right)}=\frac{{X}_{\left(i,j\right)}-mean\left(X\right)}{standard\;deviation\left(X\right)}$$

In the following sections, high-resolution SPEI will be referred as SPEI-HR and the coarse resolution SPEI results from Climate Research Unit data are termed as SPEI-CRU. The Pearson’s correlation was used to analyse the correlation between the different variables and only statistically significant results were accepted. The rest were converted into “Not Available”. The following Table 2 shows all the correlations carried out.

**Table 2:** SPEI-HR correlation analysed with different products to evaluate its performance.

Variable Combination	Spatial Correlation	Temporal Correlation
SPEI-HR vs SPEI-CRU	**✓**	**✓**
SPEI-HR vs NDVI	✓**	✓*
SPEI-HR vs RSM	✓**	✓*

Note: * indicates that the spatial correlation was performed only for 6-month time scale.

** indicates the use of area mean or spatial mean to analyze only the temporal correlation

### High-Resolution Emerging Hot Spot Analysis

As a potential application, an emerging hot spot analysis was conducted on the high-resolution SPEI drought dataset prepared in this study. The analysis was performed using the Getis-Ord-Gi* statistic that is available in “Spatial Statistics Toolbox” of ArcGIS Pro software. The analysis identifies a range of statistically significant patterns depending on the value of its Gi* statistic^32^. A Gi* statistic proportionally compares the sum of a local feature and its adjacent feature to the sum of all the features in that study, using:

7 $${G}_{\left(i\right)}^{* }=z-score=\frac{{\sum }_{j=1}^{n}{w}_{\left(i,j\right)}{x}_{j}-mean\left(X\right){\sum }_{j=1}^{n}{w}_{\left(i,j\right)}}{S\sqrt{\frac{n{\sum }_{j=1}^{n}{w}_{\left(i,j\right)}^{2}-{\left({\sum }_{j=1}^{n}{w}_{\left(i,j\right)}\right)}^{2}}{n-1}}},$$

where ${x}_{j}$ is the attribute value of the feature j, ${w}_{\left(i,j\right)}$ is the spatial weight between feature i and j, and n is the total number of features.

8 $$mean\left(X\right)=\frac{{\sum }_{j=1}^{n}\;{x}_{j}}{n},$$

9 $$S=\sqrt{\frac{{\sum }_{j=1}^{n}\;{x}_{j}^{2}}{n}-{\left(mean\left(X\right)\right)}^{2}}.$$

The Gi* statistic is evaluated for each feature in the study and a z-score and p-value is obtained. The z-score is only statistically significant when the difference between estimated local sum and expected local sum is too large and cannot be attributed to randomness. A hotspot has a high z-score and small p-value which indicates a significant cluster of high values around the local feature. While a cold spot has a low negative z-score and small p-value which represents a significant cluster of low values. In other words, hotspots occur where the drought index is high in the pixel and in the neighbourhood of that pixel. In this study a hotspot indicates accumulation of high SPEI values (or wet conditions) in time and space around a pixel, while a cold spot represents low SPEI values (or dry conditions) around a pixel in time and space^33,34^.

## Data Records

The high resolution (5 km) SPEI drought dataset produced in this study for Central Asia is archived at the Centre for Environmental Data Analysis (CEDA)^35^. The dataset is publicly available and can be accessed as follows: Pyarali, K.; Peng, J.; Disse, M.; Tuo, Y. High resolution Standardized Precipitation Evapotranspiration Index (SPEI) dataset for Central Asia. NERC EDS Centre for Environmental Data Analysis (2022).

### CHIRPS

CHIRPS is a state-of-the-science high-resolution precipitation dataset. It has a quasi-global coverage, where the available data spans over the entire longitude, but the latitude ranges between 50°S to 50°N. The development of CHIRPS dataset can be divided into three main components 1) Climate Hazards group Precipitation climatology (CHP_clim_), 2) Satellite only Climate Hazards group Infrared Precipitation (CHIRP), and 3) blending station data to produce CHIRPS^22^.

CHIRPS is the final gridded precipitation dataset that was used in this study. The blending between CHIRP and observed station data is carried out by applying a modified inverse distance weighting interpolation, where the interpolation for any pixel depends on the weighted average of the ratios between the CHIRP value of the pixel and the five closest stations. The value of the pixel is further adjusted depending on the correlations between it and the nearest station and between the CHIRP value and the true precipitation value. The final CHIRPS value for the pixel is a combination of unadjusted and bias adjusted CHIRP data^22^

CHIRPS is specifically developed to observe conditions which indicate the emergence of agricultural drought and global environmental change on land. The dataset provides context for the recent extreme climate events relative to historical observations, with an unprecedented high spatial and temporal resolution in the domain of global terrestrial products. The validation and application studies of CHIRPS suggest that it performs well across Turkey with a monthly and decadal correlation of 0.81 and 0.78, respectively^36^, over southern river basins in China with a correlation between 0.44 to 0.46^37^, throughout the Indian Subcontinent with a correlation of 0.80^38^ and across Africa^15^, while poor performances with correlation between 0.21 to 0.34 were recorded over north-western and northern river basins in China^37^.

In this study CHIRPS precipitation data were used to prepare a drought index dataset with a 5 km resolution and a time period of thirty-eight years from 1981 to 2018. More details regarding the product can be found in the paper authored by Chris Funk *et al*.^22^.

### GLEAM

GLEAM is a model that estimates Global E_p_ and RSM using remote sensing products. The dataset this model provides has a spatial resolution of 0.25° (≈ 25 km), a monthly temporal resolution and spans from 1980 to 2018. The aim of developing GLEAM was to provide a consistent and long term observed dataset for hydrological variables, which are sparsely available for most regions of the world^23^. The model is made up of four modules: 1) Potential evaporation, 2) Rainfall interception, 3) Soil module and 4) Stress module.

GLEAM estimates E_p_ (mm day^−1^) using the Priestley and Taylor (1972)^39^ equation, as presented below. This method provides an E_p_ value, which is based on land cover, net radiation (R_n_), and air temperature of the region.

10 $$\lambda {E}_{p}=\alpha \frac{\Delta }{\Delta +\psi }\left({R}_{n}-G\right),$$

where λ is the latent heat of vaporization (MJ kg^−1^), Δ is the slope of saturated water vapor temperature (kPa K^−1^), ψ is the psychometric constant (kPa K^−1^), α is the unitless Priestly and Taylor coefficient ( = 1.26) and *G* is the ground heat flux (Wm^−2^)^23^.

Furthermore, the root zone soil moisture, RSM, is estimated by GLEAM using a multi-layer water balance approach, where the inputs are net precipitation (precipitation minus intercept loss) and snow melt, while the outputs are evaporation and drainage. The depth of the root zone is a function of the type of land cover, if the land cover is tall vegetation the models divides the depth into three layers (0 – 10, 10 – 100, and 100 – 250 cm), for low vegetation the depth is divided into two layers (0 – 10, 10 – 100 cm), bare soil only one layer used (0 – 10 cm) and if the land cover is forest than the interception model developed by Gash (1979)^40^ and improved by Valente *et al*.^41^ is used^23^.

Both E_p_ and RSM are highly validated products used in multiple studies^15^. RSM is validated using *in-situ* soil moisture measurements from international Soil Moisture Network (ISMN)^42^, the validation results give an average correlation ranging between 0.49 to 0.64 for multiple datasets. Unlike RSM, E_p_ is not validated directly, rather the actual terrestrial evaporation E_a_, which is based on E_p_, is validated. The *in-situ* measurements for validating terrestrial evaporation are collected from fluxnet.org and the results show that the GLEAM estimated evapotranspiration and the *in-situ* measurements are highly correlated and the correlation ranges between 0.78 to 0.81 for all the datasets^23^.

In this study, GLEAMS potential evaporation (or “terrestrial evaporation” or “evapotranspiration”) was used to estimate water deficit from 1981–2018, while root-zone soil moisture was used to evaluate the performance of the SPEI estimation.

### CRU

CRU provides gridded observations of the whole Earth (except Antarctica) on a monthly temporal resolution and a 0.5° (≈ 50 km) spatial resolution. The observations range from 1901 to 2018. The data consist of primary variables (i.e. mean temperature at 2 m, diurnal temperature range at 2 m and monthly precipitation), secondary variables (i.e. vapor pressure, wet days and cloud cover percentage) and derived variables (i.e. frost days, minimum temperature at 2 m, maximum temperature at 2 m and potential evapotranspiration). CRU data use angular distance weighting (ADW) to interpolate observed data over the gridded land surface. The application of CRU data is very diverse from the sphere of climate research to the global financial and insurance sector^43^.

The precipitation data from stations around the globe are collected and converted into anomalies using the mean of every individual station. These anomalies are then interpolated over the 0.5° × 0.5° grid using ADW method and then converted back into actual precipitation using climatologies. Potential evaporation is one of the derived variables in CRU data, it is estimated using the Penman-Monteith equation^44^, PM, which is a method approved by Food and Agriculture Organization (FAO). The method uses the gridded vapor pressure, mean temperature, cloud cover and static average wind field observations. The application of PM in context of CRU data is explained in paper authored by M. Ekström *et al*.^45^.

The precipitation values are validated using Deutscher Wetterdienst (DWD) Global Precipitation Climatology Center’s (GPCC) precipitation data. The correlation, R, between CRU and GPCC precipitation for a Global scale is 0.92, the correlation decreases in the Southern Hemisphere and increases in Northern Hemisphere. This could be due to the distribution of available observation data^43^. In this study, we worked with the precipitation (mm/mon) data and the potential evaporation (mm day^−1^) data to evaluate the performance of 0.05° SPEI estimated over Central Asia.

### GIMMS NDVI

GIMMS was used to prepare a 3^rd^ generation NDVI dataset, which covers the whole Earth, except Antarctica, and is available for a period of 1981 to 2015. The data have a spatial resolution of 8 km and a monthly temporal resolution. The GIMMS model estimate NDVI using Advanced Very High-Resolution Radiometer (AVHRR) sensors. Bayesian method was used to derive calibration parameters from the AVHRR NDVI values. The uncertainty evaluation gave an error of ± 0.005 for the entire NDVI dataset^46^.

The dataset is widely accepted and has been used for multiple purposes for example, to evaluate the degradation of land in Sahel-Sudanian zone of Africa^47^, monitor biomass production in an ecosystem^48^, assess vulnerability of agriculture in India to variation in rainfall as a consequence of climate change^49^ and validation of drought index in Africa^15^.

Since, NDVI values can be used as a proxy to observe the growth of vegetation, we used them in this study to validate the performance of our SPEI dataset by investigating the effects of drought on vegetation.

## Technical Validation

### Inter-comparison between SPEI-HR and SPEI-CRU

**Fig. 2:**
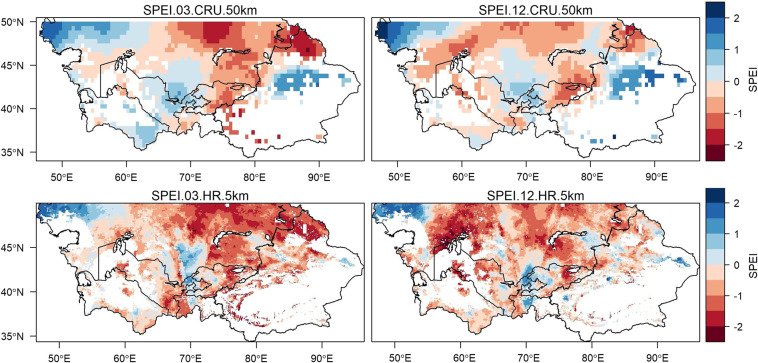
Spatial patterns covering Central Asia representing 3-month and 12-month SPEI values for high spatial resolution (SPEI-HR) and coarse spatial resolution (SPEI-CRU) from June 1990. GLEAM’s potential evaporation and CHIRPS’s precipitation data was used to develop high-resolution SPEI data (5 km), while CRU TS datasets were used for preparing coarse resolution SPEI data (50 km). The definition of SPEI range is presented in Table 2.

A direct comparison between high-resolution SPEI and coarse resolution SPEI for a couple of time scales, 3-month and 12-month, is presented in Fig. 2. The spatial patterns in Fig. 2 belong to June 1990. It can be seen that the emerging SPEI patterns between high and coarse resolution data, for both time scales, performed similar to each other. However, the level of detail in the high-resolution dataset provided a better understanding of the drought and its corresponding climate features at a finer scale. In SPEI 12 CRU a large portion of the pattern falled within the range of −0.99 to 0.99, which according to Table 2 can be defined as near normal conditions. However, the same portion, when viewed in SPEI 12 HR, showed that drought conditions over some pixels deviate from near normal range, indicating possibly moderate, severe, or extreme conditions. This highlighted the advantages of developing a high-resolution drought dataset. Furthermore, the different drought patterns between 3-month and 12-month SPEI were due to varying aggregation of water deficit depending on the time scale. The SPEI results from different time scales helped in differentiating between meteorological, agricultural, hydrological, groundwater, or any other type of drought. In Fig. 2 the northern and north-western region around Aral Sea (60°E,45°N) shows a near normal condition for the 3-month time scale, but moderate, severe, and extreme drought conditions for the 12-month time scale. The local stake holders in this region should prepare for a long-lasting drought and should not misjudge the conditions based on short term near normal patterns.

**Fig. 3:**
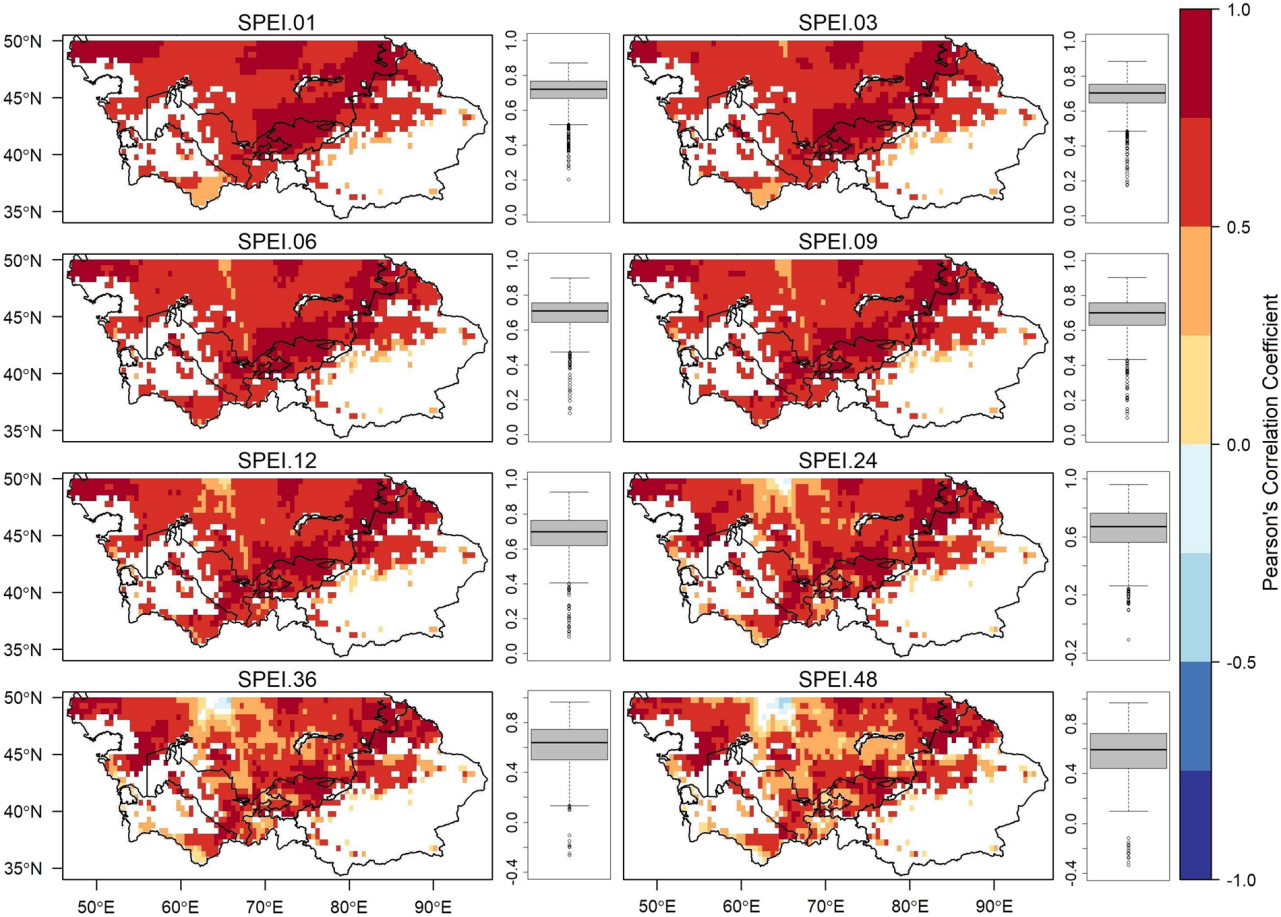
Statistically significant (p < 0.05) temporal correlation (R) between SPEI-HR and SPEI-CRU at different time scales. The box and whiskers plot represent the distribution of Pearson’s correlation coefficient values (R) for each time scale.

**Fig. 4:**
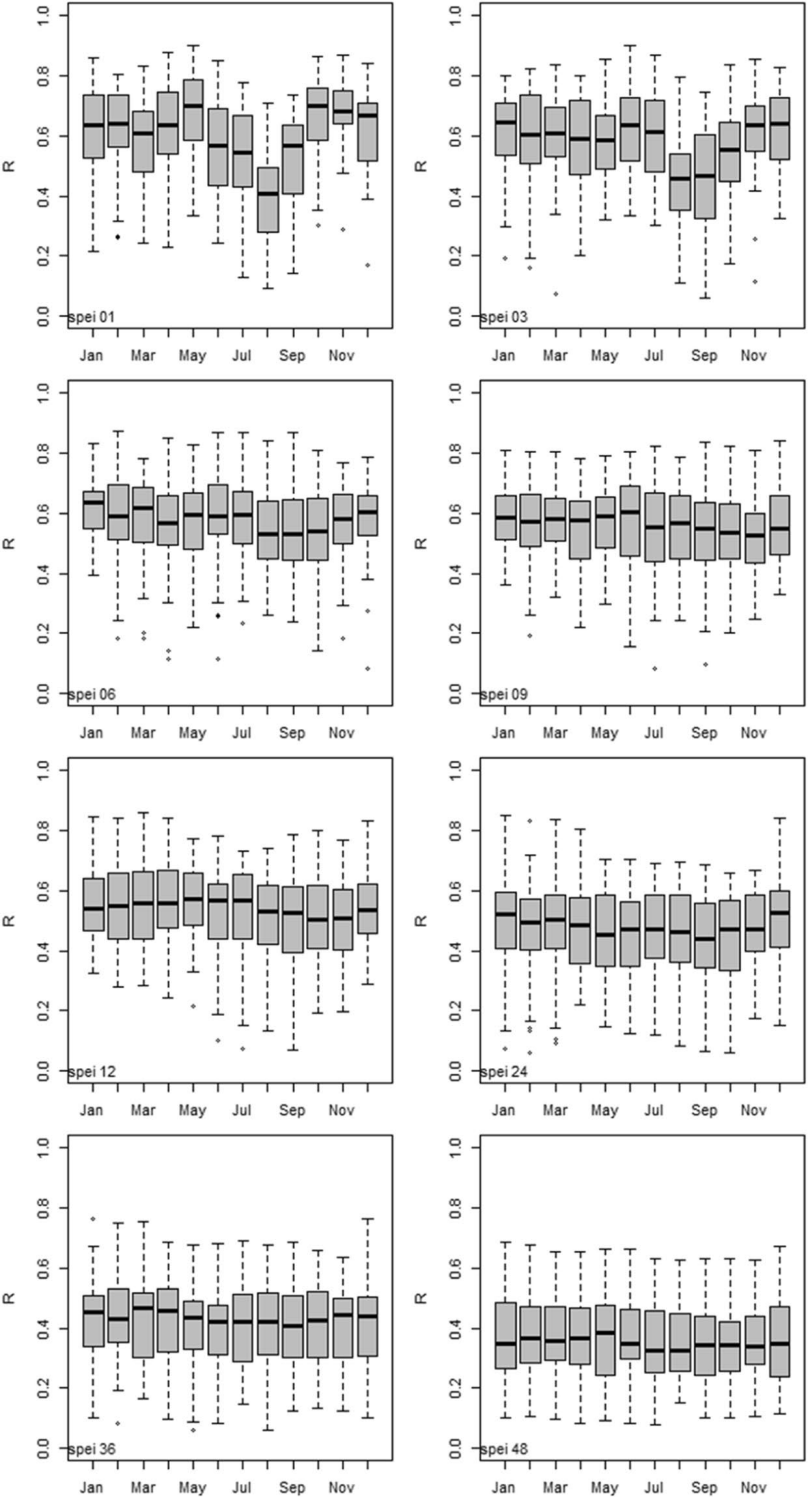
Statistically significant (p < 0.05) spatial correlation between SPEI-HR and SPEI-CRU at different time scales for each month represented using a box and whiskers plot. The spatial correlation was estimated using Pearson’s correlation and R is the Pearson correlation coefficient.

To assess the difference between SPEI-HR and SPEI-CRU spatial and temporal correlation were evaluated using Pearson’s coefficient for time scales 1, 3, 6, 9, 12, 24, 36, and 48. Figure 3 presents the results from temporal analysis, where the time series of each pixel for a certain time scale from SPEI-HR and SPEI-CRU were correlated and the results were plotted on a map. The results from temporal analysis indicate that SPEI-HR and SPEI-CRU for all time scales were highly correlated with a median correlation either equal to 0.6 or greater than 0.6 for all the time scales, as shown in the box and whiskers plot for each time scale in Fig. 3. Even though the correlation values were generally high a diminishing pattern can be observed as the time scale increases. For example, the region around 65°E and 50°N had a high correlation for SPEI 01, but the correlation decreased for greater time scales and eventually we observed negative correlation values for time scales greater than 12. Additionally, the results from spatial correlation analysis, presented in Fig. 4, were estimated by comparing SPEI-HR and SPEI-CRU values for a particular month and then the monthly correlation values over the entire period were used to prepare a box and whiskers plot. The results from spatial analysis indicate that overall, the SPEI-HR and SPEI-CRU agreed well with each other due to positive correlations (R > 0.3), but the median correlation decreased as the time scale increased. Furthermore, except for SPEI 01 and SPEI 03, we observed similar correlation values between different months for each time scale. The relatively low correlations for the month of August and September for SPEI 01 and SPEI 03 could be attributed to the short accumulation periods of the water deficit for these time scales.

It needs to be highlighted that only statistically significant correlation values, which have p-values less than 0.05, were used in evaluating the new SPEI-HR dataset.

### SPEI-HR and SPEI-CRU comparison against root zone soil moisture (RSM)

**Fig. 5:**
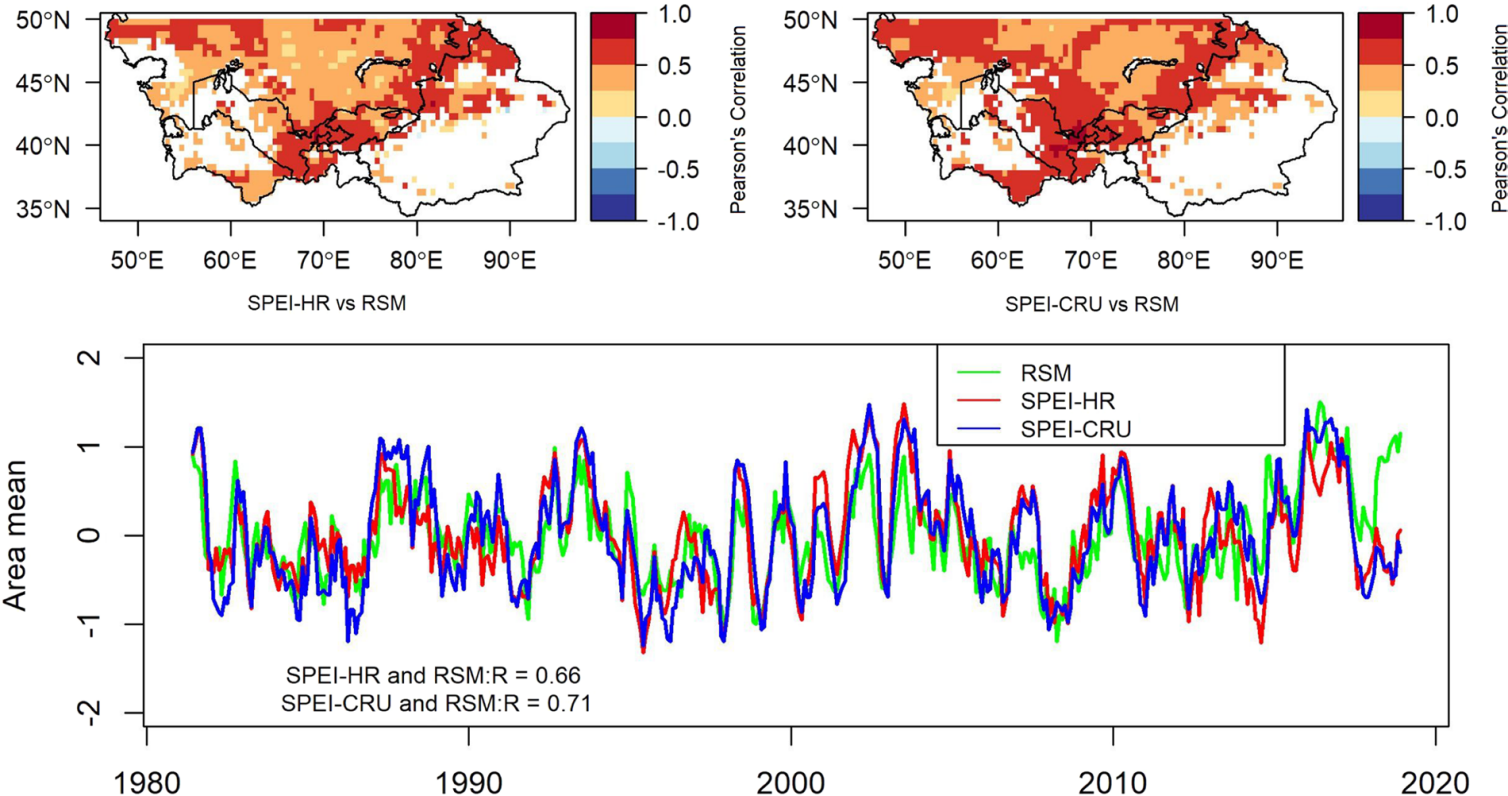
Statistically significant (p < 0.05) correlation maps for evaluating differences between SPEI, with a time scale of 6 month, and RSM. The top left map correlates high-resolution (HR) SPEI-06 with RSM, while the top right map correlates the coarse resolution (CRU) SPEI-06 with RSM. The time series plot shows the variations in area mean (or spatial average) of 6-month SPEI-HR, SPEI-CRU, and RSM against time.

To assess the performance of the new dataset it was compared with standardized root zone soil moisture from GLEAM. As shown in Fig. 5, the SPEI with a time scale of 6-month was evaluated with RSM and the results indicate that even though both SPEI-HR and SPEI-CRU gave positive correlation values with RSM, the performance of SPEI-CRU was slightly better, specifically in centre of the map (70°E, 40°N to 45°N). Furthermore, the time series of area mean, which was calculated by estimating the spatial average of each month, showed that SPEI-HR and SPEI-CRU gave similar results, whereas RSM followed the general trend, but was slightly different than both sets of SPEI. The area mean correlation between SPEI-HR and RSM was 0.66 and between SPEI-CRU and RSM was 0.71, a difference of 7.04 percent. The results given in Fig. 5 were only for a 6-month time scale, therefore, area mean correlation between both sets of SPEI and RSM was estimated for seven other time steps to explore their correlations, as presented in Table 3. The results from Table 3 show that SPEI-HR agrees best with RSM at 6-month time scale. Since deficiency in RSM is an indicator for agricultural drought^14^, therefore, to monitor an agricultural drought SPEI-HR with 6-month time scale could be a more statistically reasonable solution in Central Asia.

**Table 3:** Statistically significant (p < 0.05) correlation within area mean SPEI, at different time scales, and area mean of RSM.

	SPEI-01	SPEI-03	SPEI-06	SPEI-09	SPEI-12	SPEI-24	SPEI-36	SPEI-48
R (SPEI-CRU)	0.5	0.72	0.71	0.62	0.57	0.53	0.47	0.39
R (SPEI-HR)	0.33	0.62	0.66	0.59	0.53	0.47	0.35	0.26

### SPEI-HR and SPEI-CRU comparison against NDVI

**Fig. 6:**
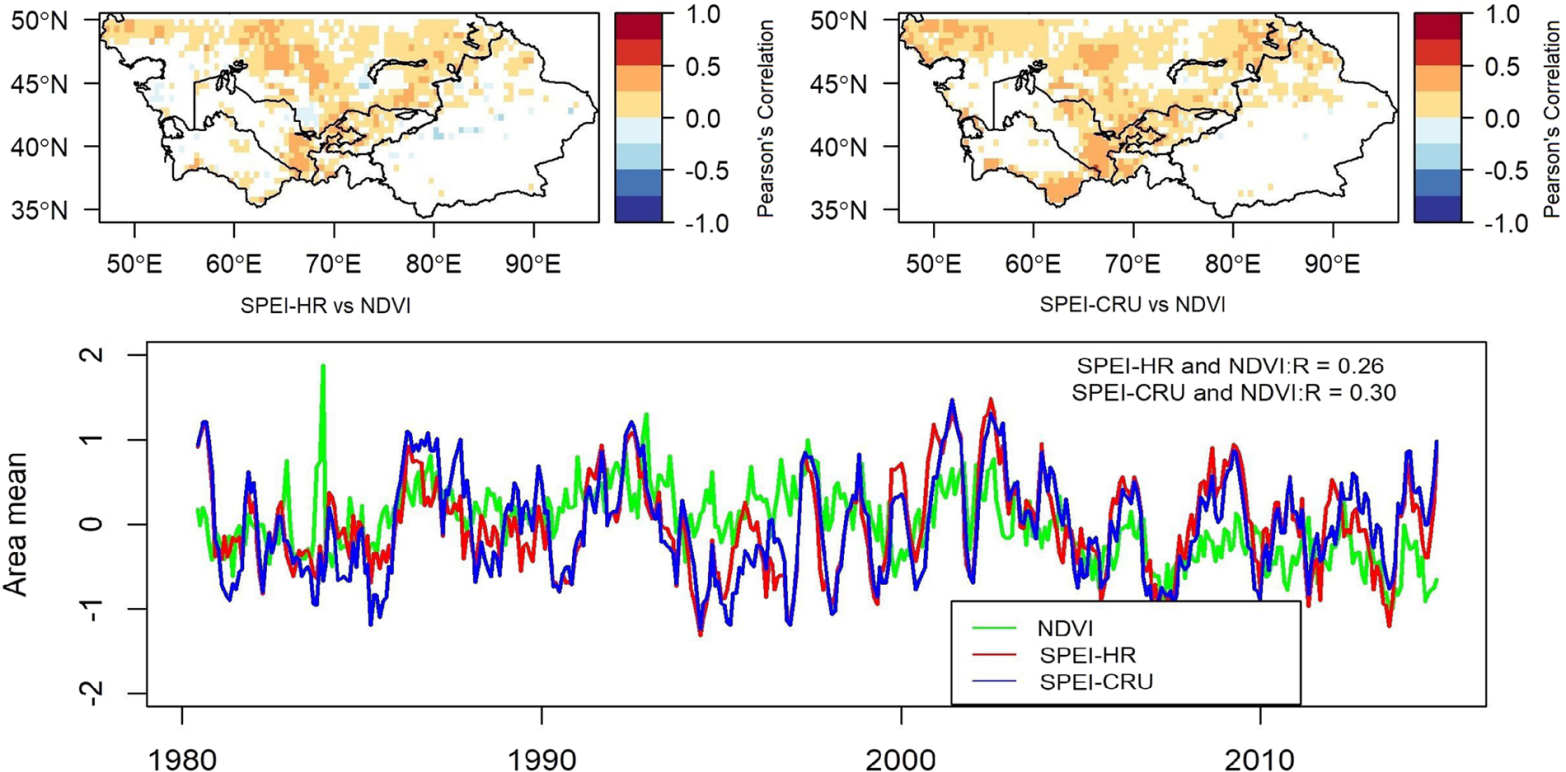
Statistically significant (p < 0.05) correlation maps for evaluating differences between SPEI, with a time scale of 6 month, and NDVI. The top left map correlates high-resolution (HR) SPEI-06 with NDVI, while the top right map correlates the coarse resolution (CRU) SPEI-06 with NDVI. The time series plot shows the variations in area mean (or spatial average) of 6-month SPEI-HR, SPEI-CRU, and NDVI against time.

To further assess the performance of the SPEI-HR it was compared with the GIMMS’s standardized NDVI product. NDVI values indicate the health of a vegetation and have been previously used for monitoring droughts^14^. Therefore, they were correlated with SPEI values. Figure 6 presents the result of correlation between a 6-month time scale SPEI-HR and SPEI-CRU against NDVI. The emerging pattern shows that although largely similar, the performance of SPEI-CRU was slightly better than SPEI-HR. Interestingly, there were some negative correlations in both datasets, but they were more dominant in SPEI-HR. In general, there were very few high correlations and most correlation values lie below 0.5. The low correlations values could be attributed to complex seasonal processes that vegetation goes through annually. Furthermore, the growth of vegetation depends on multiple variables and not just water availability, which is the only variable required for SPEI estimation. Slightly better values were observed for the African continent by Jian Peng *et al*.^15^. The timeseries in Fig. 6 also presents low correlation values for the area mean values for both SPEI products against NDVI, supporting the results of the spatial correlation plots. The correlation between 6-month SPEI-HR and SPEI-CRU against NDVI were 0.26 and 0.30, respectively. According to Table 4 the time scales with highest correlation were 6, 9, and 12-month.

**Table 4:** Statistically significant (p < 0.05) correlation within area mean SPEI, at different time scales, and area mean of NDVI.

	SPEI-01	SPEI-03	SPEI-06	SPEI-09	SPEI-12	SPEI-24	SPEI-36	SPEI-48
R (SPEI-CRU)	0.12	0.23	0.30	0.31	0.30	0.20	0.08*	0.05*
R (SPEI-HR)	0.03*	0.15	0.26	0.31	0.29	0.21	0.1*	0.04*

Note: The asterisk (*) indicates correlation coefficient vale has a p-value greater than 0.05 and is not statistically significant.

### Pattern characteristics of SPEI, RSM, and NDVI for certain drought events

Guo *et al*.^2^ found that Central Asia has suffered three periods of severe droughts in the last fifty years, which are 1973–1979, 1983–1988, and 1997–2003. Furthermore, in the same research the authors identified that there were noticeable drought events in some clustered regions during 2001 and 2008. Furthermore, FAO and World Food Program (WFP) reported that during the severe drought event of 2001 the regional agricultural industry incurred damages worth of US$800 million, the precipitation and river discharge levels were below average by 60–40% and 40–35%, respectively. While during the drought event of 2008, six percent of the population of Kyrgyzstan fell below poverty line and the wheat harvest in Tajikistan was down by 20–35%^50,51^.

**Fig. 7:**
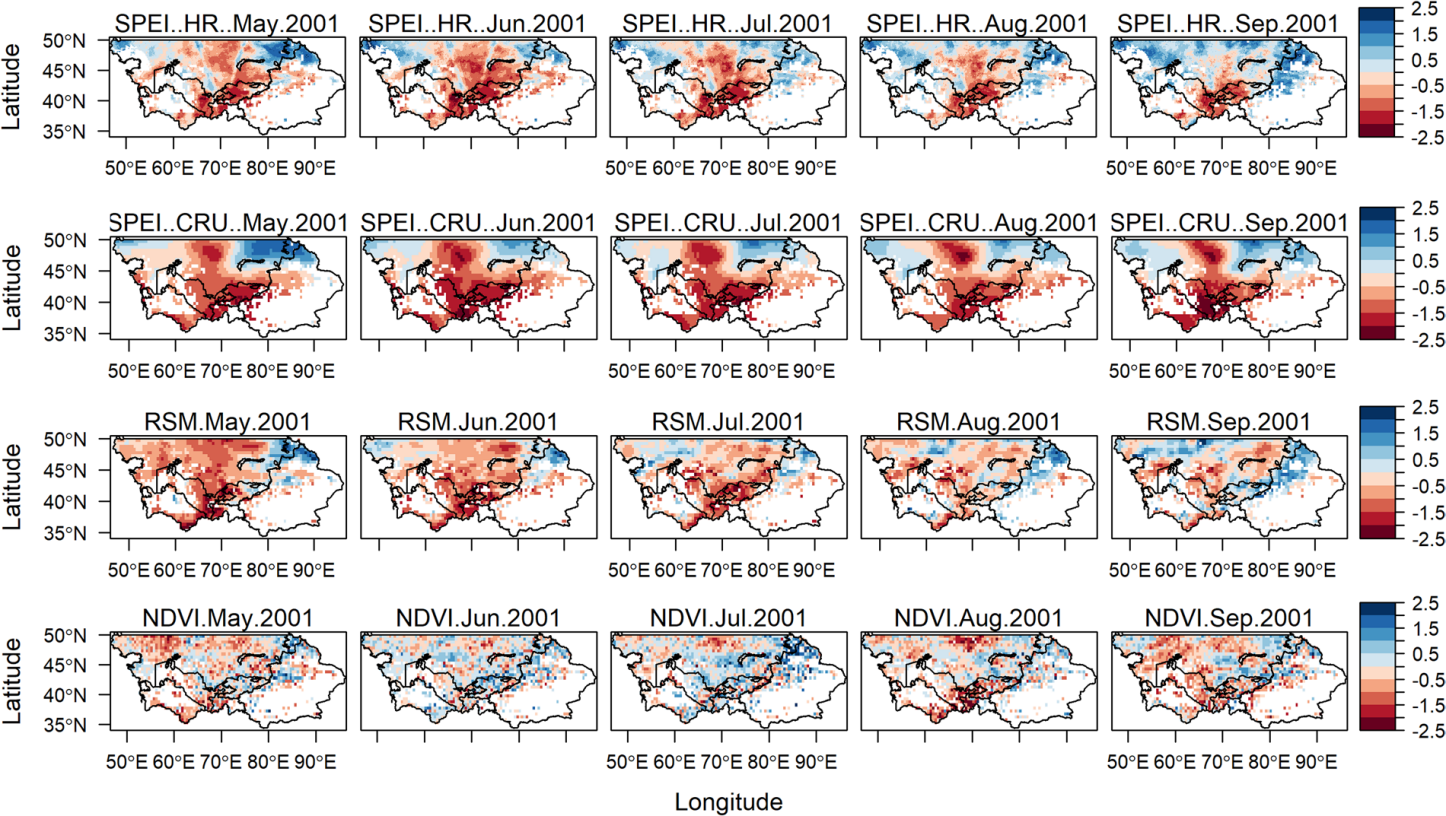
Propagation of spatial patterns for 6-month SPEI-HR, SPEI-CRU, RSM, and NDVI from May to September 2001.

**Fig. 8:**
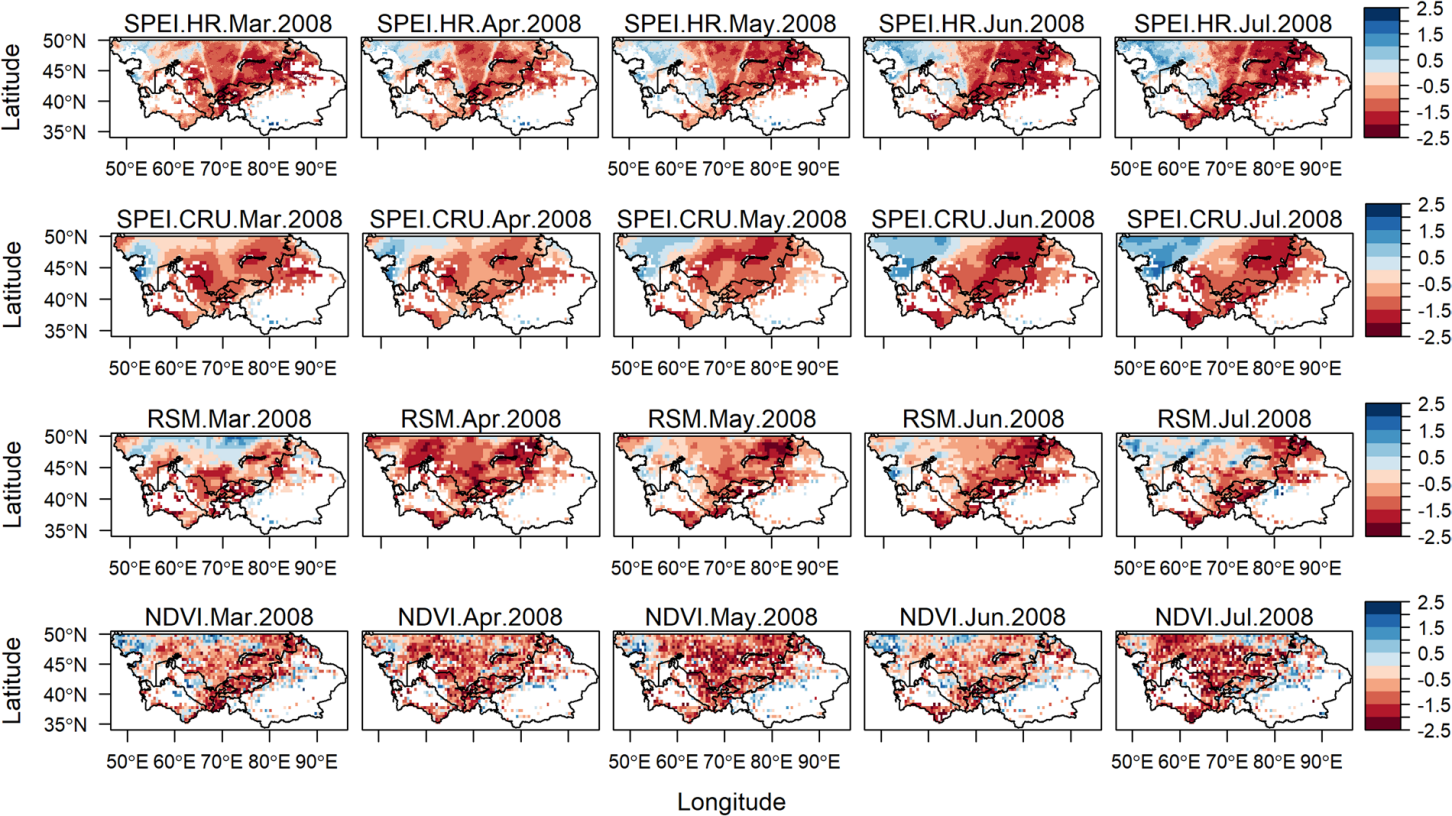
Propagation of spatial patterns for 6-month SPEI-HR, SPEI-CRU, RSM, and NDVI from March to July 2008.

To evaluate the performance of the new high-resolution SPEI dataset a direct comparison was conducted between 6-month SPEI-HR, SPEI-CRU, RSM, and NDVI for the year 2001 (May-September) and 2008 (March-July). As shown in Figs. 7 and 8, it is evident that the SPEI-HR dataset was able to observe similar drought patterns over time and space when compared to low resolution SPEI-CRU. The slight differences, specifically for the 2001 event, could be attributed to different precipitation data and E_p_ estimation methods used by the two datasets. Further comparison revealed that the evolution of 6-month SPEI-HR for both years was very well reflected in RSM. Concerning the relationship between 6-month SPEI-HR and NDVI it can be seen that the connection or reflection was very strong for the drought event of 2008, while it was slightly weak for the 2001 drought event. Overall, the four variables successfully demonstrated the progressive drying-out of Central Asia for both events. During the 2001 event the central and southern regions experienced the most severe events, the intensity of drought started to reduce in September 2001. Whereas, for the event of 2008 almost the entire Central Asia was experiencing either severe or extreme drought, except for the small part of north-western region, this event seemed to be less severe than 2001, but more spatially spread and did not seem to ease off for the whole period of observation. Another set of plots were produced using the SPEI threshold provided by Danandeh Mehr *et al*.^52^, these plots are available in appendix as Figure A3 and Figure A4. The overall drought patterns were similar under both thresholds therefore we opted for the more commonly used SPEI threshold. In future a site-specific stand-alone study might be required to choose the most suitable SPEI threshold for Central Asia.

The results from Figs. 7 and 8 indicate that the SPEI-HR dataset is able to capture drought events. Therefore, the dataset could be used to assess different impacts of droughts and to study atmospheric processes on a finer scale.

## Usage Notes

### Emerging hot spot analysis

**Fig. 9:**
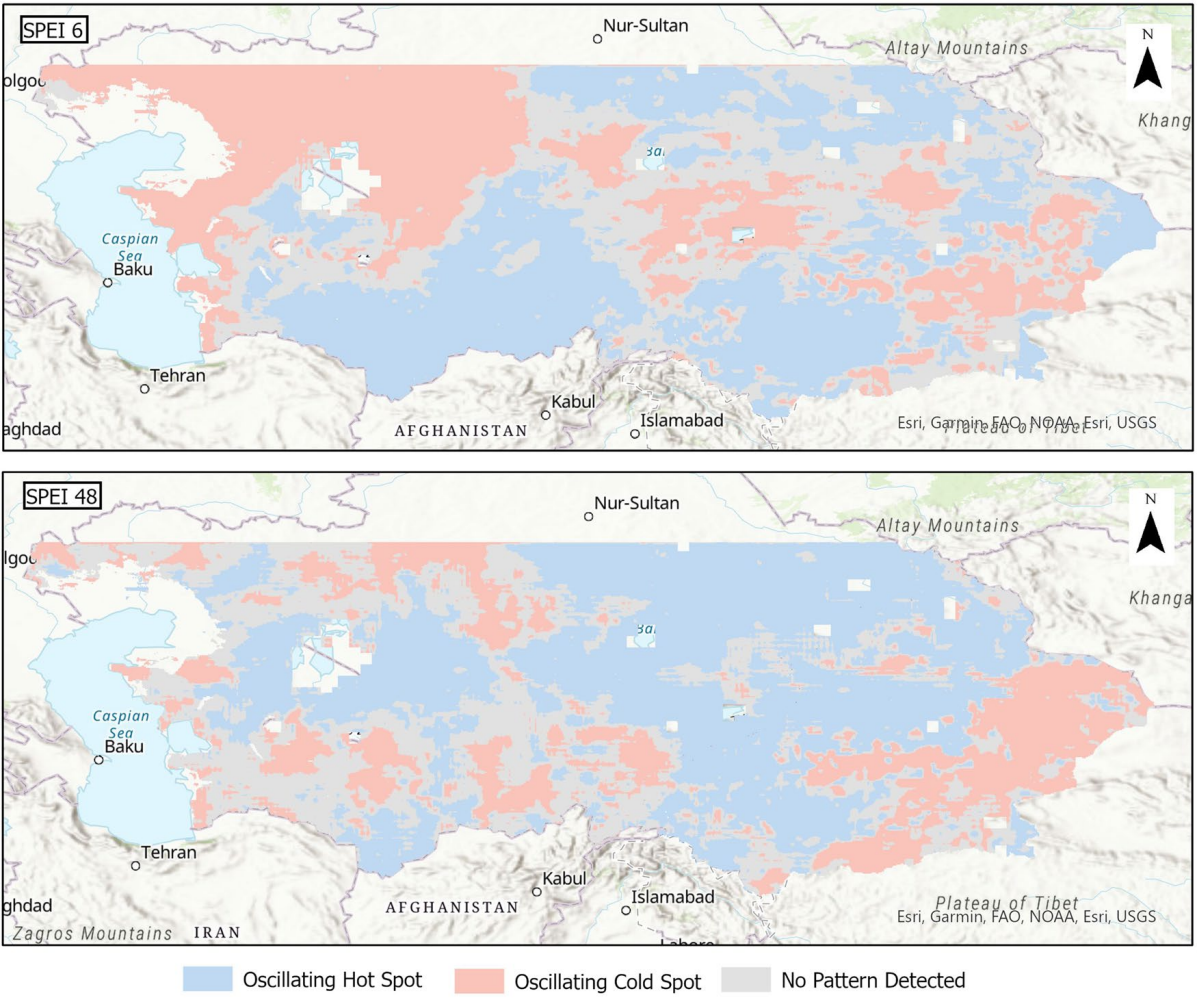
Emerging Hot Spot Analysis using 6-month and 48-month SPEI-HR. In this figure a hot spot, shaded in blue, indicates a region with wet conditions, while a cold spot, shaded in red, indicates a region with dry conditions.

Figure 9 and Figure A1-A2 (in the Appendix) presents the results of emerging hot spot analysis from multiple time scales of SPEI-HR dataset. The analysis was conducted for the entire Central Asia and barren and non-vegetated lands were not masked. The reason of conducting this analysis was to observe and identify regions with varying patterns. As shown in Fig. 9, the 6-month time scale was used to observe short term hot and cold spots, which could give us information regarding agricultural droughts, their trends, and intensities, while the long term 48-month time scale was used to understand how the water deficit of the region is shaping up over a four-year period at a basin and district scale. A positive or a high SPEI value indicates a wet region therefore a hot spot in this context indicates a region with sufficient water, while cold spot indicates a dry region.

In this study, for eight different time scales, only three patterns emerged: 1) Oscillating hot spot, which is shaded in blue, represents wet condition in the last time step and indicates that the region is a hot spot for less than 90% of the time with a tendency and a history of being a cold spot. 2) Oscillating cold spot, which is shaded in red, represents dry condition in the last time step and indicates that the region is a cold spot for less than 90% of the time with a tendency and a history of being a hot spot. 3) No detectable pattern, which is shaded in grey. Other patterns like persistent, new, intensify, diminishing, consecutive and sporadic hot or cold spots did not emerge possibly because of the seasonal nature of the SPEI values and the very fine resolution of our dataset which may lead to a very high local variation.

As presented in Fig. 9, in the 6-month time scale, the north-western region and parts of central and south-eastern regions indicated an oscillating cold spot therefore were going through dry conditions, while the rest of the Central Asia had wet conditions. The results for the 48-month time step showed that, unlike SPEI-6, the droughts in central and western regions were affecting a smaller area and most of Central Asia seemed to have wet conditions.

## Supplementary information

Supplementary Information

## Data Availability

Climate Data Operators from Max Planck Institute of Meteorology was used in the pre-processing of the data. Then functions of the SPEI package in R programming Language were used to prepare the final code. The code files are provided to the journal as Supplementary Information.
