# Supplementary material for: Development and application of high resolution SPEI drought dataset for Central Asia
Source: Sci Data. 2022 Apr 14;9:172. doi: 10.1038/s41597-022-01279-5 (PMC9010421; doi:10.1038/s41597-022-01279-5)
Supplement: Supplementary file 1 — Supplementary Information [file 41597_2022_1279_MOESM1_ESM.pdf]

Supplementary Document

Table of Contents

1. Appendix 2
2. Code Description 6
   a. Read me 6
3. Code / Script 8
   a. Preprocessing 8
      i. Climate Data Operator – Codes 8
   b. Main processing 9
      i. ncf_D_spei_prep_rev03_scidata 9
   c. Postprocessing 12
      i. corr_analysis_spei1_sci 12
      ii. corr_analysis_spei3_sci 17
      iii. corr_analysis_spei6_sci 22
      iv. corr_analysis_spei9_sci 27
      v. corr_analysis_spei12_sci 32
      vi. corr_analysis_spei24_sci 37
      vii. corr_analysis_spei36_sci 42
      viii. corr_analysis_spei48_sci 46
      ix. soilM_spatio_corr_sci 52
      x. ndvi_rev03_sci 56
      xi. standardize_ndvi_sci 63
      xii. standardize_soilmoisture_sci 64
      xiii. Functions 65

1. Appendix

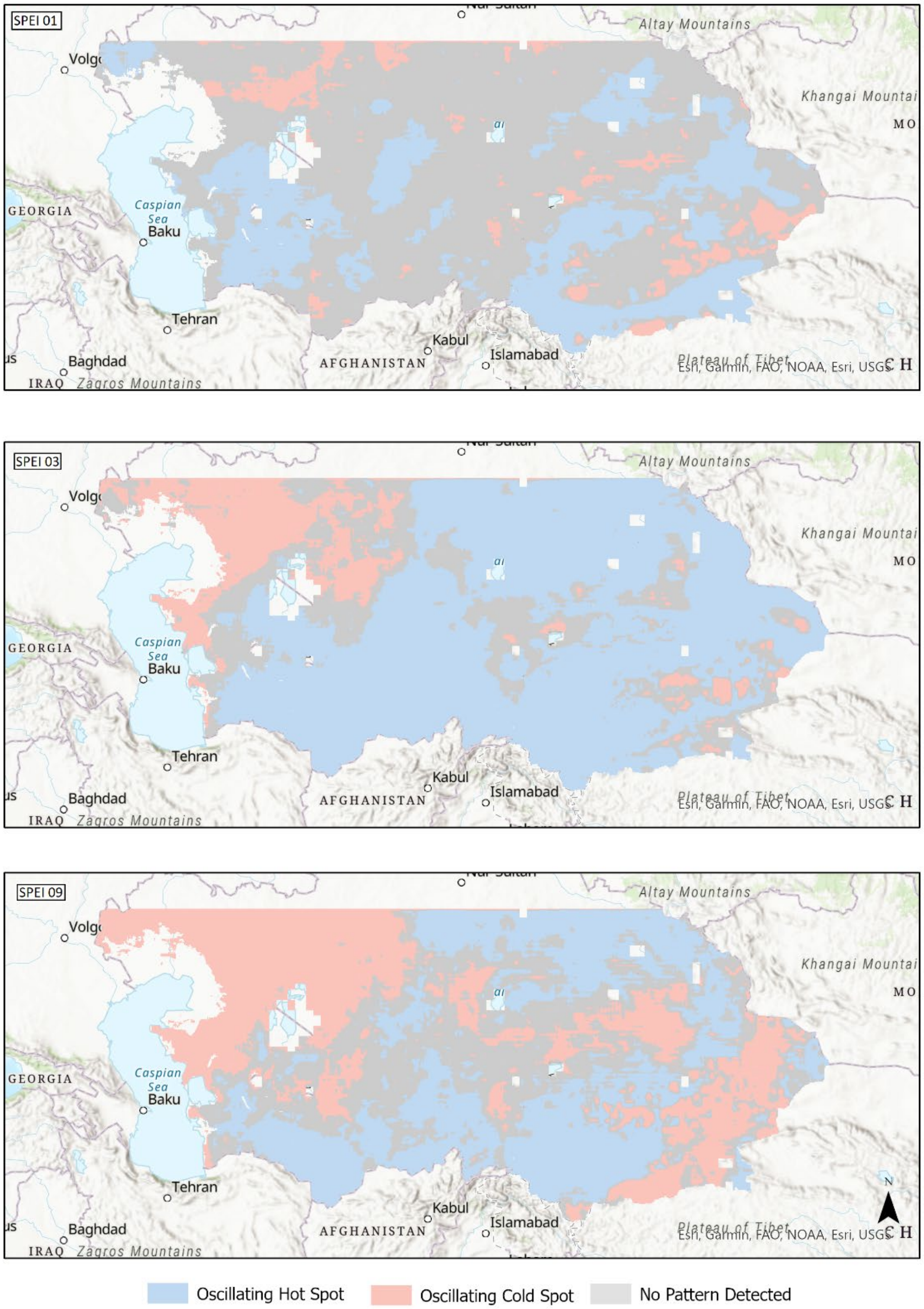

Figure A 1: Emerging Hot Spot Analysis using 1-month, 3-month and 9-month SPEI-HR.

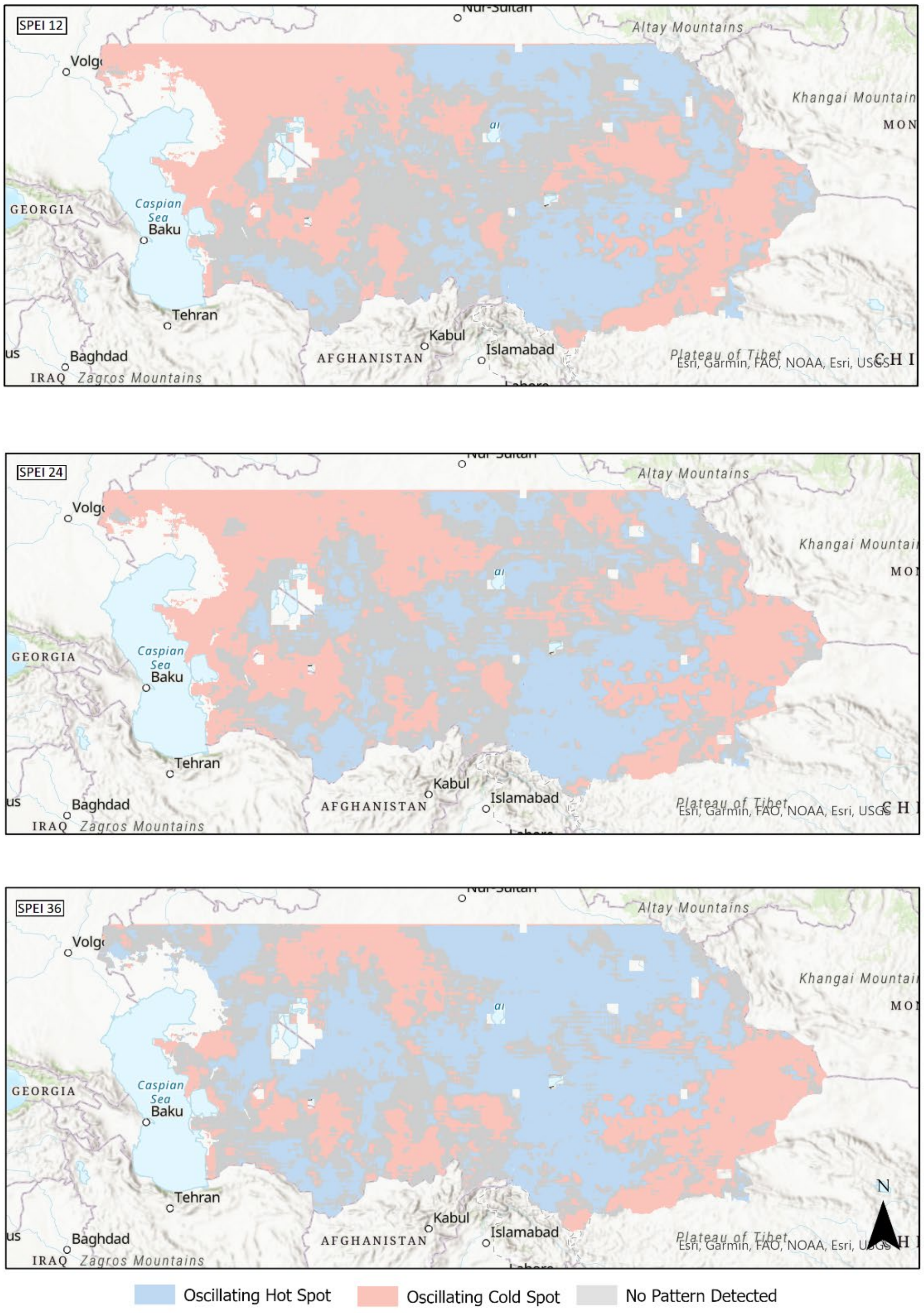

Figure A 2: Emerging Hot Spot Analysis using 12-month, 24-month and 36-month SPEI-HR.

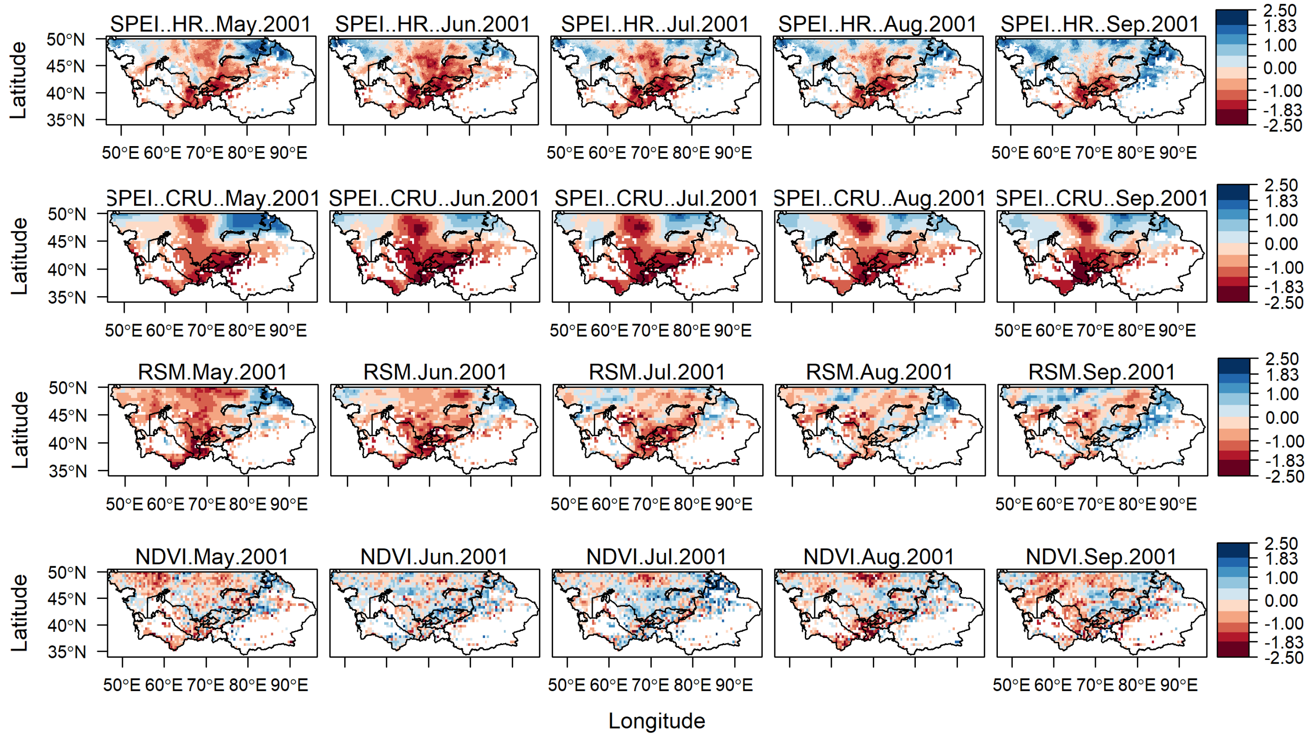

Figure A 3: Propagation of spatial patterns for 6-month SPEI-HR, SPEI-CRU, RSM, and NDVI from May to September 2001 using the new range defined by Mehr et al. 2020.

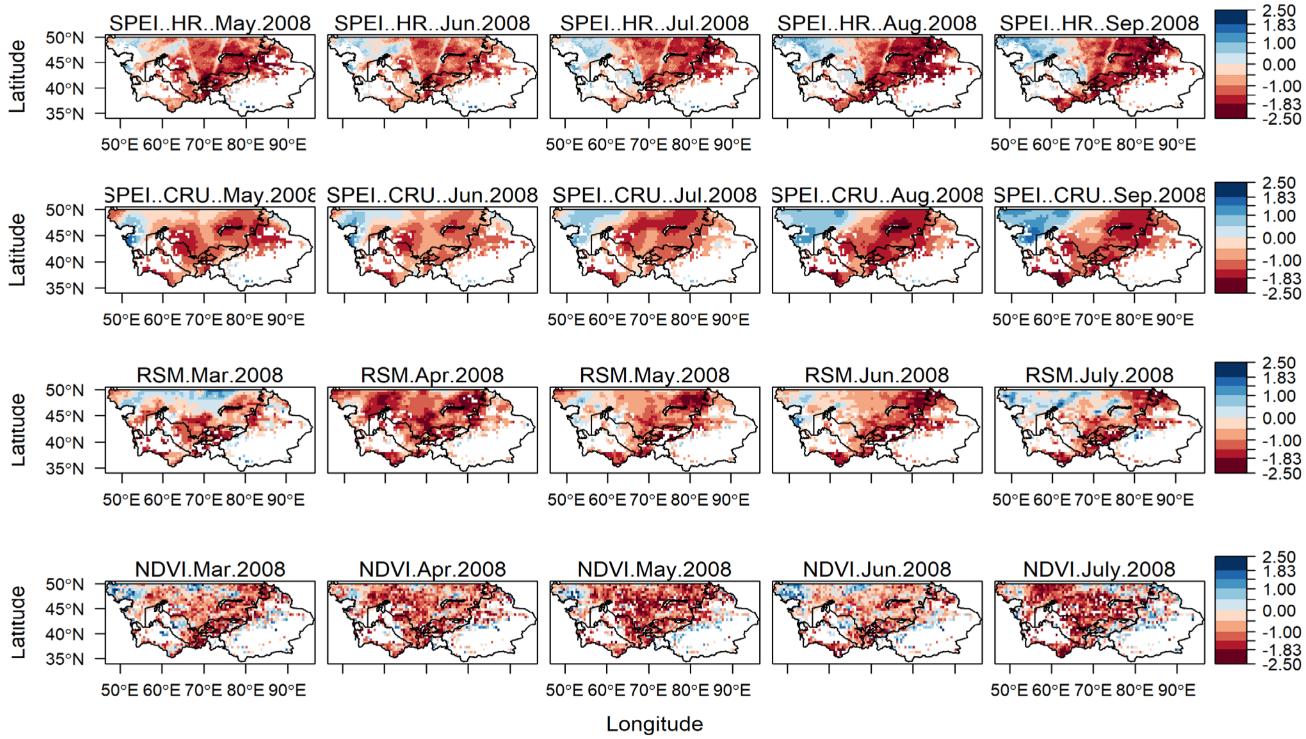

Figure A 4: Propagation of spatial patterns for 6-month SPEI-HR, SPEI-CRU, RSM, and NDVI from March to July 2008 using the new range defined by Mehr et al. 2020.

2. Code Description

a. Read me

The different R scripts or codes prepared for this study are split into three categories: pre-processing, main-processing and post processing.

In the “pre-processing”, we provide all the commands used for pre-processing raw data in the pdf document titled “Preprocessing CDO commands”. The final output is a water deficit NetCDF dataset which will serve as an input data for the main processing.

In the “main-processing”, the R script used for developing the SPEI dataset is available with the name “ncf_D_spei_prep_rev03_scidata”. This script was run 48 times, once for each time scale. The script imports the water Deficit database in NetCDF format and gives a SPEI database in NetCDF format, depending on the timescale used.

In the “post-processing”, there are different R scripts, and each script was written to perform specific tasks. The name of the scripts and their tasks are described as following:

- R scripts named as “corr_analysis_spei#_sci” (# = 1, 3, 9, 12, 24, 36, 48)

  These scripts estimate correlation between different time scales of high resolution SPEI database (developed in this study) and low resolution SPEI database from Climate Research Unit datasets (CRU). The correlation is conducted over time and space.

- “soilM_spatio_corr_sci”

  This script estimates correlation between high resolution SPEI database (developed in this study) and soil moisture dataset from GLEAM and between low resolution SPEI database from CRU and soil moisture dataset from GLEAM. The correlation is conducted over time and space (area mean).

- “ndvi_rev03_sci”

  Similarly, this script estimates correlation between high resolution SPEI database (developed in this study) and normalized difference vegetation index (NDVI) dataset from GIMMS and between low resolution SPEI database from CRU and NDVI dataset from GIMMS. The correlation is conducted over time and space (area mean).

- “standardize_ndvi_sci” and “standardize_soilmoisture_sci”

  These two R functions are written to standardize soil moisture and NDVI data before correlation analysis. These functions are required by scripts “soilM_spatio_corr_sci” and “ndvi_rev03_sci” in the post processing steps.

- Emerging Hotspot Analysis

  To perform the emerging hotspot analysis, we uploaded the high resolution SPEI dataset in a multi-dimensional raster on ArcMap Pro (without code), then we converted the raster into a space time cube and used the following settings to perform the analysis.

- Analysis Variable: SPEI
- Conceptualization of Spatial Relationships: k nearest neighbors
- Number of spatial neighbors: 8
- Neighborhood timestep: 1
- Polygon Analysis Mask: none (left empty)
- Define Global Window: Entire Cube

3. Code / Script

a. Preprocessing

i. Climate Data Operator – Codes

After obtaining the precipitation (P) and potential evapotranspiration (Ep) NetCDF data from Climate Hazards group InfraRed Precipitation with Station's (CHIRPS) and Global Land Evaporation Amsterdam Model's (GLEAM), respectively, we had to make sure that datasets had the same spatial and temporal resolutions. To carry out this task Climate Data Operators, CDO, from Max Planck Institute were used because they can operate directly on NetCDF format.

The GLEAM Ep dataset from 1980 to 2018 was split into years using the following command.

- cdo split Ep_1980_2018_GLEAM_v3.3a_MO.nc year_XXXX.nc

The GLEAM Ep dataset was downscaled from 25km to 5km using the grid from CHIRPS dataset and applying bilinear interpolation.

- cdo remapbil,chirps.grd, year1980.nc year1980_0.05.nc

Then the new high-resolution yearly NetCDF datasets were merged from 1981 to 2018 using the following command.

- cdo -z zip mergetime *.nc Ep_Gleams_0.05_1981-2018.nc

Similarly, the CHIRPS precipitation data was converted from daily to monthly resolution by using the CDO operator monthly sum.

- cdo monsum chirps-v2.0.1995.days_p05.nc chirps-v2.0.1995.mons_p05.nc

The monthly precipitation dataset obtained for each year was then merged from 1981 to 2018.

- cdo -z zip mergetime *.nc chirps-v2.0.1981-2018.mons_p05.nc

Both precipitation and Ep datasets had either global or quasi global coverage, therefore, they were masked using a NetCDF shapefile for our study area by applying the following commands.

- cdo div Ep_Gleams_0.05_1981-2018.nc CA_mask.nc CA_Ep_Gleams_0.05_1981-2018.nc
- cdo div chirps-v2.0.1981-2018.mons_p05.nc CA_mask.nc CA_chirps-v2.0.1981-2018.mons_p05.nc

The final steps of preprocessing were to evaluate the water deficit by subtracting potential evapotranspiration from precipitation and to reduce the size of the dataset by selecting the data within a spatial box. This was carried out using the following commands.

- cdo -z zip sub CA_chirps-v2.0.1981-2018.mons_p05.nc CA_Ep_Gleams_0.05_1981-2018.nc CA_Deficit_0.05_1981-2018.nc

- cdo -z zip_5 sellonlatbox 46.52499, 96.375, 34.375, 49.975 CA_Deficit_0.05_1981-2018.nc CA_latlonbox_Deficit_0.05_1981-2018.nc

To estimate Standardized Precipitation Evapotranspiration Index (SPEI) the water deficit dataset produced here will be used by the code titled “ncf_D_spei_prep_rev03_scidata”, which is provided in the folder named “main-processing”.

b. Main processing

i. ncf_D_spei_prep_rev03_scidata

```
library(ncdf4)
# Import Water Defict NetCDF data generated during preprocessing --------
# set path and filename
ncpath <- "E:/Thesis_2020/Analysis/Preprocessing/Deficit_values/"
ncname <- "CA_latlonbox_Deficit_0.05_1981-2018"
ncfname <- paste(ncpath, ncname, ".nc", sep="")
dname <- "precip" # note: precip means deficit (not precipitation)
# open a netCDF file
ncin <- nc_open(ncfname)
#print(ncin)
# get longitude and latitude
lon <- ncvar_get(ncin,"longitude")
nlon <- dim(lon)
#head(lon)
lat <- ncvar_get(ncin,"latitude")
nlat <- dim(lat)
#head(lat)
#print(c(nlon,nlat))
# get time
time <- ncvar_get(ncin,"time")
#time
tunits <- ncatt_get(ncin,"time","units")
nt <- dim(time)
nt
tunits
# get deficift
D_array <- ncvar_get(ncin,dname)
dlname <- ncatt_get(ncin,dname,"long_name")
dunits <- ncatt_get(ncin,dname,"units")
fillvalue <- ncatt_get(ncin,dname,"_FillValue")
dim(D_array)
# get global attributes
title <- ncatt_get(ncin,0,"title")
institution <- ncatt_get(ncin,0,"institution")
datasource <- ncatt_get(ncin,0,"source")
references <- ncatt_get(ncin,0,"references")
history <- ncatt_get(ncin,0,"history")
Conventions <- ncatt_get(ncin,0,"Conventions")
nc_close(ncin)
# load following packages
library(chron)
library(lattice)
library(RColorBrewer)
# formatting time and replacing fill values with NA ----------------------
# convert time -- split the time units string into fields
tustr <- strsplit(tunits$value, " ")
```

```
tdstr <- strsplit(unlist(tustr)[3], "-")
tmonth <- as.integer(unlist(tdstr)[2])
tday <- as.integer(unlist(tdstr)[3])
tyear <- as.integer(unlist(tdstr)[1])
chron(time,origin=c(tmonth, tday, tyear))
# replace netCDF fill values with NA's
D_array[D_array==fillvalue$value] <- NA
length(na.omit(as.vector(D_array[,,1])))
# quick map of imported data ---------------------------------------------
# get a single slice or layer (January)
#m <- 1
#D_slice <- D_array[,,m]
#image(lon,lat,D_slice, col=rev(brewer.pal(10,"RdBu")))
# levelplot of the slice
#grid <- expand.grid(lon=lon, lat=lat)
#cutpts <- c(-50,-40,-30,-20,-10,0,10,20,30,40,50)
#cutpts <- c(-200,-150,-100,-50,0,50,100,150,200)
#levelplot(D_slice ~ lon * lat, data=grid, at=cutpts, cuts=11, pretty=T,
# col.regions=(rev(brewer.pal(10,"RdBu"))))
# reshape array into dataframe --------------------------------------------
# First: reshape the array into vector
D_vec_long <- as.vector(D_array)
length(D_vec_long)
# then reshape the vector into a matrix
D_mat <- matrix(D_vec_long, nrow=nlon*nlat, ncol=nt)
dim(D_mat)
#head(na.omit(D_mat))
# create a dataframe and insert the D_mat matirx
lonlat <- as.matrix(expand.grid(lon,lat))
D_df02 <- data.frame(cbind(lonlat,D_mat))
#names(D_df02) <- c("lon","lat",)
#head(na.omit(D_df02, 2))
# create a dataframe without missing values. This will be used later to
demonstrate how to convert a "short"
# data frame into full matrix or array for writing out as a netCDF file.
D_df02 <- na.omit(D_df02)
#head(D_df02,2)
# remove lon and lat columns and work on new data frame
Var1 <-D_df02$Var1
Var2 <-D_df02$Var2
drops <- c("Var1","Var2")
dim(D_df02)
D_df02 <- D_df02[ , !(names(D_df02) %in% drops)]
D_df03 <- as.data.frame(t(D_df02))
D_df04 <- D_df03
# --------------- Perform SPEI Here ----------------------
# Perform SPEI Here #
library(SPEI)
for(i in seq(1,205000,1000)){
j=i+999
D_df04_a <- D_df03[,i:j]
spei12_a <- spei(D_df04_a, 60)
D_df04_a <- as.data.frame(spei12_a$fitted[,1:1000])
D_df04[,i:j] <- D_df04_a
print(sprintf("i = %i and j = %i", i,j))
}
# the 277 remaining spei calculations
D_df04_a <- D_df03[,205001:205277]
```

```
spei24_a <- spei(D_df04_a, 60)
D_df04_a <- as.data.frame(spei24_a$fitted[,1:277])
D_df04[,205001:205277] <- D_df04_a
#Add longitude and latitude back to the dataframe
spei_df05 <-t(D_df04)
spei_df05 <- data.frame(lon=Var1,lat=Var2,spei_df05)
spei_df06 <- spei_df05
remove(D_array)
remove(D_vec_long)
remove(D_mat)
remove(D_df02)
remove(D_df03)
remove(D_df04)
remove(spei_df05)
# ------- Convert a "short e.g spei_D_df06" R data fram to an array ------
--
# copy lon, lat and time from initial netCDF data set
lon4 <- lon
lat4 <- lat
time4 <- time
tunits4 <- tunits
nlon4 <- nlon; nlat4 <- nlat; nt4 <- nt
# create arrays
# nlon * nlat * nt array
fillvalue <- 1e32
spei_array3 <- array(fillvalue, dim=c(nlon4,nlat4,nt4))
# ----------------partial loop avoidance---------------
# loop-avoidance approaches
# get vectors of the grid-cell indices for each row in the data frame
#ptm <- proc.time()
j2 <- sapply(spei_df06$lon, function(x) which.min(abs(lon4-x)))
k2 <- sapply(spei_df06$lat, function(x) which.min(abs(lat4-x)))
fillvalue <- 1e32
# partial loop avoidance for tmp_array3
temp_array <- array(fillvalue, dim=c(nlon4,nlat4))
nobs <- dim(spei_df06)[1]
for (l in 1:nt) {
temp_array[cbind(j2,k2)] <- as.matrix(spei_df06[1:nobs,l+2])
spei_array3[,,l] <- temp_array
}
# --------------- Create NETCDF from the array ---------------
# path and file name, set dname
ncpath <-
"E:/Thesis_2020/Analysis/Preprocessing/Deficit_values/spei_netcdf/"
ncname <- "spei_60"
ncfname <- paste(ncpath, ncname, ".nc", sep="")
dname <- "spei_60" # note: spei means Standardized Precipitation
Evapotranspiration Index
# create and write the netCDF file -- ncdf4 version
# define dimensions
londim <- ncdim_def("lon","degrees_east",as.double(lon4))
latdim <- ncdim_def("lat","degrees_north",as.double(lat4))
timedim <- ncdim_def("time",tunits4$value,as.double(time4))
# define variables
fillvalue <- 1e32
dlname <- "Standardized Precipitation Evapotranspiration Index"
spei_def <- ncvar_def("spei","z
value",list(londim,latdim,timedim),fillvalue,dlname,prec="double")
```

```
# create netCDF file and put arrays
ncout <- nc_create(ncfname,list(spei_def),force_v4=TRUE)
# put variables
ncvar_put(ncout,spei_def,spei_array3)
# put additional attributes into dimension and data variables
ncatt_put(ncout,"lon","axis","X") #,verbose=FALSE) #,definemode=FALSE)
ncatt_put(ncout,"lat","axis","Y")
ncatt_put(ncout,"time","axis","T")
# add global attributes
ncatt_put(ncout,0,"title",'SPEI for Central Asia 1981 - 2018')
ncatt_put(ncout,0,"institution",'Technical University of Munich')
ncatt_put(ncout,0,"source",'CHIRPS for Precipitation and GLEAMS for
Potential Evaporation')
ncatt_put(ncout,0,"references",references$value)
history <- paste("K.Pyarali", date(), sep=", ")
ncatt_put(ncout,0,"history",history)
ncatt_put(ncout,0,"Conventions",Conventions$value)
# Get a summary of the created file:
ncout
```

c. Postprocessing

i. corr_analysis_spei1_sci

```
library(ncdf4)
library(Hmisc)
library(wesanderson)
#----------------------------------------Spatial and Temporal Correlation
Analysis----------------------------------
#-----------------------------------------------------Spatial
Correlation------------------------------------------
#collect data from resample HRU netcdf
# set path and filename
#ncpath <- "C:/Thesis2020/corr_analysis/"
ncpath <- "C:/Thesis2020/corr_analysis/corr_data/hru/"
#ncname <- "spei_1_hru_50km"
ncname <- "spei_1_hru_50km_green"
ncfname <- paste(ncpath, ncname, ".nc", sep="")
dname <- "spei" # note: precip means deficit (not precipitation)
# open a netCDF file
ncin <- nc_open(ncfname)
#print(ncin)
# get longitude and latitude
lon_hru <- ncvar_get(ncin,"lon")
nlon_hru <- dim(lon_hru)
head(lon_hru)
lat_hru <- ncvar_get(ncin,"lat")
nlat_hru <- dim(lat_hru)
head(lat_hru)
#print(c(nlon,nlat))
# get time
time_hru <- ncvar_get(ncin,"time")
#time_hru
tunits_hru <- ncatt_get(ncin,"time","units")
nt_hru <- dim(time_hru)
#nt_hru
tunits_hru
# get spei from hru
spei_hru_array <- ncvar_get(ncin,dname)
```

```
splname_hru <- ncatt_get(ncin,dname,"long_name")
spunits_hru <- ncatt_get(ncin,dname,"units")
fillvalue_hru <- ncatt_get(ncin,dname,"_FillValue")
dim(spei_hru_array)
# get global attributes
#title <- ncatt_get(ncin,0,"title")
#institution <- ncatt_get(ncin,0,"institution")
#datasource <- ncatt_get(ncin,0,"source")
#references <- ncatt_get(ncin,0,"references")
#history <- ncatt_get(ncin,0,"history")
#Conventions <- ncatt_get(ncin,0,"Conventions")
nc_close(ncin)
# collect data from CRU netcdf
# set path and filename
#ncpath <- "C:/Thesis2020/corr_analysis/"
ncpath <- "C:/Thesis2020/corr_analysis/corr_data/cru/"
#ncname <- "spei_cru_1"
ncname <- "spei_cru_1_green"
ncfname <- paste(ncpath, ncname, ".nc", sep="")
dname <- "spei" # note: precip means deficit (not precipitation)
# open a netCDF file
ncin <- nc_open(ncfname)
#print(ncin)
# get longitude and latitude
lon_cru <- ncvar_get(ncin,"lon")
nlon_cru <- dim(lon_cru)
head(lon_cru)
lat_cru <- ncvar_get(ncin,"lat")
nlat_cru <- dim(lat_cru)
head(lat_cru)
#print(c(nlon,nlat))
# get time
time_cru <- ncvar_get(ncin,"time")
#time_cru
tunits_cru <- ncatt_get(ncin,"time","units")
nt_cru <- dim(time_cru)
nt_cru
tunits_cru
# get spei from hru
spei_cru_array <- ncvar_get(ncin,dname)
splname_cru <- ncatt_get(ncin,dname,"long_name")
spunits_cru <- ncatt_get(ncin,dname,"units")
fillvalue_cru <- ncatt_get(ncin,dname,"_FillValue")
dim(spei_cru_array)
# get global attributes
#title <- ncatt_get(ncin,0,"title")
#institution <- ncatt_get(ncin,0,"institution")
#datasource <- ncatt_get(ncin,0,"source")
#references <- ncatt_get(ncin,0,"references")
#history <- ncatt_get(ncin,0,"history")
#Conventions <- ncatt_get(ncin,0,"Conventions")
nc_close(ncin)
# load some packages
library(chron)
library(lattice)
library(RColorBrewer)
# convert time -- split the time units string into fields for HRU and then
CRU
```

```
tustr_hru <- strsplit(tunits_hru$value, " ")
tdstr_hru <- strsplit(unlist(tustr_hru)[3], "-")
tmonth_hru <- as.integer(unlist(tdstr_hru)[2])
tday_hru <- as.integer(unlist(tdstr_hru)[3])
tyear_hru <- as.integer(unlist(tdstr_hru)[1])
chron(time_hru,origin=c(tmonth_hru, tday_hru, tyear_hru))
tustr_cru <- strsplit(tunits_cru$value, " ")
tdstr_cru <- strsplit(unlist(tustr_cru)[3], "-")
tmonth_cru <- as.integer(unlist(tdstr_cru)[2])
tday_cru <- as.integer(unlist(tdstr_cru)[3])
tyear_cru <- as.integer(unlist(tdstr_cru)[1])
chron(time_cru,origin=c(tmonth_cru, tday_cru, tyear_cru))
# replace netCDF fill values with NA's for both HRU and CRU.
spei_hru_array[spei_hru_array==fillvalue_hru$value] <- NA
spei_cru_array[spei_cru_array==fillvalue_cru$value] <- NA
#depending on the spei scale the starting time with available values might
change. So for spei_6 we dont have any values in first 5 layers or slices.
length(na.omit(as.vector(spei_hru_array[,,1])))
length(na.omit(as.vector(spei_cru_array[,,1])))
# get a single slice or layer (January)
m <- 6
spei_hru_slice <- spei_hru_array[,,m]
spei_cru_slice <- spei_cru_array[,,m]
# quick map
#image(lon_hru,lat_hru,spei_hru_slice, col=rev(brewer.pal(10,"RdBu")))
#text(90,35,"hru spei 1")
#image(lon_cru,lat_cru,spei_cru_slice, col=rev(brewer.pal(10,"RdBu")))
#text(90,35,"cru spei 1")
# reshape the arrays into vector
spei_hru_vec_long <- as.vector(spei_hru_array)
length(spei_hru_vec_long)
spei_cru_vec_long <- as.vector(spei_cru_array)
length(spei_cru_vec_long)
# reshape the vectors into a matrix
spei_hru_mat <- matrix(spei_hru_vec_long, nrow=nlon_hru*nlat_hru,
ncol=nt_hru)
dim(spei_hru_mat)
spei_cru_mat <- matrix(spei_cru_vec_long, nrow=nlon_cru*nlat_cru,
ncol=nt_cru)
dim(spei_cru_mat)
#head(na.omit(D_mat))
# create a dataframe
lonlat_hru <- as.matrix(expand.grid(lon_hru,lat_hru))
D_df02_hru <- data.frame(cbind(lonlat_hru,spei_hru_mat))
names(D_df02_hru) <- c("lon","lat")
#head(na.omit(D_df02_hru))
lonlat_cru <- as.matrix(expand.grid(lon_cru,lat_cru))
D_df02_cru <- data.frame(cbind(lonlat_cru,spei_cru_mat))
names(D_df02_cru) <- c("lon","lat")
#head(na.omit(D_df02_cru, 2))
# create a dataframe without missing values. This will be used later to
demonstrate how to convert a "short"
# data frame into full matrix or array for writing out as a netCDF file.
#D_df02 <- na.omit(D_df02_cru)
#head(D_df02,2)
# remove lon and lat columns and work on new data frame for both
# NOTE to Self: "FYI D_df03 is prepared after this step so just make sure
you dont get confusued."
```

```
Var1 <-D_df02_hru$lon
Var2 <-D_df02_hru$lat
drops <- c("lon","lat")
#D_df02 <- D_df03
dim(D_df02_hru)
D_df02_hru <- D_df02_hru[ , !(names(D_df02_hru) %in% drops)]
dim(D_df02_hru)
#drop the first 0 layers due to time scale
#D_df02_hru<-D_df02_hru[,6:456]
#dim(D_df02_hru)
D_df03_hru <- as.data.frame(t(D_df02_hru))
D_df04_hru <- D_df03_hru
Var1 <-D_df02_cru$lon
Var2 <-D_df02_cru$lat
drops <- c("lon","lat")
#D_df02 <- D_df03
dim(D_df02_cru)
D_df02_cru <- D_df02_cru[ , !(names(D_df02_cru) %in% drops)]
dim(D_df02_cru)
#drop the first 0 layers due to time scale
#D_df02_cru<-D_df02_cru[,6:456]
#dim(D_df02_cru)
D_df03_cru <- as.data.frame(t(D_df02_cru))
D_df04_cru <- D_df03_cru
#----------------------------------------Temporal Correlation-------------
-----------------------------
correlation <- data.frame(NA)
data <- data.frame(hru="" ,cru="")
colnames(data) <- c("hru","cru")
for(i in seq(1,3366,1)){
data <- data.frame(hru = D_df04_hru[,i],cru = D_df04_cru[,i])
#data <- data.frame(hru = c(4,2,NA,4,7,8,-Inf,2,7,9,10),cru =
c(1,3,4,NA,3,6,1,3,5,8,-Inf))
#data2 <- data.frame(hru = c(NA,NA,NA,NA,NA,NA,NA,NA,NA,NA,NA),cru =
c(NA,NA,NA,NA,NA,NA,NA,NA,NA,NA,NA))
data <- data[is.finite(rowSums(data)),]
if(nrow(data) != 0){
r <- rcorr(data[,1],data[,2],type=c("pearson"))
if( r$P[2]<0.05){
correlation[i,] <- r$r[2]
}
else{
correlation[i,] <- NA
}
}
else{
correlation[i,] <-NA
}
}
#Add longitude and latitude back to the correlation dataframe
#t_correlation <-t(correlation)
spatial_correlation <- data.frame(lon=Var1,lat=Var2,correlation)
#spei_df05 <-
fread("E:/Thesis_2020/Analysis/Preprocessing/Deficit_values/spei_netcdfspei12.csv"
)#
spei_df05 <- data.frame(spei_df05)
#spei_1_corr_hru_cru_raster<-Raster_it(spatial_correlation)
#writeRaster(spei_1_corr_hru_cru_raster,"spei_1_corr_hru_cru_raster.tif",format="GTiff"
```

```
)
#spei_df06[is.na(spei_df06)] <- 1e32
#corr_array <- array(fillvalue_hru, dim=c(nlon_hru,nlat_hru,1))
corr_array2 <- array(spatial_correlation$NA., dim=c(nlon_hru,nlat_hru))
dim(corr_array2)
# quick map correlation
cutpts <- seq(-1,1,length.out=9)
grid <- expand.grid(lon=lon_hru, lat=lat_hru)
#image(lon_hru,lat_hru,corr_array2, data=grid, at=cutpts, cuts=10,
pretty=T,
# col.regions=(rev(brewer.pal(10,"RdBu"))), main="Correlation (r)")
spei_1_corr_hru_cru<-levelplot(corr_array2 ~ lon * lat, data=grid,
at=cutpts, cuts=10, pretty=T,
col.regions= (rev(brewer.pal(10,"RdYlBu"))))
panel.text(325, 350, label="spei-1", font=1)
#(rev(brewer.pal(10,"RdYIBu")))
#main="spei-1 Correlation (pearson)"
#-----------------------------------Spatial Correlation-------------------
-------------------------
#depending on spei scale the length of time will change
temp_correlation <- data.frame(NA)
temp_data <- data.frame(hru="" ,cru="")
colnames(data) <- c("hru","cru")
for(i in seq(1,456,1)){
temp_data <- data.frame(hru = D_df02_hru[,i],cru = D_df02_cru[,i])
#data <- data.frame(hru = c(4,2,NA,4,7,8,-Inf,2,7,9,10),cru =
c(1,3,4,NA,3,6,1,3,5,8,-Inf))
#data2 <- data.frame(hru = c(NA,NA,NA,NA,NA,NA,NA,NA,NA,NA,NA),cru =
c(NA,NA,NA,NA,NA,NA,NA,NA,NA,NA,NA))
temp_data <- temp_data[is.finite(rowSums(temp_data)),]
if(nrow(temp_data) != 0){
r <- rcorr(temp_data[,1],temp_data[,2],type=c("pearson"))
if( r$P[2]<0.05){
temp_correlation[i,] <- r$r[2]
}
else{
temp_correlation[i,] <- NA
}
}
else{
temp_correlation[i,] <-NA
}
}
ts_corr <- ts(temp_correlation, start=c(1981,1),end = c(2018,12),frequency
= 12)
months<-data.frame(NA)
#months<-data.frame(c(seq(1,37,1)))
#months <-
data.frame(Jan=c(1:38),Feb=c(1:38),Mar=c(1:38),Apr=c(1:38),May=c(1:38),Jun=c(1:38),Jul=c(1:38),Aug=c(1
:38),Sep=c(1:38),Oct=c(1:38),Nov=c(1:38),Dec=c(1:38)
)#
colnames(months) <-
c("Jan","Feb","Mar","Apr","May","Jun","Jul","Aug","Sep","Oct","Nov","Dec")
for (c in (1:12)) {
f <- which(cycle(ts_corr) == c)
f <- f[!is.na(ts_corr[f])]
#numeric <- as.data.frame(sort(ts_corr[f]))
#numeric <- sort(ts_corr[f])
```

```
numeric <- ts_corr[f]
for(k in (1:length(numeric))){
months[k,c]<-numeric[k]
}
}
colnames(months) <-
c("Jan","Feb","Mar","Apr","May","Jun","Jul","Aug","Sep","Oct","Nov","Dec")
par(mar=c(2,4.1,1,1))
par(mfrow=c(4,2))
boxplot(months$Jan, months$Feb, months$Mar,
months$Apr,months$May,months$Jun,months$Jul,months$Aug,months$Sep,months$Oct,months$Nov,month
s$Dec
,
at = c(1,2,3,4,5,6,7,8,9,10,11,12),
names = c("Jan", "Feb", "Mar",
"Apr","May","Jun","Jul","Aug","Sep","Oct","Nov","Dec"),
col = "grey",
border = "black",
horizontal = FALSE,
notch = FALSE,
ylab = "R",
ylim = c(0.0,1.0)
)t
ext(0.0,"spei 01")
#main = "Multiple boxplots for comparision"
#col = c("pink","red")
area_mean_1 <- data.frame(NA)
for(i in seq(1,dim(D_df02_cru)[2],1)){
interim_data <- data.frame(hru = D_df02_hru[,i],cru = D_df02_cru[,i])
interim_data <- interim_data[is.finite(rowSums(interim_data)),]
area_mean_1[i,1] <- mean(interim_data$hru)
area_mean_1[i,2] <- mean(interim_data$cru)
}
colnames(area_mean_1) <- c("hru","cru")
#plot(area_mean_1$hru,type = 'l')
#plot(area_mean_1$cru,type = 'l')
```

ii. corr_analysis_spei3_sci

```
library(ncdf4)
library(Hmisc)
#-----------------------------------------Spatial and Temporal Correlation
Analysis-----------------------------------
#-----------------------------------------------------Spatial
Correlation-------------------------------------------
#collect data from resample HRU netcdf
# set path and filename
#ncpath <- "C:/Thesis2020/corr_analysis/"
ncpath <- "C:/Thesis2020/corr_analysis/corr_data/hru/"
#ncname <- "spei_3_hru_50km"
ncname <- "spei_3_hru_50km_green"
ncfname <- paste(ncpath, ncname, ".nc", sep="")
dname <- "spei" # note: precip means deficit (not precipitation)
# open a netCDF file
ncin <- nc_open(ncfname)
#print(ncin)
# get longitude and latitude
lon_hru <- ncvar_get(ncin,"lon")
```

```
nlon_hru <- dim(lon_hru)
head(lon_hru)
lat_hru <- ncvar_get(ncin,"lat")
nlat_hru <- dim(lat_hru)
head(lat_hru)
#print(c(nlon,nlat))
# get time
time_hru <- ncvar_get(ncin,"time")
#time_hru
tunits_hru <- ncatt_get(ncin,"time","units")
nt_hru <- dim(time_hru)
nt_hru
tunits_hru
# get spei from hru
spei_hru_array <- ncvar_get(ncin,dname)
splname_hru <- ncatt_get(ncin,dname,"long_name")
spunits_hru <- ncatt_get(ncin,dname,"units")
fillvalue_hru <- ncatt_get(ncin,dname,"_FillValue")
dim(spei_hru_array)
# get global attributes
#title <- ncatt_get(ncin,0,"title")
#institution <- ncatt_get(ncin,0,"institution")
#datasource <- ncatt_get(ncin,0,"source")
#references <- ncatt_get(ncin,0,"references")
#history <- ncatt_get(ncin,0,"history")
#Conventions <- ncatt_get(ncin,0,"Conventions")
nc_close(ncin)
# collect data from CRU netcdf
# set path and filename
#ncpath <- "C:/Thesis2020/corr_analysis/"
ncpath <- "C:/Thesis2020/corr_analysis/corr_data/cru/"
#ncname <- "spei_cru_3"
ncname <- "spei_cru_3_green"
ncfname <- paste(ncpath, ncname, ".nc", sep="")
dname <- "spei" # note: precip means deficit (not precipitation)
# open a netCDF file
ncin <- nc_open(ncfname)
#print(ncin)
# get longitude and latitude
lon_cru <- ncvar_get(ncin,"lon")
nlon_cru <- dim(lon_cru)
head(lon_cru)
lat_cru <- ncvar_get(ncin,"lat")
nlat_cru <- dim(lat_cru)
head(lat_cru)
#print(c(nlon,nlat))
# get time
time_cru <- ncvar_get(ncin,"time")
#time_cru
tunits_cru <- ncatt_get(ncin,"time","units")
nt_cru <- dim(time_cru)
nt_cru
tunits_cru
# get spei from hru
spei_cru_array <- ncvar_get(ncin,dname)
splname_cru <- ncatt_get(ncin,dname,"long_name")
spunits_cru <- ncatt_get(ncin,dname,"units")
fillvalue_cru <- ncatt_get(ncin,dname,"_FillValue")
```

```
dim(spei_cru_array)
# get global attributes
#title <- ncatt_get(ncin,0,"title")
#institution <- ncatt_get(ncin,0,"institution")
#datasource <- ncatt_get(ncin,0,"source")
#references <- ncatt_get(ncin,0,"references")
#history <- ncatt_get(ncin,0,"history")
#Conventions <- ncatt_get(ncin,0,"Conventions")
nc_close(ncin)
# load some packages
library(chron)
library(lattice)
library(RColorBrewer)
library(wesanderson)
# convert time -- split the time units string into fields for HRU and then
CRU
tustr_hru <- strsplit(tunits_hru$value, " ")
tdstr_hru <- strsplit(unlist(tustr_hru)[3], "-")
tmonth_hru <- as.integer(unlist(tdstr_hru)[2])
tday_hru <- as.integer(unlist(tdstr_hru)[3])
tyear_hru <- as.integer(unlist(tdstr_hru)[1])
#chron(time_hru,origin=c(tmonth_hru, tday_hru, tyear_hru))
tustr_cru <- strsplit(tunits_cru$value, " ")
tdstr_cru <- strsplit(unlist(tustr_cru)[3], "-")
tmonth_cru <- as.integer(unlist(tdstr_cru)[2])
tday_cru <- as.integer(unlist(tdstr_cru)[3])
tyear_cru <- as.integer(unlist(tdstr_cru)[1])
#chron(time_cru,origin=c(tmonth_cru, tday_cru, tyear_cru))
# replace netCDF fill values with NA's for both HRU and CRU.
spei_hru_array[spei_hru_array==fillvalue_hru$value] <- NA
spei_cru_array[spei_cru_array==fillvalue_cru$value] <- NA
#depending on the spei scale the starting time with available values might
change. So for spei_6 we dont have any values in first 5 layers or slices.
length(na.omit(as.vector(spei_hru_array[,,3])))
length(na.omit(as.vector(spei_cru_array[,,3])))
# get a single slice or layer (January)
m <- 6
spei_hru_slice <- spei_hru_array[,,m]
spei_cru_slice <- spei_cru_array[,,m]
# quick map
#image(lon_hru,lat_hru,spei_hru_slice, col=rev(brewer.pal(10,"RdBu")))
#image(lon_cru,lat_cru,spei_cru_slice, col=rev(brewer.pal(10,"RdBu")))
# reshape the arrays into vector
spei_hru_vec_long <- as.vector(spei_hru_array)
length(spei_hru_vec_long)
spei_cru_vec_long <- as.vector(spei_cru_array)
length(spei_cru_vec_long)
# reshape the vectors into a matrix
spei_hru_mat <- matrix(spei_hru_vec_long, nrow=nlon_hru*nlat_hru,
ncol=nt_hru)
dim(spei_hru_mat)
spei_cru_mat <- matrix(spei_cru_vec_long, nrow=nlon_cru*nlat_cru,
ncol=nt_cru)
dim(spei_cru_mat)
#head(na.omit(D_mat))
# create a dataframe
lonlat_hru <- as.matrix(expand.grid(lon_hru,lat_hru))
D_df02_hru <- data.frame(cbind(lonlat_hru,spei_hru_mat))
```

```
names(D_df02_hru) <- c("lon","lat")
#head(na.omit(D_df02_hru))
lonlat_cru <- as.matrix(expand.grid(lon_cru,lat_cru))
D_df02_cru <- data.frame(cbind(lonlat_cru,spei_cru_mat))
names(D_df02_cru) <- c("lon","lat")
#head(na.omit(D_df02_cru, 5))
# create a dataframe without missing values. This will be used later to
demonstrate how to convert a "short"
# data frame into full matrix or array for writing out as a netCDF file.
#D_df02 <- na.omit(D_df02_cru)
#head(D_df02,2)
# remove lon and lat columns and work on new data frame for both
# NOTE to Self: "FYI D_df03 is prepared after this step so just make sure
you dont get confusued."
Var1 <-D_df02_hru$lon
Var2 <-D_df02_hru$lat
drops <- c("lon","lat")
dim(D_df02_hru)
D_df02_hru <- D_df02_hru[ , !(names(D_df02_hru) %in% drops)]
dim(D_df02_hru)
#drop the first 2 layers due to time scale
D_df02_hru<-D_df02_hru[,3:456]
dim(D_df02_hru)
D_df03_hru <- as.data.frame(t(D_df02_hru))
D_df04_hru <- D_df03_hru
Var1 <-D_df02_cru$lon
Var2 <-D_df02_cru$lat
drops <- c("lon","lat")
dim(D_df02_cru)
D_df02_cru <- D_df02_cru[ , !(names(D_df02_cru) %in% drops)]
dim(D_df02_cru)
#drop the first 2 layers due to time scale
D_df02_cru<-D_df02_cru[,3:456]
dim(D_df02_cru)
D_df03_cru <- as.data.frame(t(D_df02_cru))
D_df04_cru <- D_df03_cru
#----------------------------------------Temporal Correlation-------------
-----------------------------
correlation <- data.frame(NA)
data <- data.frame(hru="" ,cru="")
colnames(data) <- c("hru","cru")
for(i in seq(1,3366,1)){
data <- data.frame(hru = D_df04_hru[,i],cru = D_df04_cru[,i])
#data <- data.frame(hru = c(4,2,NA,4,7,8,-Inf,2,7,9,10),cru =
c(1,3,4,NA,3,6,1,3,5,8,-Inf))
#data2 <- data.frame(hru = c(NA,NA,NA,NA,NA,NA,NA,NA,NA,NA,NA),cru =
c(NA,NA,NA,NA,NA,NA,NA,NA,NA,NA,NA))
data <- data[is.finite(rowSums(data)),]
if(nrow(data) != 0){
r <- rcorr(data[,1],data[,2],type=c("pearson"))
if( r$P[2]<0.05){
correlation[i,] <- r$r[2]
}
else{
correlation[i,] <- NA
}
}
else{
```

```
correlation[i,] <-NA
}
}
#Add longitude and latitude back to the correlation dataframe
#t_correlation <-t(correlation)
spatial_correlation <- data.frame(lon=Var1,lat=Var2,correlation)
#spei_df05 <-
fread("E:/Thesis_2020/Analysis/Preprocessing/Deficit_values/spei_netcdfspei12.csv"
)#
spei_df05 <- data.frame(spei_df05)
#spei_3_corr_hru_cru_raster<-Raster_it(spatial_correlation)
#writeRaster(spei_3_corr_hru_cru_raster,"spei_3_corr_hru_cru_raster.tif",format="GTiff"
)
#spei_df06[is.na(spei_df06)] <- 1e32
#corr_array <- array(fillvalue_hru, dim=c(nlon_hru,nlat_hru,1))
corr_array2 <- array(spatial_correlation$NA., dim=c(nlon_hru,nlat_hru))
dim(corr_array2)
# quick map correlation
cutpts <- seq(-1,1,length.out=9)
grid <- expand.grid(lon=lon_hru, lat=lat_hru)
#image(lon_hru,lat_hru,corr_array2, data=grid, at=cutpts, cuts=10,
pretty=T,
# col.regions=(rev(brewer.pal(10,"RdBu"))), main="Correlation (r)")
spei_3_corr_hru_cru<-levelplot(corr_array2 ~ lon * lat, data=grid,
at=cutpts, cuts=10, pretty=T,
col.regions=(rev(brewer.pal(10,"RdYlBu"))))
#-----------------------------------Temporal Correlation------------------
--------------------------
#depending on spei scale the length of time will change
temp_correlation <- data.frame(NA)
temp_data <- data.frame(hru="" ,cru="")
colnames(data) <- c("hru","cru")
for(i in seq(1,454,1)){
temp_data <- data.frame(hru = D_df02_hru[,i],cru = D_df02_cru[,i])
#data <- data.frame(hru = c(4,2,NA,4,7,8,-Inf,2,7,9,10),cru =
c(1,3,4,NA,3,6,1,3,5,8,-Inf))
#data2 <- data.frame(hru = c(NA,NA,NA,NA,NA,NA,NA,NA,NA,NA,NA),cru =
c(NA,NA,NA,NA,NA,NA,NA,NA,NA,NA,NA))
temp_data <- temp_data[is.finite(rowSums(temp_data)),]
if(nrow(temp_data) != 0){
r <- rcorr(temp_data[,1],temp_data[,2],type=c("pearson"))
if( r$P[2]<0.05){
temp_correlation[i,] <- r$r[2]
}
else{
temp_correlation[i,] <- NA
}
}
else{
temp_correlation[i,] <-NA
}
}
ts_corr <- ts(temp_correlation, start=c(1981,3),end = c(2018,12),frequency
= 12)
months<-data.frame(NA)
#months<-data.frame(c(seq(1,37,1)))
#months <-
```

```
data.frame(Jan=c(1:38),Feb=c(1:38),Mar=c(1:38),Apr=c(1:38),May=c(1:38),Jun=c(1:38),Jul=c(1:38),Aug=c(1
:38),Sep=c(1:38),Oct=c(1:38),Nov=c(1:38),Dec=c(1:38)
)#
colnames(months) <-
c("Jan","Feb","Mar","Apr","May","Jun","Jul","Aug","Sep","Oct","Nov","Dec")
for (c in (1:12)) {
f <- which(cycle(ts_corr) == c)
f <- f[!is.na(ts_corr[f])]
#numeric <- as.data.frame(sort(ts_corr[f]))
#numeric <- sort(ts_corr[f])
numeric <- ts_corr[f]
for(k in (1:length(numeric))){
months[k,c]<-numeric[k]
}
}
colnames(months) <-
c("Jan","Feb","Mar","Apr","May","Jun","Jul","Aug","Sep","Oct","Nov","Dec")
boxplot(months$Jan, months$Feb, months$Mar,
months$Apr,months$May,months$Jun,months$Jul,months$Aug,months$Sep,months$Oct,months$Nov,month
s$Dec
,
at = c(1,2,3,4,5,6,7,8,9,10,11,12),
names = c("Jan", "Feb", "Mar",
"Apr","May","Jun","Jul","Aug","Sep","Oct","Nov","Dec"),
col = "grey",
border = "black",
horizontal = FALSE,
notch = FALSE,
ylab = "R",
ylim = c(0.0,1.0)
)t
ext(0.0,"spei 03")
#main = "Multiple boxplots for comparision"
area_mean_3 <- data.frame(NA)
for(i in seq(1,dim(D_df02_cru)[2],1)){
interim_data <- data.frame(hru = D_df02_hru[,i],cru = D_df02_cru[,i])
interim_data <- interim_data[is.finite(rowSums(interim_data)),]
area_mean_3[i,1] <- mean(interim_data$hru)
area_mean_3[i,2] <- mean(interim_data$cru)
}
colnames(area_mean_3) <- c("hru","cru")
#plot(area_mean_3$hru,type = 'l')
#plot(area_mean_3$cru,type = 'l')
```

iii. corr_analysis_spei6_sci

```
library(ncdf4)
library(Hmisc)
#----------------------------------------Spatial and Temporal Correlation
Analysis----------------------------------
# HRU is short for High Resolution
# CRU indicates low resoltion data from Climate Research Unit database
#collect data from resample HRU netcdf
# set path and filename
#ncpath <- "C:/Thesis2020/corr_analysis/"
ncpath <- "C:/Thesis2020/corr_analysis/corr_data/hru/"
#ncname <- "spei_6_hru_50km"
ncname <- "spei_6_hru_50km_green"
```

```
ncfname <- paste(ncpath, ncname, ".nc", sep="")
dname <- "spei" # note: precip means deficit (not precipitation)
# open a netCDF file
ncin <- nc_open(ncfname)
#print(ncin)
# get longitude and latitude
lon_hru <- ncvar_get(ncin,"lon")
nlon_hru <- dim(lon_hru)
head(lon_hru)
lat_hru <- ncvar_get(ncin,"lat")
nlat_hru <- dim(lat_hru)
head(lat_hru)
#print(c(nlon,nlat))
# get time
time_hru <- ncvar_get(ncin,"time")
#time_hru
tunits_hru <- ncatt_get(ncin,"time","units")
nt_hru <- dim(time_hru)
nt_hru
tunits_hru
# get spei from hru
spei_hru_array <- ncvar_get(ncin,dname)
splname_hru <- ncatt_get(ncin,dname,"long_name")
spunits_hru <- ncatt_get(ncin,dname,"units")
fillvalue_hru <- ncatt_get(ncin,dname,"_FillValue")
dim(spei_hru_array)
# get global attributes
#title <- ncatt_get(ncin,0,"title")
#institution <- ncatt_get(ncin,0,"institution")
#datasource <- ncatt_get(ncin,0,"source")
#references <- ncatt_get(ncin,0,"references")
#history <- ncatt_get(ncin,0,"history")
#Conventions <- ncatt_get(ncin,0,"Conventions")
nc_close(ncin)
# collect data from CRU netcdf
# set path and filename
#ncpath <- "C:/Thesis2020/corr_analysis/"
ncpath <- "C:/Thesis2020/corr_analysis/corr_data/cru/"
#ncname <- "spei_cru_6"
ncname <- "spei_cru_6_green"
ncfname <- paste(ncpath, ncname, ".nc", sep="")
dname <- "spei" # note: precip means deficit (not precipitation)
# open a netCDF file
ncin <- nc_open(ncfname)
#print(ncin)
# get longitude and latitude
lon_cru <- ncvar_get(ncin,"lon")
nlon_cru <- dim(lon_cru)
head(lon_cru)
lat_cru <- ncvar_get(ncin,"lat")
nlat_cru <- dim(lat_cru)
head(lat_cru)
#print(c(nlon,nlat))
# get time
time_cru <- ncvar_get(ncin,"time")
#time_cru
tunits_cru <- ncatt_get(ncin,"time","units")
nt_cru <- dim(time_cru)
```

```
nt_cru
tunits_cru
# get spei from cru
spei_cru_array <- ncvar_get(ncin,dname)
splname_cru <- ncatt_get(ncin,dname,"long_name")
spunits_cru <- ncatt_get(ncin,dname,"units")
fillvalue_cru <- ncatt_get(ncin,dname,"_FillValue")
dim(spei_cru_array)
# get global attributes
#title <- ncatt_get(ncin,0,"title")
#institution <- ncatt_get(ncin,0,"institution")
#datasource <- ncatt_get(ncin,0,"source")
#references <- ncatt_get(ncin,0,"references")
#history <- ncatt_get(ncin,0,"history")
#Conventions <- ncatt_get(ncin,0,"Conventions")
nc_close(ncin)
# load some packages
library(chron)
library(lattice)
library(RColorBrewer)
library(wesanderson)
# replace netCDF fill values with NA's for both HRU and CRU.
spei_hru_array[spei_hru_array==fillvalue_hru$value] <- NA
spei_cru_array[spei_cru_array==fillvalue_cru$value] <- NA
#depending on the spei scale the starting time with available values might
change. So for spei_6 we dont have any values in first 5 layers or slices.
length(na.omit(as.vector(spei_hru_array[,,8])))
length(na.omit(as.vector(spei_cru_array[,,8])))
# get a single slice or layer (January)
m <- 6
spei_hru_slice <- spei_hru_array[,,m]
spei_cru_slice <- spei_cru_array[,,m]
# reshape the arrays into vector
spei_hru_vec_long <- as.vector(spei_hru_array)
length(spei_hru_vec_long)
spei_cru_vec_long <- as.vector(spei_cru_array)
length(spei_cru_vec_long)
# reshape the vectors into a matrix
spei_hru_mat <- matrix(spei_hru_vec_long, nrow=nlon_hru*nlat_hru,
ncol=nt_hru)
dim(spei_hru_mat)
spei_cru_mat <- matrix(spei_cru_vec_long, nrow=nlon_cru*nlat_cru,
ncol=nt_cru)
dim(spei_cru_mat)
#head(na.omit(D_mat))
# create a dataframe
lonlat_hru <- as.matrix(expand.grid(lon_hru,lat_hru))
D_df02_hru <- data.frame(cbind(lonlat_hru,spei_hru_mat))
names(D_df02_hru) <- c("lon","lat")
#head(na.omit(D_df02_hru))
lonlat_cru <- as.matrix(expand.grid(lon_cru,lat_cru))
D_df02_cru <- data.frame(cbind(lonlat_cru,spei_cru_mat))
names(D_df02_cru) <- c("lon","lat")
#head(na.omit(D_df02_cru, 2))
# remove lon and lat columns and work on new data frame for both
Var1 <-D_df02_hru$lon
Var2 <-D_df02_hru$lat
drops <- c("lon","lat")
```

```
dim(D_df02_hru)
D_df02_hru <- D_df02_hru[ , !(names(D_df02_hru) %in% drops)]
dim(D_df02_hru)
#drop the first 5 layers due to time scale
D_df02_hru<-D_df02_hru[,6:456]
dim(D_df02_hru)
D_df03_hru <- as.data.frame(t(D_df02_hru))
D_df04_hru <- D_df03_hru
Var1 <-D_df02_cru$lon
Var2 <-D_df02_cru$lat
drops <- c("lon","lat")
dim(D_df02_cru)
D_df02_cru <- D_df02_cru[ , !(names(D_df02_cru) %in% drops)]
dim(D_df02_cru)
#drop the first 5 layers due to time scale
D_df02_cru<-D_df02_cru[,6:456]
dim(D_df02_cru)
D_df03_cru <- as.data.frame(t(D_df02_cru))
D_df04_cru <- D_df03_cru
# --------------------------------------- Temporal correlation ------------
------------------------------
correlation <- data.frame(NA)
data <- data.frame(hru="" ,cru="")
colnames(data) <- c("hru","cru")
for(i in seq(1,3366,1)){
data <- data.frame(hru = D_df04_hru[,i],cru = D_df04_cru[,i])
#data <- data.frame(hru = c(4,2,NA,4,7,8,-Inf,2,7,9,10),cru =
c(1,3,4,NA,3,6,1,3,5,8,-Inf))
#data2 <- data.frame(hru = c(NA,NA,NA,NA,NA,NA,NA,NA,NA,NA,NA),cru =
c(NA,NA,NA,NA,NA,NA,NA,NA,NA,NA,NA))
data <- data[is.finite(rowSums(data)),]
if(nrow(data) != 0){
r <- rcorr(data[,1],data[,2],type=c("pearson"))
if( r$P[2] < 0.05){
correlation[i,] <- r$r[2]
}
else{
correlation[i,] <-NA
}
}
else{
correlation[i,] <-NA
}
}
#Add longitude and latitude back to the correlation dataframe
#t_correlation <-t(correlation)
spatial_correlation <- data.frame(lon=Var1,lat=Var2,correlation)
corr_array2 <- array(spatial_correlation$NA., dim=c(nlon_hru,nlat_hru))
dim(corr_array2)
# quick map correlation
cutpts <- seq(-1,1,length.out=9)
grid <- expand.grid(lon=lon_hru, lat=lat_hru)
spei_6_corr_hru_cru<-levelplot(corr_array2 ~ lon * lat, data=grid,
at=cutpts, cuts=10, pretty=T,
col.regions=(rev(brewer.pal(10,"RdYlBu"))))
#-----------------------------------spatial Correlation-------------------
-------------------------
#depending on spei scale the length of time will change
```

```
temp_correlation <- data.frame(NA)
temp_data <- data.frame(hru="" ,cru="")
colnames(data) <- c("hru","cru")
for(i in seq(1,451,1)){
temp_data <- data.frame(hru = D_df02_hru[,i],cru = D_df02_cru[,i])
#data <- data.frame(hru = c(4,2,NA,4,7,8,-Inf,2,7,9,10),cru =
c(1,3,4,NA,3,6,1,3,5,8,-Inf))
#data2 <- data.frame(hru = c(NA,NA,NA,NA,NA,NA,NA,NA,NA,NA,NA),cru =
c(NA,NA,NA,NA,NA,NA,NA,NA,NA,NA,NA))
temp_data <- temp_data[is.finite(rowSums(temp_data)),]
if(nrow(temp_data) != 0){
r <- rcorr(temp_data[,1],temp_data[,2],type=c("pearson"))
if( r$P[2]<0.05){
temp_correlation[i,] <- r$r[2]
}
else{
temp_correlation[i,] <-NA
}
}
else{
temp_correlation[i,] <-NA
}
}
ts_corr <- ts(temp_correlation, start=c(1981,6),end = c(2018,12),frequency
= 12)
months<-data.frame(NA)
for (c in (1:12)) {
f <- which(cycle(ts_corr) == c)
f <- f[!is.na(ts_corr[f])]
#numeric <- as.data.frame(sort(ts_corr[f]))
numeric<-(ts_corr[f])
#numeric <- sort(ts_corr[f])
for(k in (1:length(numeric))){
months[k,c]<-numeric[k]
}
}
colnames(months) <-
c("Jan","Feb","Mar","Apr","May","Jun","Jul","Aug","Sep","Oct","Nov","Dec")
boxplot(months$Jan, months$Feb, months$Mar,
months$Apr,months$May,months$Jun,months$Jul,months$Aug,months$Sep,months$Oct,months$Nov,month
s$Dec
,
at = c(1,2,3,4,5,6,7,8,9,10,11,12),
names = c("Jan", "Feb", "Mar",
"Apr","May","Jun","Jul","Aug","Sep","Oct","Nov","Dec"),
col = "grey",
border = "black",
horizontal = FALSE,
notch = FALSE,
ylab = "R",
ylim = c(0.0,1.0)
)t
ext(0.0,"spei 06")
#plot all the box plot one after another using par(mfrow=c(2,4)) and text()
to insert the name of the plot..
#dont forget to plot then in high res using png()
main = "Multiple boxplots for comparision"
area_mean_6 <- data.frame(NA)
```

```
for(i in seq(1,dim(D_df02_cru)[2],1)){
interim_data <- data.frame(hru = D_df02_hru[,i],cru = D_df02_cru[,i])
#removes all rows if there is an infinite value present in either cru or
hr data
interim_data <- interim_data[is.finite(rowSums(interim_data)),]
area_mean_6[i,1] <- mean(interim_data$hru)
area_mean_6[i,2] <- mean(interim_data$cru)
}
colnames(area_mean_6) <- c("hru","cru")
```

iv. corr_analysis_spei9_sci

```
library(ncdf4)
library(Hmisc)
#----------------------------------------Spatial and Temporal Correlation
Analysis----------------------------------
#-----------------------------------------------------Spatial
Correlation------------------------------------------
#collect data from resample HRU netcdf
# set path and filename
#ncpath <- "C:/Thesis2020/corr_analysis/"
ncpath <- "C:/Thesis2020/corr_analysis/corr_data/hru/"
#ncname <- "spei_9_hru_50km"
ncname <- "spei_9_hru_50km_green"
ncfname <- paste(ncpath, ncname, ".nc", sep="")
dname <- "spei" # note: precip means deficit (not precipitation)
# open a netCDF file
ncin <- nc_open(ncfname)
#print(ncin)
# get longitude and latitude
lon_hru <- ncvar_get(ncin,"lon")
nlon_hru <- dim(lon_hru)
head(lon_hru)
lat_hru <- ncvar_get(ncin,"lat")
nlat_hru <- dim(lat_hru)
head(lat_hru)
#print(c(nlon,nlat))
# get time
time_hru <- ncvar_get(ncin,"time")
#time_hru
tunits_hru <- ncatt_get(ncin,"time","units")
nt_hru <- dim(time_hru)
nt_hru
tunits_hru
# get spei from hru
spei_hru_array <- ncvar_get(ncin,dname)
splname_hru <- ncatt_get(ncin,dname,"long_name")
spunits_hru <- ncatt_get(ncin,dname,"units")
fillvalue_hru <- ncatt_get(ncin,dname,"_FillValue")
dim(spei_hru_array)
# get global attributes
#title <- ncatt_get(ncin,0,"title")
#institution <- ncatt_get(ncin,0,"institution")
#datasource <- ncatt_get(ncin,0,"source")
#references <- ncatt_get(ncin,0,"references")
#history <- ncatt_get(ncin,0,"history")
#Conventions <- ncatt_get(ncin,0,"Conventions")
nc_close(ncin)
```

```
# collect data from CRU netcdf
# set path and filename
#ncpath <- "C:/Thesis2020/corr_analysis/"
ncpath <- "C:/Thesis2020/corr_analysis/corr_data/cru/"
#ncname <- "spei_cru_9"
ncname <- "spei_cru_9_green"
ncfname <- paste(ncpath, ncname, ".nc", sep="")
dname <- "spei" # note: precip means deficit (not precipitation)
# open a netCDF file
ncin <- nc_open(ncfname)
#print(ncin)
# get longitude and latitude
lon_cru <- ncvar_get(ncin,"lon")
nlon_cru <- dim(lon_cru)
head(lon_cru)
lat_cru <- ncvar_get(ncin,"lat")
nlat_cru <- dim(lat_cru)
head(lat_cru)
#print(c(nlon,nlat))
# get time
time_cru <- ncvar_get(ncin,"time")
#time_cru
tunits_cru <- ncatt_get(ncin,"time","units")
nt_cru <- dim(time_cru)
nt_cru
tunits_cru
# get spei from hru
spei_cru_array <- ncvar_get(ncin,dname)
splname_cru <- ncatt_get(ncin,dname,"long_name")
spunits_cru <- ncatt_get(ncin,dname,"units")
fillvalue_cru <- ncatt_get(ncin,dname,"_FillValue")
dim(spei_cru_array)
# get global attributes
#title <- ncatt_get(ncin,0,"title")
#institution <- ncatt_get(ncin,0,"institution")
#datasource <- ncatt_get(ncin,0,"source")
#references <- ncatt_get(ncin,0,"references")
#history <- ncatt_get(ncin,0,"history")
#Conventions <- ncatt_get(ncin,0,"Conventions")
nc_close(ncin)
# load some packages
library(chron)
library(lattice)
library(RColorBrewer)
library(wesanderson)
# convert time -- split the time units string into fields for HRU and then
CRU
tustr_hru <- strsplit(tunits_hru$value, " ")
tdstr_hru <- strsplit(unlist(tustr_hru)[3], "-")
tmonth_hru <- as.integer(unlist(tdstr_hru)[2])
tday_hru <- as.integer(unlist(tdstr_hru)[3])
tyear_hru <- as.integer(unlist(tdstr_hru)[1])
#chron(time_hru,origin=c(tmonth_hru, tday_hru, tyear_hru))
tustr_cru <- strsplit(tunits_cru$value, " ")
tdstr_cru <- strsplit(unlist(tustr_cru)[3], "-")
tmonth_cru <- as.integer(unlist(tdstr_cru)[2])
tday_cru <- as.integer(unlist(tdstr_cru)[3])
tyear_cru <- as.integer(unlist(tdstr_cru)[1])
```

```
#chron(time_cru,origin=c(tmonth_cru, tday_cru, tyear_cru))
# replace netCDF fill values with NA's for both HRU and CRU.
spei_hru_array[spei_hru_array==fillvalue_hru$value] <- NA
spei_cru_array[spei_cru_array==fillvalue_cru$value] <- NA
#depending on the spei scale the starting time with available values might
change. So for spei_6 we dont have any values in first 5 layers or slices.
length(na.omit(as.vector(spei_hru_array[,,9])))
length(na.omit(as.vector(spei_cru_array[,,9])))
# get a single slice or layer (January)
m <- 9
spei_hru_slice <- spei_hru_array[,,m]
spei_cru_slice <- spei_cru_array[,,m]
# quick map
#image(lon_hru,lat_hru,spei_hru_slice, col=rev(brewer.pal(10,"RdBu")))
#image(lon_cru,lat_cru,spei_cru_slice, col=rev(brewer.pal(10,"RdBu")))
# reshape the arrays into vector
spei_hru_vec_long <- as.vector(spei_hru_array)
length(spei_hru_vec_long)
spei_cru_vec_long <- as.vector(spei_cru_array)
length(spei_cru_vec_long)
# reshape the vectors into a matrix
spei_hru_mat <- matrix(spei_hru_vec_long, nrow=nlon_hru*nlat_hru,
ncol=nt_hru)
dim(spei_hru_mat)
spei_cru_mat <- matrix(spei_cru_vec_long, nrow=nlon_cru*nlat_cru,
ncol=nt_cru)
dim(spei_cru_mat)
#head(na.omit(D_mat))
# create a dataframe
lonlat_hru <- as.matrix(expand.grid(lon_hru,lat_hru))
D_df02_hru <- data.frame(cbind(lonlat_hru,spei_hru_mat))
names(D_df02_hru) <- c("lon","lat")
#head(na.omit(D_df02_hru))
lonlat_cru <- as.matrix(expand.grid(lon_cru,lat_cru))
D_df02_cru <- data.frame(cbind(lonlat_cru,spei_cru_mat))
names(D_df02_cru) <- c("lon","lat")
#head(na.omit(D_df02_cru, 5))
# create a dataframe without missing values. This will be used later to
demonstrate how to convert a "short"
# data frame into full matrix or array for writing out as a netCDF file.
#D_df02 <- na.omit(D_df02_cru)
#head(D_df02,2)
# remove lon and lat columns and work on new data frame for both
# NOTE to Self: "FYI D_df03 is prepared after this step so just make sure
you dont get confusued."
Var1 <-D_df02_hru$lon
Var2 <-D_df02_hru$lat
drops <- c("lon","lat")
dim(D_df02_hru)
D_df02_hru <- D_df02_hru[ , !(names(D_df02_hru) %in% drops)]
dim(D_df02_hru)
#drop the first 8 layers due to time scale
D_df02_hru<-D_df02_hru[,9:456]
dim(D_df02_hru)
D_df03_hru <- as.data.frame(t(D_df02_hru))
D_df04_hru <- D_df03_hru
Var1 <-D_df02_cru$lon
Var2 <-D_df02_cru$lat
```

```
drops <- c("lon","lat")
dim(D_df02_cru)
D_df02_cru <- D_df02_cru[ , !(names(D_df02_cru) %in% drops)]
dim(D_df02_cru)
#drop the first 8 layers due to time scale
D_df02_cru<-D_df02_cru[,9:456]
dim(D_df02_cru)
D_df03_cru <- as.data.frame(t(D_df02_cru))
D_df04_cru <- D_df03_cru
#--------------------------------------------Temporal Correlation -------
-----------------------------
correlation <- data.frame(NA)
data <- data.frame(hru="" ,cru="")
colnames(data) <- c("hru","cru")
for(i in seq(1,3366,1)){
data <- data.frame(hru = D_df04_hru[,i],cru = D_df04_cru[,i])
data <- data[is.finite(rowSums(data)),]
if(nrow(data) != 0){
r <- rcorr(data[,1],data[,2],type=c("pearson"))
if( r$P[2]<0.05){
correlation[i,] <- r$r[2]
}
else{
correlation[i,] <- NA
}
}
else{
correlation[i,] <-NA
}
}
#Add longitude and latitude back to the correlation dataframe
#t_correlation <-t(correlation)
spatial_correlation <- data.frame(lon=Var1,lat=Var2,correlation)
#spei_df05 <-
fread("E:/Thesis_2020/Analysis/Preprocessing/Deficit_values/spei_netcdfspei12.csv"
)#
spei_df05 <- data.frame(spei_df05)
#spei_9_corr_hru_cru_raster<-Raster_it(spatial_correlation)
#writeRaster(spei_9_corr_hru_cru_raster,"spei_9_corr_hru_cru_raster.tif",format="GTiff"
)
#spei_df06[is.na(spei_df06)] <- 1e32
#corr_array <- array(fillvalue_hru, dim=c(nlon_hru,nlat_hru,1))
corr_array2 <- array(spatial_correlation$NA., dim=c(nlon_hru,nlat_hru))
dim(corr_array2)
# quick map correlation
cutpts <- seq(-1,1,length.out=9)
grid <- expand.grid(lon=lon_hru, lat=lat_hru)
#image(lon_hru,lat_hru,corr_array2, data=grid, at=cutpts, cuts=10,
pretty=T,
# col.regions=(rev(brewer.pal(10,"RdBu"))), main="Correlation (r)")
spei_9_corr_hru_cru <- levelplot(corr_array2 ~ lon * lat, data=grid,
at=cutpts, cuts=10, pretty=T,
col.regions=(rev(brewer.pal(10,"RdYlBu"))))
#----------------------------------Spatial Correlation------------------
-------------------------
#depending on spei scale the length of time will change
temp_correlation <- data.frame(NA)
temp_data <- data.frame(hru="" ,cru="")
```

```
colnames(data) <- c("hru","cru")
for(i in seq(1,448,1)){
temp_data <- data.frame(hru = D_df02_hru[,i],cru = D_df02_cru[,i])
temp_data <- temp_data[is.finite(rowSums(temp_data)),]
if(nrow(temp_data) != 0){
r <- rcorr(temp_data[,1],temp_data[,2],type=c("pearson"))
if( r$P[2]<0.05){
temp_correlation[i,] <- r$r[2]
}
else{
temp_correlation[i,] <- NA
}
}
else{
temp_correlation[i,] <-NA
}
}
ts_corr <- ts(temp_correlation, start=c(1981,9),end = c(2018,12),frequency
= 12)
months<-data.frame(NA)
#months<-data.frame(c(seq(1,37,1)))
#months <-
data.frame(Jan=c(1:38),Feb=c(1:38),Mar=c(1:38),Apr=c(1:38),May=c(1:38),Jun=c(1:38),Jul=c(1:38),Aug=c(1
:38),Sep=c(1:38),Oct=c(1:38),Nov=c(1:38),Dec=c(1:38)
)#
colnames(months) <-
c("Jan","Feb","Mar","Apr","May","Jun","Jul","Aug","Sep","Oct","Nov","Dec")
for (c in (1:12)) {
f <- which(cycle(ts_corr) == c)
f <- f[!is.na(ts_corr[f])]
#numeric <- as.data.frame(sort(ts_corr[f]))
#numeric <- sort(ts_corr[f])
numeric <- ts_corr[f]
for(k in (1:length(numeric))){
months[k,c]<-numeric[k]
}
}
colnames(months) <-
c("Jan","Feb","Mar","Apr","May","Jun","Jul","Aug","Sep","Oct","Nov","Dec")
boxplot(months$Jan, months$Feb, months$Mar,
months$Apr,months$May,months$Jun,months$Jul,months$Aug,months$Sep,months$Oct,months$Nov,month
s$Dec
,
at = c(1,2,3,4,5,6,7,8,9,10,11,12),
names = c("Jan", "Feb", "Mar",
"Apr","May","Jun","Jul","Aug","Sep","Oct","Nov","Dec"),
col = "grey",
border = "black",
horizontal = FALSE,
notch = FALSE,
ylab = "R",
ylim = c(0.0,1.0)
)t
ext(0.0,"spei 09")
area_mean_9 <- data.frame(NA)
for(i in seq(1,dim(D_df02_cru)[2],1)){
interim_data <- data.frame(hru = D_df02_hru[,i],cru = D_df02_cru[,i])
interim_data <- interim_data[is.finite(rowSums(interim_data)),]
```

```
area_mean_9[i,1] <- mean(interim_data$hru)
area_mean_9[i,2] <- mean(interim_data$cru)
}
colnames(area_mean_9) <- c("hru","cru")
```

v. corr_analysis_spei12_sci

```
library(ncdf4)
library(Hmisc)
#----------------------------------------Spatial and Temporal Correlation
Analysis----------------------------------
#-----------------------------------------------------Spatial
Correlation------------------------------------------
#collect data from resample HRU netcdf
# set path and filename
#ncpath <- "C:/Thesis2020/corr_analysis/"
ncpath <- "C:/Thesis2020/corr_analysis/corr_data/hru/"
#ncname <- "spei_12_hru_50km"
ncname <- "spei_12_hru_50km_green"
ncfname <- paste(ncpath, ncname, ".nc", sep="")
dname <- "spei" # note: precip means deficit (not precipitation)
# open a netCDF file
ncin <- nc_open(ncfname)
#print(ncin)
# get longitude and latitude
lon_hru <- ncvar_get(ncin,"lon")
nlon_hru <- dim(lon_hru)
head(lon_hru)
lat_hru <- ncvar_get(ncin,"lat")
nlat_hru <- dim(lat_hru)
head(lat_hru)
#print(c(nlon,nlat))
# get time
time_hru <- ncvar_get(ncin,"time")
#time_hru
tunits_hru <- ncatt_get(ncin,"time","units")
nt_hru <- dim(time_hru)
nt_hru
tunits_hru
# get spei from hru
spei_hru_array <- ncvar_get(ncin,dname)
splname_hru <- ncatt_get(ncin,dname,"long_name")
spunits_hru <- ncatt_get(ncin,dname,"units")
fillvalue_hru <- ncatt_get(ncin,dname,"_FillValue")
dim(spei_hru_array)
# get global attributes
#title <- ncatt_get(ncin,0,"title")
#institution <- ncatt_get(ncin,0,"institution")
#datasource <- ncatt_get(ncin,0,"source")
#references <- ncatt_get(ncin,0,"references")
#history <- ncatt_get(ncin,0,"history")
#Conventions <- ncatt_get(ncin,0,"Conventions")
nc_close(ncin)
# collect data from CRU netcdf
# set path and filename
#ncpath <- "C:/Thesis2020/corr_analysis/"
ncpath <- "C:/Thesis2020/corr_analysis/corr_data/cru/"
#ncname <- "spei_cru_12"
```

```
ncname <- "spei_cru_12_green"
ncfname <- paste(ncpath, ncname, ".nc", sep="")
dname <- "spei" # note: precip means deficit (not precipitation)
# open a netCDF file
ncin <- nc_open(ncfname)
#print(ncin)
# get longitude and latitude
lon_cru <- ncvar_get(ncin,"lon")
nlon_cru <- dim(lon_cru)
head(lon_cru)
lat_cru <- ncvar_get(ncin,"lat")
nlat_cru <- dim(lat_cru)
head(lat_cru)
#print(c(nlon,nlat))
# get time
time_cru <- ncvar_get(ncin,"time")
#time_cru
tunits_cru <- ncatt_get(ncin,"time","units")
nt_cru <- dim(time_cru)
nt_cru
tunits_cru
# get spei from hru
spei_cru_array <- ncvar_get(ncin,dname)
splname_cru <- ncatt_get(ncin,dname,"long_name")
spunits_cru <- ncatt_get(ncin,dname,"units")
fillvalue_cru <- ncatt_get(ncin,dname,"_FillValue")
dim(spei_cru_array)
# get global attributes
#title <- ncatt_get(ncin,0,"title")
#institution <- ncatt_get(ncin,0,"institution")
#datasource <- ncatt_get(ncin,0,"source")
#references <- ncatt_get(ncin,0,"references")
#history <- ncatt_get(ncin,0,"history")
#Conventions <- ncatt_get(ncin,0,"Conventions")
nc_close(ncin)
# load some packages
library(chron)
library(lattice)
library(RColorBrewer)
# convert time -- split the time units string into fields for HRU and then
CRU
tustr_hru <- strsplit(tunits_hru$value, " ")
tdstr_hru <- strsplit(unlist(tustr_hru)[3], "-")
tmonth_hru <- as.integer(unlist(tdstr_hru)[2])
tday_hru <- as.integer(unlist(tdstr_hru)[3])
tyear_hru <- as.integer(unlist(tdstr_hru)[1])
#chron(time_hru,origin=c(tmonth_hru, tday_hru, tyear_hru))
tustr_cru <- strsplit(tunits_cru$value, " ")
tdstr_cru <- strsplit(unlist(tustr_cru)[3], "-")
tmonth_cru <- as.integer(unlist(tdstr_cru)[2])
tday_cru <- as.integer(unlist(tdstr_cru)[3])
tyear_cru <- as.integer(unlist(tdstr_cru)[1])
#chron(time_cru,origin=c(tmonth_cru, tday_cru, tyear_cru))
# replace netCDF fill values with NA's for both HRU and CRU.
spei_hru_array[spei_hru_array==fillvalue_hru$value] <- NA
spei_cru_array[spei_cru_array==fillvalue_cru$value] <- NA
#depending on the spei scale the starting time with available values might
change. So for spei_6 we dont have any values in first 5 layers or slices.
```

```
length(na.omit(as.vector(spei_hru_array[,,12])))
length(na.omit(as.vector(spei_cru_array[,,12])))
# get a single slice or layer (January)
m <- 12
spei_hru_slice <- spei_hru_array[,,m]
spei_cru_slice <- spei_cru_array[,,m]
# quick map
#image(lon_hru,lat_hru,spei_hru_slice, col=rev(brewer.pal(10,"RdBu")))
#image(lon_cru,lat_cru,spei_cru_slice, col=rev(brewer.pal(10,"RdBu")))
# reshape the arrays into vector
spei_hru_vec_long <- as.vector(spei_hru_array)
length(spei_hru_vec_long)
spei_cru_vec_long <- as.vector(spei_cru_array)
length(spei_cru_vec_long)
# reshape the vectors into a matrix
spei_hru_mat <- matrix(spei_hru_vec_long, nrow=nlon_hru*nlat_hru,
ncol=nt_hru)
dim(spei_hru_mat)
spei_cru_mat <- matrix(spei_cru_vec_long, nrow=nlon_cru*nlat_cru,
ncol=nt_cru)
dim(spei_cru_mat)
#head(na.omit(D_mat))
# create a dataframe
lonlat_hru <- as.matrix(expand.grid(lon_hru,lat_hru))
D_df02_hru <- data.frame(cbind(lonlat_hru,spei_hru_mat))
names(D_df02_hru) <- c("lon","lat")
#head(na.omit(D_df02_hru))
lonlat_cru <- as.matrix(expand.grid(lon_cru,lat_cru))
D_df02_cru <- data.frame(cbind(lonlat_cru,spei_cru_mat))
names(D_df02_cru) <- c("lon","lat")
#head(na.omit(D_df02_cru, 5))
# create a dataframe without missing values. This will be used later to
demonstrate how to convert a "short"
# data frame into full matrix or array for writing out as a netCDF file.
#D_df02 <- na.omit(D_df02_cru)
#head(D_df02,2)
# remove lon and lat columns and work on new data frame for both
# NOTE to Self: "FYI D_df03 is prepared after this step so just make sure
you dont get confusued."
Var1 <-D_df02_hru$lon
Var2 <-D_df02_hru$lat
drops <- c("lon","lat")
dim(D_df02_hru)
D_df02_hru <- D_df02_hru[ , !(names(D_df02_hru) %in% drops)]
dim(D_df02_hru)
#drop the first 11 layers due to time scale
D_df02_hru<-D_df02_hru[,12:456]
dim(D_df02_hru)
D_df03_hru <- as.data.frame(t(D_df02_hru))
D_df04_hru <- D_df03_hru
Var1 <-D_df02_cru$lon
Var2 <-D_df02_cru$lat
drops <- c("lon","lat")
dim(D_df02_cru)
D_df02_cru <- D_df02_cru[ , !(names(D_df02_cru) %in% drops)]
dim(D_df02_cru)
#drop the first 11 layers due to time scale
D_df02_cru<-D_df02_cru[,12:456]
```

```
dim(D_df02_cru)
D_df03_cru <- as.data.frame(t(D_df02_cru))
D_df04_cru <- D_df03_cru
#-----------------------------------------Temporal Analysis---------------
---------------------
correlation <- data.frame(NA)
data <- data.frame(hru="" ,cru="")
colnames(data) <- c("hru","cru")
for(i in seq(1,3366,1)){
data <- data.frame(hru = D_df04_hru[,i],cru = D_df04_cru[,i])
data <- data[is.finite(rowSums(data)),]
if(nrow(data) != 0){
r <- rcorr(data[,1],data[,2],type=c("pearson"))
if( r$P[2]<0.05){
correlation[i,] <- r$r[2]
}
else{
correlation[i,] <- NA
}
}
else{
correlation[i,] <-NA
}
}
#Add longitude and latitude back to the correlation dataframe
#t_correlation <-t(correlation)
spatial_correlation <- data.frame(lon=Var1,lat=Var2,correlation)
#spei_df05 <-
fread("E:/Thesis_2020/Analysis/Preprocessing/Deficit_values/spei_netcdfspei12.csv"
)#
spei_df05 <- data.frame(spei_df05)
#spei_12_corr_hru_cru_raster<-Raster_it(spatial_correlation)
#writeRaster(spei_12_corr_hru_cru_raster,"spei_12_corr_hru_cru_raster.tif",format="GTiff"
)
#spei_df06[is.na(spei_df06)] <- 1e32
#corr_array <- array(fillvalue_hru, dim=c(nlon_hru,nlat_hru,1))
corr_array2 <- array(spatial_correlation$NA., dim=c(nlon_hru,nlat_hru))
dim(corr_array2)
# quick map correlation
cutpts <- seq(-1,1,length.out=9)
grid <- expand.grid(lon=lon_hru, lat=lat_hru)
#image(lon_hru,lat_hru,corr_array2, data=grid, at=cutpts, cuts=10,
pretty=T,
# col.regions=(rev(brewer.pal(10,"RdBu"))), main="Correlation (r)")
spei_12_corr_hru_cru<-levelplot(corr_array2 ~ lon * lat, data=grid,
at=cutpts, cuts=10, pretty=T,
col.regions=(rev(brewer.pal(10,"RdYlBu"))))
#-----------------------------------Spatial Correlation-------------------
-------------------------
#depending on spei scale the length of time will change
temp_correlation <- data.frame(NA)
temp_data <- data.frame(hru="" ,cru="")
colnames(data) <- c("hru","cru")
for(i in seq(1,dim(D_df02_cru)[2],1)){
temp_data <- data.frame(hru = D_df02_hru[,i],cru = D_df02_cru[,i])
temp_data <- temp_data[is.finite(rowSums(temp_data)),]
if(nrow(temp_data) != 0){
r <- rcorr(temp_data[,1],temp_data[,2],type=c("pearson"))
```

```
if( r$P[2]<0.05){
temp_correlation[i,] <- r$r[2]
}
else{
temp_correlation[i,] <- NA
}
}
else{
temp_correlation[i,] <-NA
}
}
ts_corr <- ts(temp_correlation, start=c(1981,12),end = c(2018,12),frequency
= 12)
months<-data.frame(NA)
#months<-data.frame(c(seq(1,37,1)))
#months <-
data.frame(Jan=c(1:38),Feb=c(1:38),Mar=c(1:38),Apr=c(1:38),May=c(1:38),Jun=c(1:38),Jul=c(1:38),Aug=c(1
:38),Sep=c(1:38),Oct=c(1:38),Nov=c(1:38),Dec=c(1:38)
)#
colnames(months) <-
c("Jan","Feb","Mar","Apr","May","Jun","Jul","Aug","Sep","Oct","Nov","Dec")
for (c in (1:12)) {
f <- which(cycle(ts_corr) == c)
f <- f[!is.na(ts_corr[f])]
#numeric <- as.data.frame(sort(ts_corr[f]))
#numeric <- sort(ts_corr[f])
numeric <- ts_corr[f]
for(k in (1:length(numeric))){
months[k,c]<-numeric[k]
}
}
colnames(months) <-
c("Jan","Feb","Mar","Apr","May","Jun","Jul","Aug","Sep","Oct","Nov","Dec")
boxplot(months$Jan, months$Feb, months$Mar,
months$Apr,months$May,months$Jun,months$Jul,months$Aug,months$Sep,months$Oct,months$Nov,month
s$Dec
,
at = c(1,2,3,4,5,6,7,8,9,10,11,12),
names = c("Jan", "Feb", "Mar",
"Apr","May","Jun","Jul","Aug","Sep","Oct","Nov","Dec"),
col = "grey",
border = "black",
horizontal = FALSE,
notch = FALSE,
ylab = "R",
ylim = c(0.0,1.0)
)t
ext(0.0,"spei 12")
area_mean_12 <- data.frame(NA)
for(i in seq(1,dim(D_df02_cru)[2],1)){
interim_data <- data.frame(hru = D_df02_hru[,i],cru = D_df02_cru[,i])
interim_data <- interim_data[is.finite(rowSums(interim_data)),]
area_mean_12[i,1] <- mean(interim_data$hru)
area_mean_12[i,2] <- mean(interim_data$cru)
}
colnames(area_mean_12) <- c("hru","cru")
```

vi. corr_analysis_spei24_sci

```
library(ncdf4)
library(Hmisc)
#----------------------------------------Spatial and Temporal Correlation
Analysis----------------------------------
#------------------------------------------------------Spatial
Correlation------------------------------------------
#collect data from resample HRU netcdf
# set path and filename
#ncpath <- "C:/Thesis2020/corr_analysis/"
ncpath <- "C:/Thesis2020/corr_analysis/corr_data/hru/"
#ncname <- "spei_24_hru_50km"
ncname <- "spei_24_hru_50km_green"
ncfname <- paste(ncpath, ncname, ".nc", sep="")
dname <- "spei" # note: precip means deficit (not precipitation)
# open a netCDF file
ncin <- nc_open(ncfname)
#print(ncin)
# get longitude and latitude
lon_hru <- ncvar_get(ncin,"lon")
nlon_hru <- dim(lon_hru)
head(lon_hru)
lat_hru <- ncvar_get(ncin,"lat")
nlat_hru <- dim(lat_hru)
head(lat_hru)
#print(c(nlon,nlat))
# get time
time_hru <- ncvar_get(ncin,"time")
#time_hru
tunits_hru <- ncatt_get(ncin,"time","units")
nt_hru <- dim(time_hru)
nt_hru
tunits_hru
# get spei from hru
spei_hru_array <- ncvar_get(ncin,dname)
splname_hru <- ncatt_get(ncin,dname,"long_name")
spunits_hru <- ncatt_get(ncin,dname,"units")
fillvalue_hru <- ncatt_get(ncin,dname,"_FillValue")
dim(spei_hru_array)
# get global attributes
#title <- ncatt_get(ncin,0,"title")
#institution <- ncatt_get(ncin,0,"institution")
#datasource <- ncatt_get(ncin,0,"source")
#references <- ncatt_get(ncin,0,"references")
#history <- ncatt_get(ncin,0,"history")
#Conventions <- ncatt_get(ncin,0,"Conventions")
nc_close(ncin)
# collect data from CRU netcdf
# set path and filename
#ncpath <- "C:/Thesis2020/corr_analysis/"
ncpath <- "C:/Thesis2020/corr_analysis/corr_data/cru/"
#ncname <- "spei_cru_24"
ncname <- "spei_cru_24_green"
ncfname <- paste(ncpath, ncname, ".nc", sep="")
dname <- "spei" # note: precip means deficit (not precipitation)
# open a netCDF file
```

```
ncin <- nc_open(ncfname)
#print(ncin)
# get longitude and latitude
lon_cru <- ncvar_get(ncin,"lon")
nlon_cru <- dim(lon_cru)
head(lon_cru)
lat_cru <- ncvar_get(ncin,"lat")
nlat_cru <- dim(lat_cru)
head(lat_cru)
#print(c(nlon,nlat))
# get time
time_cru <- ncvar_get(ncin,"time")
#time_cru
tunits_cru <- ncatt_get(ncin,"time","units")
nt_cru <- dim(time_cru)
nt_cru
tunits_cru
# get spei from hru
spei_cru_array <- ncvar_get(ncin,dname)
splname_cru <- ncatt_get(ncin,dname,"long_name")
spunits_cru <- ncatt_get(ncin,dname,"units")
fillvalue_cru <- ncatt_get(ncin,dname,"_FillValue")
dim(spei_cru_array)
# get global attributes
#title <- ncatt_get(ncin,0,"title")
#institution <- ncatt_get(ncin,0,"institution")
#datasource <- ncatt_get(ncin,0,"source")
#references <- ncatt_get(ncin,0,"references")
#history <- ncatt_get(ncin,0,"history")
#Conventions <- ncatt_get(ncin,0,"Conventions")
nc_close(ncin)
# load some packages
library(chron)
library(lattice)
library(RColorBrewer)
# convert time -- split the time units string into fields for HRU and then
CRU
tustr_hru <- strsplit(tunits_hru$value, " ")
tdstr_hru <- strsplit(unlist(tustr_hru)[3], "-")
tmonth_hru <- as.integer(unlist(tdstr_hru)[2])
tday_hru <- as.integer(unlist(tdstr_hru)[3])
tyear_hru <- as.integer(unlist(tdstr_hru)[1])
#chron(time_hru,origin=c(tmonth_hru, tday_hru, tyear_hru))
tustr_cru <- strsplit(tunits_cru$value, " ")
tdstr_cru <- strsplit(unlist(tustr_cru)[3], "-")
tmonth_cru <- as.integer(unlist(tdstr_cru)[2])
tday_cru <- as.integer(unlist(tdstr_cru)[3])
tyear_cru <- as.integer(unlist(tdstr_cru)[1])
#chron(time_cru,origin=c(tmonth_cru, tday_cru, tyear_cru))
# replace netCDF fill values with NA's for both HRU and CRU.
spei_hru_array[spei_hru_array==fillvalue_hru$value] <- NA
spei_cru_array[spei_cru_array==fillvalue_cru$value] <- NA
#depending on the spei scale the starting time with available values might
change. So for spei_6 we dont have any values in first 5 layers or slices.
length(na.omit(as.vector(spei_hru_array[,,12])))
length(na.omit(as.vector(spei_cru_array[,,12])))
# get a single slice or layer (January)
m <- 24
```

```
spei_hru_slice <- spei_hru_array[,,m]
spei_cru_slice <- spei_cru_array[,,m]
# quick map
#image(lon_hru,lat_hru,spei_hru_slice, col=rev(brewer.pal(10,"RdBu")))
#image(lon_cru,lat_cru,spei_cru_slice, col=rev(brewer.pal(10,"RdBu")))
# reshape the arrays into vector
spei_hru_vec_long <- as.vector(spei_hru_array)
length(spei_hru_vec_long)
spei_cru_vec_long <- as.vector(spei_cru_array)
length(spei_cru_vec_long)
# reshape the vectors into a matrix
spei_hru_mat <- matrix(spei_hru_vec_long, nrow=nlon_hru*nlat_hru,
ncol=nt_hru)
dim(spei_hru_mat)
spei_cru_mat <- matrix(spei_cru_vec_long, nrow=nlon_cru*nlat_cru,
ncol=nt_cru)
dim(spei_cru_mat)
#head(na.omit(D_mat))
# create a dataframe
lonlat_hru <- as.matrix(expand.grid(lon_hru,lat_hru))
D_df02_hru <- data.frame(cbind(lonlat_hru,spei_hru_mat))
names(D_df02_hru) <- c("lon","lat")
#head(na.omit(D_df02_hru))
lonlat_cru <- as.matrix(expand.grid(lon_cru,lat_cru))
D_df02_cru <- data.frame(cbind(lonlat_cru,spei_cru_mat))
names(D_df02_cru) <- c("lon","lat")
#head(na.omit(D_df02_cru, 5))
# create a dataframe without missing values. This will be used later to
demonstrate how to convert a "short"
# data frame into full matrix or array for writing out as a netCDF file.
#D_df02 <- na.omit(D_df02_cru)
#head(D_df02,2)
# remove lon and lat columns and work on new data frame for both
# NOTE to Self: "FYI D_df03 is prepared after this step so just make sure
you dont get confusued."
Var1 <-D_df02_hru$lon
Var2 <-D_df02_hru$lat
drops <- c("lon","lat")
dim(D_df02_hru)
D_df02_hru <- D_df02_hru[ , !(names(D_df02_hru) %in% drops)]
dim(D_df02_hru)
#drop the first 11 layers due to time scale
D_df02_hru<-D_df02_hru[,24:456]
dim(D_df02_hru)
D_df03_hru <- as.data.frame(t(D_df02_hru))
D_df04_hru <- D_df03_hru
Var1 <-D_df02_cru$lon
Var2 <-D_df02_cru$lat
drops <- c("lon","lat")
dim(D_df02_cru)
D_df02_cru <- D_df02_cru[ , !(names(D_df02_cru) %in% drops)]
dim(D_df02_cru)
#drop the first 11 layers due to time scale
D_df02_cru<-D_df02_cru[,24:456]
dim(D_df02_cru)
D_df03_cru <- as.data.frame(t(D_df02_cru))
D_df04_cru <- D_df03_cru
#---------------------------------------------------Temporal Correlation--
```

```
-----------------------------
correlation <- data.frame(NA)
data <- data.frame(hru="" ,cru="")
colnames(data) <- c("hru","cru")
for(i in seq(1,3366,1)){
data <- data.frame(hru = D_df04_hru[,i],cru = D_df04_cru[,i])
data <- data[is.finite(rowSums(data)),]
if(nrow(data) != 0){
r <- rcorr(data[,1],data[,2],type=c("pearson"))
if( r$P[2]<0.05){
correlation[i,] <- r$r[2]
}
else{
correlation[i,] <- NA
}
}
else{
correlation[i,] <-NA
}
}
#Add longitude and latitude back to the correlation dataframe
#t_correlation <-t(correlation)
spatial_correlation <- data.frame(lon=Var1,lat=Var2,correlation)
#spei_df05 <-
fread("E:/Thesis_2020/Analysis/Preprocessing/Deficit_values/spei_netcdfspei12.csv"
)#
spei_df05 <- data.frame(spei_df05)
#spei_24_corr_hru_cru_raster<-Raster_it(spatial_correlation)
#writeRaster(spei_24_corr_hru_cru_raster,"spei_24_corr_hru_cru_raster.tif",format="GTiff"
)
#spei_df06[is.na(spei_df06)] <- 1e32
#corr_array <- array(fillvalue_hru, dim=c(nlon_hru,nlat_hru,1))
corr_array2 <- array(spatial_correlation$NA., dim=c(nlon_hru,nlat_hru))
dim(corr_array2)
# quick map correlation
cutpts <- seq(-1,1,length.out=9)
grid <- expand.grid(lon=lon_hru, lat=lat_hru)
#image(lon_hru,lat_hru,corr_array2, data=grid, at=cutpts, cuts=10,
pretty=T,
# col.regions=(rev(brewer.pal(10,"RdBu"))), main="Correlation (r)")
spei_24_corr_hru_cru<-levelplot(corr_array2 ~ lon * lat, data=grid,
at=cutpts, cuts=10, pretty=T,
col.regions=(rev(brewer.pal(10,"RdYlBu"))))
#-----------------------------------Temporal Correlation------------------
---------------------------
#depending on spei scale the length of time will change
temp_correlation <- data.frame(NA)
temp_data <- data.frame(hru="" ,cru="")
colnames(data) <- c("hru","cru")
dim(D_df02_cru)[2]
for(i in seq(1,dim(D_df02_cru)[2],1)){
temp_data <- data.frame(hru = D_df02_hru[,i],cru = D_df02_cru[,i])
#data <- data.frame(hru = c(4,2,NA,4,7,8,-Inf,2,7,9,10),cru =
c(1,3,4,NA,3,6,1,3,5,8,-Inf))
#data2 <- data.frame(hru = c(NA,NA,NA,NA,NA,NA,NA,NA,NA,NA,NA),cru =
c(NA,NA,NA,NA,NA,NA,NA,NA,NA,NA,NA))
temp_data <- temp_data[is.finite(rowSums(temp_data)),]
if(nrow(temp_data) != 0){
```

```
r <- rcorr(temp_data[,1],temp_data[,2],type=c("pearson"))
if( r$P[2]<0.05){
temp_correlation[i,] <- r$r[2]
}
else{
temp_correlation[i,] <- NA
}
}
else{
temp_correlation[i,] <-NA
}
}
ts_corr <- ts(temp_correlation, start=c(1982,12),end = c(2018,12),frequency
= 12)
months<-data.frame(NA)
#months<-data.frame(c(seq(1,37,1)))
#months <-
data.frame(Jan=c(1:38),Feb=c(1:38),Mar=c(1:38),Apr=c(1:38),May=c(1:38),Jun=c(1:38),Jul=c(1:38),Aug=c(1
:38),Sep=c(1:38),Oct=c(1:38),Nov=c(1:38),Dec=c(1:38)
)#
colnames(months) <-
c("Jan","Feb","Mar","Apr","May","Jun","Jul","Aug","Sep","Oct","Nov","Dec")
for (c in (1:12)) {
f <- which(cycle(ts_corr) == c)
f <- f[!is.na(ts_corr[f])]
#numeric <- as.data.frame(sort(ts_corr[f]))
#numeric <- sort(ts_corr[f])
numeric <- ts_corr[f]
for(k in (1:length(numeric))){
months[k,c]<-numeric[k]
}
}
colnames(months) <-
c("Jan","Feb","Mar","Apr","May","Jun","Jul","Aug","Sep","Oct","Nov","Dec")
boxplot(months$Jan, months$Feb, months$Mar,
months$Apr,months$May,months$Jun,months$Jul,months$Aug,months$Sep,months$Oct,months$Nov,month
s$Dec
,
at = c(1,2,3,4,5,6,7,8,9,10,11,12),
names = c("Jan", "Feb", "Mar",
"Apr","May","Jun","Jul","Aug","Sep","Oct","Nov","Dec"),
col = "grey",
border = "black",
horizontal = FALSE,
notch = FALSE,
ylab = "R",
ylim = c(0.0,1.0)
)t
ext(0.0,"spei 24")
area_mean_24 <- data.frame(NA)
for(i in seq(1,dim(D_df02_cru)[2],1)){
interim_data <- data.frame(hru = D_df02_hru[,i],cru = D_df02_cru[,i])
interim_data <- interim_data[is.finite(rowSums(interim_data)),]
area_mean_24[i,1] <- mean(interim_data$hru)
area_mean_24[i,2] <- mean(interim_data$cru)
}
colnames(area_mean_24) <- c("hru","cru")
```

vii. corr_analysis_spei36_sci

```
library(ncdf4)
library(Hmisc)
#----------------------------------------Spatial and Temporal Correlation
Analysis----------------------------------
#------------------------------------------------------Spatial
Correlation------------------------------------------
#collect data from resample HRU netcdf
# set path and filename
#ncpath <- "C:/Thesis2020/corr_analysis/"
ncpath <- "C:/Thesis2020/corr_analysis/corr_data/hru/"
#ncname <- "spei_36_hru_50km"
ncname <- "spei_36_hru_50km_green"
ncfname <- paste(ncpath, ncname, ".nc", sep="")
dname <- "spei" # note: precip means deficit (not precipitation)
# open a netCDF file
ncin <- nc_open(ncfname)
#print(ncin)
# get longitude and latitude
lon_hru <- ncvar_get(ncin,"lon")
nlon_hru <- dim(lon_hru)
head(lon_hru)
lat_hru <- ncvar_get(ncin,"lat")
nlat_hru <- dim(lat_hru)
head(lat_hru)
#print(c(nlon,nlat))
# get time
time_hru <- ncvar_get(ncin,"time")
#time_hru
tunits_hru <- ncatt_get(ncin,"time","units")
nt_hru <- dim(time_hru)
nt_hru
tunits_hru
# get spei from hru
spei_hru_array <- ncvar_get(ncin,dname)
splname_hru <- ncatt_get(ncin,dname,"long_name")
spunits_hru <- ncatt_get(ncin,dname,"units")
fillvalue_hru <- ncatt_get(ncin,dname,"_FillValue")
dim(spei_hru_array)
# get global attributes
#title <- ncatt_get(ncin,0,"title")
#institution <- ncatt_get(ncin,0,"institution")
#datasource <- ncatt_get(ncin,0,"source")
#references <- ncatt_get(ncin,0,"references")
#history <- ncatt_get(ncin,0,"history")
#Conventions <- ncatt_get(ncin,0,"Conventions")
nc_close(ncin)
# collect data from CRU netcdf
# set path and filename
#ncpath <- "C:/Thesis2020/corr_analysis/"
ncpath <- "C:/Thesis2020/corr_analysis/corr_data/cru/"
#ncname <- "spei_cru_36"
ncname <- "spei_cru_36_green"
ncfname <- paste(ncpath, ncname, ".nc", sep="")
dname <- "spei" # note: precip means deficit (not precipitation)
# open a netCDF file
```

```
ncin <- nc_open(ncfname)
#print(ncin)
# get longitude and latitude
lon_cru <- ncvar_get(ncin,"lon")
nlon_cru <- dim(lon_cru)
head(lon_cru)
lat_cru <- ncvar_get(ncin,"lat")
nlat_cru <- dim(lat_cru)
head(lat_cru)
#print(c(nlon,nlat))
# get time
time_cru <- ncvar_get(ncin,"time")
#time_cru
tunits_cru <- ncatt_get(ncin,"time","units")
nt_cru <- dim(time_cru)
nt_cru
tunits_cru
# get spei from hru
spei_cru_array <- ncvar_get(ncin,dname)
splname_cru <- ncatt_get(ncin,dname,"long_name")
spunits_cru <- ncatt_get(ncin,dname,"units")
fillvalue_cru <- ncatt_get(ncin,dname,"_FillValue")
dim(spei_cru_array)
# get global attributes
#title <- ncatt_get(ncin,0,"title")
#institution <- ncatt_get(ncin,0,"institution")
#datasource <- ncatt_get(ncin,0,"source")
#references <- ncatt_get(ncin,0,"references")
#history <- ncatt_get(ncin,0,"history")
#Conventions <- ncatt_get(ncin,0,"Conventions")
nc_close(ncin)
# load some packages
library(chron)
library(lattice)
library(RColorBrewer)
# convert time -- split the time units string into fields for HRU and then
CRU
tustr_hru <- strsplit(tunits_hru$value, " ")
tdstr_hru <- strsplit(unlist(tustr_hru)[3], "-")
tmonth_hru <- as.integer(unlist(tdstr_hru)[2])
tday_hru <- as.integer(unlist(tdstr_hru)[3])
tyear_hru <- as.integer(unlist(tdstr_hru)[1])
#chron(time_hru,origin=c(tmonth_hru, tday_hru, tyear_hru))
tustr_cru <- strsplit(tunits_cru$value, " ")
tdstr_cru <- strsplit(unlist(tustr_cru)[3], "-")
tmonth_cru <- as.integer(unlist(tdstr_cru)[2])
tday_cru <- as.integer(unlist(tdstr_cru)[3])
tyear_cru <- as.integer(unlist(tdstr_cru)[1])
#chron(time_cru,origin=c(tmonth_cru, tday_cru, tyear_cru))
# replace netCDF fill values with NA's for both HRU and CRU.
spei_hru_array[spei_hru_array==fillvalue_hru$value] <- NA
spei_cru_array[spei_cru_array==fillvalue_cru$value] <- NA
#depending on the spei scale the starting time with available values might
change. So for spei_6 we dont have any values in first 5 layers or slices.
length(na.omit(as.vector(spei_hru_array[,,36])))
length(na.omit(as.vector(spei_cru_array[,,36])))
# get a single slice or layer (January)
m <- 36
```

```
spei_hru_slice <- spei_hru_array[,,m]
spei_cru_slice <- spei_cru_array[,,m]
# quick map
#image(lon_hru,lat_hru,spei_hru_slice, col=rev(brewer.pal(10,"RdBu")))
#image(lon_cru,lat_cru,spei_cru_slice, col=rev(brewer.pal(10,"RdBu")))
# reshape the arrays into vector
spei_hru_vec_long <- as.vector(spei_hru_array)
length(spei_hru_vec_long)
spei_cru_vec_long <- as.vector(spei_cru_array)
length(spei_cru_vec_long)
# reshape the vectors into a matrix
spei_hru_mat <- matrix(spei_hru_vec_long, nrow=nlon_hru*nlat_hru,
ncol=nt_hru)
dim(spei_hru_mat)
spei_cru_mat <- matrix(spei_cru_vec_long, nrow=nlon_cru*nlat_cru,
ncol=nt_cru)
dim(spei_cru_mat)
#head(na.omit(D_mat))
# create a dataframe
lonlat_hru <- as.matrix(expand.grid(lon_hru,lat_hru))
D_df02_hru <- data.frame(cbind(lonlat_hru,spei_hru_mat))
names(D_df02_hru) <- c("lon","lat")
#head(na.omit(D_df02_hru))
lonlat_cru <- as.matrix(expand.grid(lon_cru,lat_cru))
D_df02_cru <- data.frame(cbind(lonlat_cru,spei_cru_mat))
names(D_df02_cru) <- c("lon","lat")
#head(na.omit(D_df02_cru, 5))
# create a dataframe without missing values. This will be used later to
demonstrate how to convert a "short"
# data frame into full matrix or array for writing out as a netCDF file.
#D_df02 <- na.omit(D_df02_cru)
#head(D_df02,2)
# remove lon and lat columns and work on new data frame for both
# NOTE to Self: "FYI D_df03 is prepared after this step so just make sure
you dont get confusued."
Var1 <-D_df02_hru$lon
Var2 <-D_df02_hru$lat
drops <- c("lon","lat")
dim(D_df02_hru)
D_df02_hru <- D_df02_hru[ , !(names(D_df02_hru) %in% drops)]
dim(D_df02_hru)
#drop the first 11 layers due to time scale
D_df02_hru<-D_df02_hru[,36:456]
dim(D_df02_hru)
D_df03_hru <- as.data.frame(t(D_df02_hru))
D_df04_hru <- D_df03_hru
Var1 <-D_df02_cru$lon
Var2 <-D_df02_cru$lat
drops <- c("lon","lat")
dim(D_df02_cru)
D_df02_cru <- D_df02_cru[ , !(names(D_df02_cru) %in% drops)]
dim(D_df02_cru)
#drop the first 11 layers due to time scale
D_df02_cru<-D_df02_cru[,36:456]
dim(D_df02_cru)
D_df03_cru <- as.data.frame(t(D_df02_cru))
D_df04_cru <- D_df03_cru
#-------------------------------------------Temporal Correlation---------
```

```
----------------------------------
correlation <- data.frame(NA)
data <- data.frame(hru="" ,cru="")
colnames(data) <- c("hru","cru")
for(i in seq(1,3366,1)){
data <- data.frame(hru = D_df04_hru[,i],cru = D_df04_cru[,i])
data <- data[is.finite(rowSums(data)),]
if(nrow(data) != 0){
r <- rcorr(data[,1],data[,2],type=c("pearson"))
if( r$P[2]<0.05){
correlation[i,] <- r$r[2]
}
else{
correlation[i,] <- NA
}
}
else{
correlation[i,] <-NA
}
}
#Add longitude and latitude back to the correlation dataframe
#t_correlation <-t(correlation)
spatial_correlation <- data.frame(lon=Var1,lat=Var2,correlation)
#spei_df05 <-
fread("E:/Thesis_2020/Analysis/Preprocessing/Deficit_values/spei_netcdfspei12.csv"
)#
spei_df05 <- data.frame(spei_df05)
#spei_36_corr_hru_cru_raster<-Raster_it(spatial_correlation)
#writeRaster(spei_36_corr_hru_cru_raster,"spei_36_corr_hru_cru_raster.tif",format="GTiff"
)
#spei_df06[is.na(spei_df06)] <- 1e32
#corr_array <- array(fillvalue_hru, dim=c(nlon_hru,nlat_hru,1))
corr_array2 <- array(spatial_correlation$NA., dim=c(nlon_hru,nlat_hru))
dim(corr_array2)
# quick map correlation
cutpts <- seq(-1,1,length.out=9)
grid <- expand.grid(lon=lon_hru, lat=lat_hru)
#image(lon_hru,lat_hru,corr_array2, data=grid, at=cutpts, cuts=10,
pretty=T,
# col.regions=(rev(brewer.pal(10,"RdBu"))), main="Correlation (r)")
spei_36_corr_hru_cru<-levelplot(corr_array2 ~ lon * lat, data=grid,
at=cutpts, cuts=10, pretty=T,
col.regions=(rev(brewer.pal(10,"RdYlBu"))))
#-----------------------------------Spatial Correlation-------------------
-------------------------
#depending on spei scale the length of time will change
temp_correlation <- data.frame(NA)
temp_data <- data.frame(hru="" ,cru="")
colnames(data) <- c("hru","cru")
dim(D_df02_cru)[2]
for(i in seq(1,dim(D_df02_cru)[2],1)){
temp_data <- data.frame(hru = D_df02_hru[,i],cru = D_df02_cru[,i])
temp_data <- temp_data[is.finite(rowSums(temp_data)),]
if(nrow(temp_data) != 0){
r <- rcorr(temp_data[,1],temp_data[,2],type=c("pearson"))
if( r$P[2]<0.05){
temp_correlation[i,] <- r$r[2]
}
```

```
else{
temp_correlation[i,] <- NA
}
}
else{
temp_correlation[i,] <-NA
}
}
ts_corr <- ts(temp_correlation, start=c(1983,12),end = c(2018,12),frequency
= 12)
months<-data.frame(NA)
#months<-data.frame(c(seq(1,37,1)))
#months <-
data.frame(Jan=c(1:38),Feb=c(1:38),Mar=c(1:38),Apr=c(1:38),May=c(1:38),Jun=c(1:38),Jul=c(1:38),Aug=c(1
:38),Sep=c(1:38),Oct=c(1:38),Nov=c(1:38),Dec=c(1:38)
)#
colnames(months) <-
c("Jan","Feb","Mar","Apr","May","Jun","Jul","Aug","Sep","Oct","Nov","Dec")
for (c in (1:12)) {
f <- which(cycle(ts_corr) == c)
f <- f[!is.na(ts_corr[f])]
#numeric <- as.data.frame(sort(ts_corr[f]))
numeric <- sort(ts_corr[f])
for(k in (1:length(numeric))){
months[k,c]<-numeric[k]
}
}
colnames(months) <-
c("Jan","Feb","Mar","Apr","May","Jun","Jul","Aug","Sep","Oct","Nov","Dec")
boxplot(months$Jan, months$Feb, months$Mar,
months$Apr,months$May,months$Jun,months$Jul,months$Aug,months$Sep,months$Oct,months$Nov,month
s$Dec
,
at = c(1,2,3,4,5,6,7,8,9,10,11,12),
names = c("Jan", "Feb", "Mar",
"Apr","May","Jun","Jul","Aug","Sep","Oct","Nov","Dec"),
col = "grey",
border = "black",
horizontal = FALSE,
notch = FALSE,
ylab = "R",
ylim = c(0.0,1.0)
)t
ext(0.0,"spei 36")
area_mean_36 <- data.frame(NA)
for(i in seq(1,dim(D_df02_cru)[2],1)){
interim_data <- data.frame(hru = D_df02_hru[,i],cru = D_df02_cru[,i])
interim_data <- interim_data[is.finite(rowSums(interim_data)),]
area_mean_36[i,1] <- mean(interim_data$hru)
area_mean_36[i,2] <- mean(interim_data$cru)
#area_mean_48[i,1] <- i
#area_mean_48[i,2] <- i
}
colnames(area_mean_36) <- c("hru","cru")
```

viii. corr_analysis_spei48_sci

```
library(ncdf4)
```

```
library(Hmisc)
#-----------------------------------------Spatial and Temporal Correlation
Analysis----------------------------------
#------------------------------------------------------Spatial
Correlation------------------------------------------
#collect data from resample HRU netcdf
# set path and filename
#ncpath <- "C:/Thesis2020/corr_analysis/"
ncpath <- "C:/Thesis2020/corr_analysis/corr_data/hru/"
#ncname <- "spei_48_hru_50km"
ncname <- "spei_48_hru_50km_green"
ncfname <- paste(ncpath, ncname, ".nc", sep="")
dname <- "spei" # note: precip means deficit (not precipitation)
# open a netCDF file
ncin <- nc_open(ncfname)
#print(ncin)
# get longitude and latitude
lon_hru <- ncvar_get(ncin,"lon")
nlon_hru <- dim(lon_hru)
head(lon_hru)
lat_hru <- ncvar_get(ncin,"lat")
nlat_hru <- dim(lat_hru)
head(lat_hru)
#print(c(nlon,nlat))
# get time
time_hru <- ncvar_get(ncin,"time")
#time_hru
tunits_hru <- ncatt_get(ncin,"time","units")
nt_hru <- dim(time_hru)
nt_hru
tunits_hru
# get spei from hru
spei_hru_array <- ncvar_get(ncin,dname)
splname_hru <- ncatt_get(ncin,dname,"long_name")
spunits_hru <- ncatt_get(ncin,dname,"units")
fillvalue_hru <- ncatt_get(ncin,dname,"_FillValue")
dim(spei_hru_array)
# get global attributes
#title <- ncatt_get(ncin,0,"title")
#institution <- ncatt_get(ncin,0,"institution")
#datasource <- ncatt_get(ncin,0,"source")
#references <- ncatt_get(ncin,0,"references")
#history <- ncatt_get(ncin,0,"history")
#Conventions <- ncatt_get(ncin,0,"Conventions")
nc_close(ncin)
# collect data from CRU netcdf
# set path and filename
#ncpath <- "C:/Thesis2020/corr_analysis/"
ncpath <- "C:/Thesis2020/corr_analysis/corr_data/cru/"
#ncname <- "spei_cru_48"
ncname <- "spei_cru_48_green"
ncfname <- paste(ncpath, ncname, ".nc", sep="")
dname <- "spei" # note: precip means deficit (not precipitation)
# open a netCDF file
ncin <- nc_open(ncfname)
#print(ncin)
# get longitude and latitude
lon_cru <- ncvar_get(ncin,"lon")
```

```
nlon_cru <- dim(lon_cru)
head(lon_cru)
lat_cru <- ncvar_get(ncin,"lat")
nlat_cru <- dim(lat_cru)
head(lat_cru)
#print(c(nlon,nlat))
# get time
time_cru <- ncvar_get(ncin,"time")
#time_cru
tunits_cru <- ncatt_get(ncin,"time","units")
nt_cru <- dim(time_cru)
nt_cru
tunits_cru
# get spei from hru
spei_cru_array <- ncvar_get(ncin,dname)
splname_cru <- ncatt_get(ncin,dname,"long_name")
spunits_cru <- ncatt_get(ncin,dname,"units")
fillvalue_cru <- ncatt_get(ncin,dname,"_FillValue")
dim(spei_cru_array)
# get global attributes
#title <- ncatt_get(ncin,0,"title")
#institution <- ncatt_get(ncin,0,"institution")
#datasource <- ncatt_get(ncin,0,"source")
#references <- ncatt_get(ncin,0,"references")
#history <- ncatt_get(ncin,0,"history")
#Conventions <- ncatt_get(ncin,0,"Conventions")
nc_close(ncin)
# load some packages
library(chron)
library(lattice)
library(RColorBrewer)
# convert time -- split the time units string into fields for HRU and then
CRU
tustr_hru <- strsplit(tunits_hru$value, " ")
tdstr_hru <- strsplit(unlist(tustr_hru)[3], "-")
tmonth_hru <- as.integer(unlist(tdstr_hru)[2])
tday_hru <- as.integer(unlist(tdstr_hru)[3])
tyear_hru <- as.integer(unlist(tdstr_hru)[1])
#chron(time_hru,origin=c(tmonth_hru, tday_hru, tyear_hru))
tustr_cru <- strsplit(tunits_cru$value, " ")
tdstr_cru <- strsplit(unlist(tustr_cru)[3], "-")
tmonth_cru <- as.integer(unlist(tdstr_cru)[2])
tday_cru <- as.integer(unlist(tdstr_cru)[3])
tyear_cru <- as.integer(unlist(tdstr_cru)[1])
#chron(time_cru,origin=c(tmonth_cru, tday_cru, tyear_cru))
# replace netCDF fill values with NA's for both HRU and CRU.
spei_hru_array[spei_hru_array==fillvalue_hru$value] <- NA
spei_cru_array[spei_cru_array==fillvalue_cru$value] <- NA
#depending on the spei scale the starting time with available values might
change. So for spei_6 we dont have any values in first 5 layers or slices.
length(na.omit(as.vector(spei_hru_array[,,48])))
length(na.omit(as.vector(spei_cru_array[,,48])))
# get a single slice or layer (January)
m <- 48
spei_hru_slice <- spei_hru_array[,,m]
spei_cru_slice <- spei_cru_array[,,m]
# quick map
#image(lon_hru,lat_hru,spei_hru_slice, col=rev(brewer.pal(10,"RdBu")))
```

```
#image(lon_cru,lat_cru,spei_cru_slice, col=rev(brewer.pal(10,"RdBu")))
# reshape the arrays into vector
spei_hru_vec_long <- as.vector(spei_hru_array)
length(spei_hru_vec_long)
spei_cru_vec_long <- as.vector(spei_cru_array)
length(spei_cru_vec_long)
# reshape the vectors into a matrix
spei_hru_mat <- matrix(spei_hru_vec_long, nrow=nlon_hru*nlat_hru,
ncol=nt_hru)
dim(spei_hru_mat)
spei_cru_mat <- matrix(spei_cru_vec_long, nrow=nlon_cru*nlat_cru,
ncol=nt_cru)
dim(spei_cru_mat)
#head(na.omit(D_mat))
# create a dataframe
lonlat_hru <- as.matrix(expand.grid(lon_hru,lat_hru))
D_df02_hru <- data.frame(cbind(lonlat_hru,spei_hru_mat))
names(D_df02_hru) <- c("lon","lat")
#head(na.omit(D_df02_hru))
lonlat_cru <- as.matrix(expand.grid(lon_cru,lat_cru))
D_df02_cru <- data.frame(cbind(lonlat_cru,spei_cru_mat))
names(D_df02_cru) <- c("lon","lat")
#head(na.omit(D_df02_cru, 5))
# create a dataframe without missing values. This will be used later to
demonstrate how to convert a "short"
# data frame into full matrix or array for writing out as a netCDF file.
#D_df02 <- na.omit(D_df02_cru)
#head(D_df02,2)
# remove lon and lat columns and work on new data frame for both
# NOTE to Self: "FYI D_df03 is prepared after this step so just make sure
you dont get confusued."
Var1 <-D_df02_hru$lon
Var2 <-D_df02_hru$lat
drops <- c("lon","lat")
dim(D_df02_hru)
D_df02_hru <- D_df02_hru[ , !(names(D_df02_hru) %in% drops)]
dim(D_df02_hru)
#drop the first 11 layers due to time scale
D_df02_hru<-D_df02_hru[,48:456]
dim(D_df02_hru)
D_df03_hru <- as.data.frame(t(D_df02_hru))
D_df04_hru <- D_df03_hru
Var1 <-D_df02_cru$lon
Var2 <-D_df02_cru$lat
drops <- c("lon","lat")
dim(D_df02_cru)
D_df02_cru <- D_df02_cru[ , !(names(D_df02_cru) %in% drops)]
dim(D_df02_cru)
#drop the first 11 layers due to time scale
D_df02_cru<-D_df02_cru[,48:456]
dim(D_df02_cru)
D_df03_cru <- as.data.frame(t(D_df02_cru))
D_df04_cru <- D_df03_cru
#-----------------------------------------------Temporal Correlation-----
----------------------------
correlation <- data.frame(NA)
data <- data.frame(hru="" ,cru="")
colnames(data) <- c("hru","cru")
```

```
for(i in seq(1,3366,1)){
data <- data.frame(hru = D_df04_hru[,i],cru = D_df04_cru[,i])
#data <- data.frame(hru = c(4,2,NA,4,7,8,-Inf,2,7,9,10),cru =
c(1,3,4,NA,3,6,1,3,5,8,-Inf))
#data2 <- data.frame(hru = c(NA,NA,NA,NA,NA,NA,NA,NA,NA,NA,NA),cru =
c(NA,NA,NA,NA,NA,NA,NA,NA,NA,NA,NA))
data <- data[is.finite(rowSums(data)),]
if(nrow(data) != 0){
r <- rcorr(data[,1],data[,2],type=c("pearson"))
if( r$P[2]<0.05){
correlation[i,] <- r$r[2]
}
else{
correlation[i,] <- NA
}
}
else{
correlation[i,] <-NA
}
}
#Add longitude and latitude back to the correlation dataframe
#t_correlation <-t(correlation)
spatial_correlation <- data.frame(lon=Var1,lat=Var2,correlation)
#spei_df05 <-
fread("E:/Thesis_2020/Analysis/Preprocessing/Deficit_values/spei_netcdfspei12.csv"
)#
spei_df05 <- data.frame(spei_df05)
#spei_48_corr_hru_cru_raster<-Raster_it(spatial_correlation)
#writeRaster(spei_48_corr_hru_cru_raster,"spei_48_corr_hru_cru_raster.tif",format="GTiff"
)
#spei_df06[is.na(spei_df06)] <- 1e32
#corr_array <- array(fillvalue_hru, dim=c(nlon_hru,nlat_hru,1))
corr_array2 <- array(spatial_correlation$NA., dim=c(nlon_hru,nlat_hru))
dim(corr_array2)
# quick map correlation
cutpts <- seq(-1,1,length.out=9)
grid <- expand.grid(lon=lon_hru, lat=lat_hru)
#image(lon_hru,lat_hru,corr_array2, data=grid, at=cutpts, cuts=10,
pretty=T,
# col.regions=(rev(brewer.pal(10,"RdBu"))), main="Correlation (r)")
spei_48_corr_hru_cru<-levelplot(corr_array2 ~ lon * lat, data=grid,
at=cutpts, cuts=10, pretty=T,
col.regions=(rev(brewer.pal(10,"RdYlBu"))))
#-----------------------------------Spatial Correlation-------------------
-------------------------
#depending on spei scale the length of time will change
temp_correlation <- data.frame(NA)
temp_data <- data.frame(hru="" ,cru="")
colnames(data) <- c("hru","cru")
dim(D_df02_cru)[2]
for(i in seq(1,dim(D_df02_cru)[2],1)){
temp_data <- data.frame(hru = D_df02_hru[,i],cru = D_df02_cru[,i])
#data <- data.frame(hru = c(4,2,NA,4,7,8,-Inf,2,7,9,10),cru =
c(1,3,4,NA,3,6,1,3,5,8,-Inf))
#data2 <- data.frame(hru = c(NA,NA,NA,NA,NA,NA,NA,NA,NA,NA,NA),cru =
c(NA,NA,NA,NA,NA,NA,NA,NA,NA,NA,NA))
temp_data <- temp_data[is.finite(rowSums(temp_data)),]
if(nrow(temp_data) != 0){
```

```
r <- rcorr(temp_data[,1],temp_data[,2],type=c("pearson"))
if( r$P[2]<0.05){
temp_correlation[i,] <- r$r[2]
}
else{
temp_correlation[i,] <- NA
}
}
else{
temp_correlation[i,] <-NA
}
}
ts_corr <- ts(temp_correlation, start=c(1984,12),end = c(2018,12),frequency
= 12)
months<-data.frame(NA)
#months<-data.frame(c(seq(1,37,1)))
#months <-
data.frame(Jan=c(1:38),Feb=c(1:38),Mar=c(1:38),Apr=c(1:38),May=c(1:38),Jun=c(1:38),Jul=c(1:38),Aug=c(1
:38),Sep=c(1:38),Oct=c(1:38),Nov=c(1:38),Dec=c(1:38)
)#
colnames(months) <-
c("Jan","Feb","Mar","Apr","May","Jun","Jul","Aug","Sep","Oct","Nov","Dec")
for (c in (1:12)) {
f <- which(cycle(ts_corr) == c)
f <- f[!is.na(ts_corr[f])]
#numeric <- as.data.frame(sort(ts_corr[f]))
#numeric <- sort(ts_corr[f])
numeric <- ts_corr[f]
for(k in (1:length(numeric))){
months[k,c]<-numeric[k]
}
}
colnames(months) <-
c("Jan","Feb","Mar","Apr","May","Jun","Jul","Aug","Sep","Oct","Nov","Dec")
boxplot(months$Jan, months$Feb, months$Mar,
months$Apr,months$May,months$Jun,months$Jul,months$Aug,months$Sep,months$Oct,months$Nov,month
s$Dec
,
at = c(1,2,3,4,5,6,7,8,9,10,11,12),
names = c("Jan", "Feb", "Mar",
"Apr","May","Jun","Jul","Aug","Sep","Oct","Nov","Dec"),
col = "grey",
border = "black",
horizontal = FALSE,
notch = FALSE,
ylab = "R",
ylim = c(0.0,1.0)
)t
ext(0.0,"spei 48")
area_mean_48 <- data.frame(NA)
for(i in seq(1,dim(D_df02_cru)[2],1)){
interim_data <- data.frame(hru = D_df02_hru[,i],cru = D_df02_cru[,i])
interim_data <- interim_data[is.finite(rowSums(interim_data)),]
area_mean_48[i,1] <- mean(interim_data$hru)
area_mean_48[i,2] <- mean(interim_data$cru)
#area_mean_48[i,1] <- i
#area_mean_48[i,2] <- i
}
```

```
colnames(area_mean_48) <- c("hru","cru")
#plot Ye told to make
plot(as.numeric(test_hru[,2:ncol(test_hru)]),ylim=c(-3,3),type='l')
lines(as.numeric(test_cru[,2:ncol(test_cru)]),type = 'l',col='blue')
```

ix. soilM_spatio_corr_sci

```
library(ncdf4)
library(Hmisc)
#-----------------------------------Root zone Soil Moisture Spatial and Temporal Correlataion Analysis--------
#collect data from resample soil moisture netcdf
# set path and filename
#ncpath <- "C:/Thesis2020/corr_analysis/"
ncpath <- "C:/Thesis2020/corr_analysis/corr_data/soil_moisture/"
#ncname <- "SM_81-18_50km"
ncname <- "SM_81-18_50km_green"
ncfname <- paste(ncpath, ncname, ".nc", sep="")
dname <- "SMroot" # note: precip means deficit (not precipitation)
# open a netCDF file
ncin <- nc_open(ncfname)
#print(ncin)
# get longitude and latitude
lon_sm <- ncvar_get(ncin,"lon")
nlon_sm <- dim(lon_sm)
head(lon_sm)
lat_sm <- ncvar_get(ncin,"lat")
nlat_sm <- dim(lat_sm)
head(lat_sm)
#print(c(nlon,nlat))
# get time
time_sm <- ncvar_get(ncin,"time")
#time_sm
tunits_sm <- ncatt_get(ncin,"time","units")
nt_sm <- dim(time_sm)
nt_sm
tunits_sm
# get spei from hru
sm_array <- ncvar_get(ncin,dname)
sm_name <- ncatt_get(ncin,dname,"long_name")
smunits <- ncatt_get(ncin,dname,"units")
fillvalue_sm <- ncatt_get(ncin,dname,"_FillValue")
dim(sm_array)
# get global attributes
#title <- ncatt_get(ncin,0,"title")
#institution <- ncatt_get(ncin,0,"institution")
#datasource <- ncatt_get(ncin,0,"source")
#references <- ncatt_get(ncin,0,"references")
#history <- ncatt_get(ncin,0,"history")
#Conventions <- ncatt_get(ncin,0,"Conventions")
nc_close(ncin)
# load some packages
library(chron)
library(lattice)
library(RColorBrewer)
# replace netCDF fill values with NA's for both HRU and CRU.
sm_array[sm_array==fillvalue_sm$value] <- NA
length(na.omit(as.vector(sm_array[,,1])))
# get a single slice or layer (January)
```

```
m <- 1
sm_slice <- sm_array[,,m]
# quick map
image(lon_sm,lat_sm, t(sm_slice), col=rev(brewer.pal(10,"RdBu")))
#sm_tslice<-t(sm_slice)
cutpts <- seq(0,0.5,length.out=11)
grid2 <- expand.grid(lat=lat_sm, lon=lon_sm)
levelplot(sm_slice ~ lon * lat, data=grid2, at=cutpts, cuts=10, pretty=T,
col.regions=(rev(brewer.pal(10,"RdBu"))), main="soil moisture")
# reshape the arrays into vector
sm_vec_long <- as.vector(sm_array)
length(sm_vec_long)
# reshape the vectors into a matrix
sm_mat <- matrix(sm_vec_long, nrow=nlon_sm*nlat_sm, ncol=nt_sm)
dim(sm_mat)
#head(na.omit(sm_mat))
# create a dataframe
lonlat_sm <- as.matrix(expand.grid(lat_sm,lon_sm))
D_df02_sm <- data.frame(cbind(lonlat_sm,sm_mat))
names(D_df02_sm) <- c("lat","lon")
dim(D_df02_sm)
#D_df02_sm_extra <- D_df02_sm
D_df02_sm <- D_df02_sm[order(D_df02_sm[,1]),]
# remove lon and lat columns and work on new data frame for both
Var1 <-D_df02_sm$lon
Var2 <-D_df02_sm$lat
drops <- c("lon","lat")
dim(D_df02_sm)
D_df02_sm <- D_df02_sm[ , !(names(D_df02_sm) %in% drops)]
dim(D_df02_sm)
#import the standardize_soilmoisture function before using the following
code
t_D_df02_sm_std <- standardize(D_df02_sm)
D_df02_sm_std <- as.data.frame(t(t_D_df02_sm_std))
D_df03_sm_std <- D_df02_sm_std[,6:456]
dim(D_df03_sm_std)
D_df04_sm_std <- as.data.frame(t(D_df03_sm_std))
dim(D_df04_sm_std)
#---------------------------------- Temporal Soil Moisture // Hru spei ---
------------------------------
# "Hru" from here onwards means High Resolution with a 5km by 5km grid
correlation_hrusm <- data.frame(NA)
data <- data.frame(hru="" ,sm="")
colnames(data) <- c("hru","sm")
for(i in seq(1,3366,1)){
j=3366-i
data <- data.frame(hru = D_df04_hru[,i],sm = D_df04_sm_std[,i])
data <- data[is.finite(rowSums(data)),]
if(nrow(data) != 0){
r <- rcorr(data[,1],data[,2],type=c("pearson"))
if( r$P[2]<0.05){
correlation_hrusm[i,] <- r$r[2]
}
else{
correlation_hrusm[i,] <-NA
}
}
else{
```

```
correlation_hrusm[i,] <-NA
}
}
#Add longitude and latitude back to the correlation dataframe
spatial_correlation <- data.frame(lat=Var2,lon=Var1,correlation_hrusm)
SM_hru_spei_6_corr_raster <- Raster_it(spatial_correlation)
crs(SM_hru_spei_6_corr_raster) <- "+proj=longlat +datum=WGS84 +no_defs"
corr_hrusm_array2 <- array(spatial_correlation$NA., dim=c(nlon_sm,nlat_sm))
dim(corr_hrusm_array2)
# quick map correlation
cutpts <- seq(-1,1,length.out=9)
grid <- expand.grid(lon=lon_sm,lat=lat_sm)
#image(lon_sm,lat_sm,corr_hrusm_array2, data=grid, at=cutpts, cuts=10,
pretty=T,
# col.regions=(rev(brewer.pal(10,"RdBu"))), main="Correlation (r)")
SM_hru_spei_6_corr<-levelplot(corr_hrusm_array2 ~ lon * lat, data=grid,
at=cutpts, cuts=10, pretty=T,
col.regions=(rev(brewer.pal(10,"RdYlBu")))) + CA2
#---------------------------------- Temporal Soil Moisture // Cru spei ---
------------------------------
correlation_crusm <- data.frame(NA)
data <- data.frame(cru="" ,sm="")
colnames(data) <- c("cru","sm")
for(i in seq(1,3366,1)){
data <- data.frame(cru = D_df04_cru[,i],sm = D_df04_sm_std[,i])
data <- data[is.finite(rowSums(data)),]
if(nrow(data) != 0){
r <- rcorr(data[,1],data[,2],type=c("pearson"))
if( r$P[2]<0.05){
correlation_crusm[i,] <- r$r[2]
}
else{
correlation_crusm[i,] <-NA
}
}
else{
correlation_crusm[i,] <-NA
}
}
#Add longitude and latitude back to the correlation dataframe
spatial_correlation2 <- data.frame(lat=Var2,lon=Var1,correlation_crusm)
SM_cru_spei_6_corr_raster <- Raster_it(spatial_correlation2)
crs(SM_cru_spei_6_corr_raster) <- "+proj=longlat +datum=WGS84 +no_defs"
corr_crusm_array2 <- array(spatial_correlation2$NA.,
dim=c(nlon_sm,nlat_sm))
dim(corr_crusm_array2)
# quick map correlation
cutpts <- seq(-1,1,length.out=9)
grid <- expand.grid(lon=lon_sm, lat=lat_sm)
#image(lon_sm,lat_sm,corr_hrusm_array2, data=grid, at=cutpts, cuts=10,
pretty=T,
#col.regions=(rev(brewer.pal(10,"RdBu"))), main="Correlation (r)")
SM_cru_spei_6_corr<-levelplot(corr_crusm_array2 ~ lon * lat, data=grid,
at=cutpts, cuts=10, pretty=T,
col.regions=(rev(brewer.pal(10,"RdYlBu")))) + CA2
grid.arrange(SM_hru_spei_6_corr,SM_cru_spei_6_corr,ncol=2)
#------------------------------------------ area mean correlation --------
-------------------------
```

```
area_mean<-data.frame(NA)
for(i in seq(1,456,1)){
area_mean[i,] <- mean(na.omit(D_df02_sm_std[,i]))
}
area_mean_sm <- area_mean
#correlation between area mean soil moisture per year and spei 1 hru and
cru
rcorr(area_mean_1[,1],area_mean_sm[,1],type=c("pearson"))
rcorr(area_mean_1[,2],area_mean_sm[,1],type=c("pearson"))
#correlation between area mean soil moisture per year and spei 3 hru and
cru
rcorr(area_mean_3[,1],area_mean_sm[3:456,1],type=c("pearson"))
rcorr(area_mean_3[,2],area_mean_sm[3:456,1],type=c("pearson"))
#correlation between area mean soil moisture per year and spei 6 hru and
cru
rcorr(area_mean_6[,1],area_mean_sm[6:456,1],type=c("pearson"))
rcorr(area_mean_6[,2],area_mean_sm[6:456,1],type=c("pearson"))
#correlation between area mean soil moisture per year and spei 9 hru and
cru
rcorr(area_mean_9[,1],area_mean_sm[9:456,1],type=c("pearson"))
rcorr(area_mean_9[,2],area_mean_sm[9:456,1],type=c("pearson"))
#correlation between area mean soil moisture per year and spei 12 hru and
cru
rcorr(area_mean_12[,1],area_mean_sm[12:456,1],type=c("pearson"))
rcorr(area_mean_12[,2],area_mean_sm[12:456,1],type=c("pearson"))
#correlation between area mean soil moisture per year and spei 24 hru and
cru
rcorr(area_mean_24[,1],area_mean_sm[24:456,1],type=c("pearson"))
rcorr(area_mean_24[,2],area_mean_sm[24:456,1],type=c("pearson"))
#correlation between area mean soil moisture per year and spei 36 hru and
cru
rcorr(area_mean_36[,1],area_mean_sm[36:456,1],type=c("pearson"))
rcorr(area_mean_36[,2],area_mean_sm[36:456,1],type=c("pearson"))
#correlation between area mean soil moisture per year and spei 48 hru and
cru
rcorr(area_mean_48[,1],area_mean_sm[48:456,1],type=c("pearson"))
rcorr(area_mean_48[,2],area_mean_sm[48:456,1],type=c("pearson"))
#----------------------plot for time scale 6 months----------------------
dates <- seq(as.Date("1981/1/1"), by = "month", length.out = 456)
plot(dates[6:456],area_mean_sm$NA.[06:456],type =
'l',col='green',ylab="Root zone soil moisture",xlab="date",
xlim=c(as.dates(time_sm)[6],as.dates(time_sm)[456]),lwd=2)
lines(dates[6:456],area_mean_6$hru,type = 'l',col='red',lwd=2)
lines(dates[6:456],area_mean_6$cru,type = 'l',col='blue',lwd=2)
text(locator(1),"SPEI-HR and RSM:R = 0.66 \nSPEI-CRU and RSM:R = 0.71",
cex=0.75)
legend(locator(2), legend=c("RSM", "SPEI-HR","SPEI-CRU"),col=c("green",
"red","blue"), lty=1, cex=0.75)
#-------------------- CA2 outline-------------------------
library(sf)
library(gridExtra)
library(sp)
shp_path <- "E:/Thesis_2020/Analysis/Preprocessing/centralasia
shapefile/2nd CA shape file from
Ye/BoundariesofCA_No_riverandsea/BoundariesofCA_No_riverandsea/"
shp_name <- "BoundariesofCA_No_riverandsea.shp"
shp_file <- paste(shp_path, shp_name, sep="")
# read the shapefile
```

```
CA_2_shp <- read_sf(shp_file)
CA_2_outline <- as(st_geometry(CA_2_shp), Class="Spatial")
CA2<-latticeExtra::layer(sp.lines(CA_2_outline, col="black", lwd=1.0))
#-----------------------------levelplot for Rasters-----------------------
-----
library(rasterVis)
library(RColorBrewer)
library(gridExtra)
cutpts <- seq(-1,1,length.out=9)
SM_cru_lvlplt <- levelplot(SM_cru_spei_6_corr_raster,at=cutpts, cuts=10,
pretty=T,
col.regions=(rev(brewer.pal(10,"RdYlBu"))),margin=FALSE) + CA2
SM_cru_lvlplt[["y.limits"]]<- c(34.0, 50.5)
SM_hru_lvlplt <- levelplot(SM_hru_spei_6_corr_raster,at=cutpts, cuts=10,
pretty=T,
col.regions=(rev(brewer.pal(10,"RdYlBu"))),margin=FALSE) + CA2
SM_hru_lvlplt[["y.limits"]]<- c(34.0, 50.5)
grid.arrange(SM_hru_lvlplt,SM_cru_lvlplt,ncol=2)
png("C:/Thesis2020/corr_analysis/Results/Greenland/soil
moisture/area_mean_res300_rev02.png",width=210,height=297/3,units="mm",res=300,bg="white"
)
#dates <- seq(as.Date("1981/1/1"), by = "month", length.out = 456)
plot(dates[6:456],area_mean_sm$NA.[06:456],type =
'l',col='green',ylab="Area mean",xlab="Date", lwd=2, ylim=c(-2,2))
lines(dates[6:456],area_mean_6$hru,type = 'l',col='red',lwd=2)
lines(dates[6:456],area_mean_6$cru,type = 'l',col='blue',lwd=2)
text(l1,"SPEI-HR and RSM:R = 0.66 \nSPEI-CRU and RSM:R = 0.71", cex=0.75)
legend(l2, legend=c("RSM", "SPEI-HR","SPEI-CRU"),col=c("green",
"red","blue"), lty=1, cex=0.75)
dev.off()
dates <- seq(as.Date("1980/1/1"), by = "month", length.out = 432)
plot(dates[6:420],area_mean_ndvi$NA.[6:420],type =
'l',col='green',ylab="Area mean",xlab="Date",lwd=2,ylim=c(-2,2))
lines(dates[6:420],area_mean_6$hru,type = 'l',col='red',lwd=2)
lines(dates[6:420],area_mean_6$cru,type = 'l',col='blue',lwd=2)
text(l1,"SPEI-HR and NDVI:R = 0.26 \nSPEI-CRU and NDVI:R = 0.30", cex=0.75)
legend(l2, legend=c("NDVI", "NDVI-HR","NDVI-CRU"),col=c("green",
"red","blue"), lty=1, cex=0.75)
```

x. ndvi_rev03_sci

```
setwd("C:/Thesis2020/ndvi/raw")
# load packages
library(sf)
library(ncdf4)
library(raster)
library(rasterVis)
library(RColorBrewer)
library(Hmisc)
# set path and shape file name
shp_path <- "C:/Thesis2020/arccatalog/"
shp_name <- "CA_clipped.shp"
shp_file <- paste(shp_path, shp_name, sep="")
green_shp_path <- "C:/Thesis2020/Modis_lc/CA_3/mask/"
green_shp_name <- "CA_notbarren2.shp"
green_shp_file <- paste(green_shp_path, green_shp_name, sep="")
# read the shapefile
```

```
CA_shp <- read_sf(shp_file)
CA_outline <- as(st_geometry(CA_shp), Class="Spatial")
CA_green_shp <- read_sf(green_shp_file)
CA_green_outline <- as(st_geometry(CA_green_shp), Class="Spatial")
# plot the outline
plot(CA_outline, col="gray80", lwd=1)
#plot(CA_nb_outline, col="gray80", lwd=1)
plot(CA_green_outline, col="gray80", lwd=1)
# set path and list .nc files
nclist<-list.files("C:/Thesis2020/ndvi/raw",pattern = ".nc4$")
#read 3D array from the netcdf file using the stack() fnction
ndvi_raster <- stack(nclist,varname="ndvi")
# initial plot
mapTheme <- rasterTheme(region = rev(brewer.pal(10, "RdYlBu")))
cutpts <- c(0,1000,2000,3000,4000.5000,6000,7000,8000,9000,10000)
plt<-levelplot(subset(ndvi_raster,13), margin = F, at=cutpts, cuts=11,
pretty=TRUE, par.settings = mapTheme,
main="ndvi")
plt + latticeExtra::layer(sp.lines(CA_outline, col="black", lwd=1.0))
# fillvalues to be replaced with NA, not possible for the whole data set
therefore we first crop and mask the data to reduce size.
CA_ndvi<- crop(ndvi_raster,CA_outline)
CA_ndvi<- raster::mask(CA_ndvi,CA_outline)
#can take 30min
CA_ndvi<-raster::mask(CA_ndvi,CA_green_outline)
# scale the ndvi data Takes almost 1 hour
CA_ndvi_s <-CA_ndvi/10000
CA_ndvi_s[CA_ndvi_s < 0] <- NA
# after filling NA we can regrid the data to bring the resolution down to
cru data. To do that first we need to
# read CRU netcdf file and crop and mask it to the region of interest
# read CRU netcdf for regriding the data
ncpath <- "C:/Thesis2020/CRU/PET_cru/raw_data/"
ncname <- "cru_ts4.04.1981.1990.pet.dat"
ncfname <- paste(ncpath, ncname, ".nc", sep="")
cru_raster <- brick(ncfname, varname="pet")
#crop and mask it to the region of interest
CA_cru<- crop(cru_raster,CA_outline)
CA_cru<- raster::mask(CA_cru,CA_outline)
# Regrid scaled ndvi data to 50 km CRU resolution
CA_ndvi_s_remap <- projectRaster(CA_ndvi_s,CA_cru,method = 'bilinear')
# plot to regrided data
mapTheme <- rasterTheme(region = rev(brewer.pal(10, "RdBu")))
cutpts <- c(0,0.1,0.2,0.3,0.4,0.5,0.6,0.7,0.8,0.9,1)
plt<-levelplot(subset(CA_ndvi_s_remap,18), margin = F, at=cutpts, cuts=11,
pretty=TRUE, par.settings = mapTheme,
main="ndvi")
plt + latticeExtra::layer(sp.lines(CA_outline, col="black", lwd=1.0))
# for back up write as Gtiff for future use
writeRaster(CA_ndvi_s_remap,"CA_ndvi_s_cru_remap",format="GTiff")
CA_ndvi_s_remap<-raster("C:/Thesis2020/ndvi/raw/CA_ndvi_s_cru_remap.tif")
#coordinates
coords <- xyFromCell(CA_ndvi_s_remap,1:ncell(CA_ndvi_s_remap))
#create list
xyzlist <- list(x=coords[,'x'],y=coords[,'y'],z=as.matrix(CA_ndvi_s_remap))
#create dataframe
df_ndvi<-data.frame(xyzlist$x,xyzlist$y,xyzlist$z)
View(df_ndvi)
```

```
# before correlation, ordering the data w.r.t lat is important to match the
coordinates of cru data frame
# we must remove the lat 34.25 (102) and 50.25 (102) and the lon 46.25(31)
and 96.75(31). After removal we will have
# matching lengths of dataframes with matching pixels. This could be
achieved by ordering the data frame and removing data
# FYI: for ploting the data we do not need ordered dataframe because other
wise the array conversion is not proper
df_ndvi_order <- df_ndvi[order(df_ndvi[,2]),]
ndvi_array <- array(df_ndvi$layer.7, dim=c(100,31))
dim(ndvi_array)
# quick map correlation
cutpts <- seq(-1,1,length.out=9)
grid <- data.frame(lon=coords[,'x'],lat=coords[,'y'])
levelplot(ndvi_array ~ lon * lat, data=grid, at=cutpts, cuts=10, pretty=T,
col.regions=(rev(brewer.pal(10,"RdBu"))), main="ndvi")
# removing pixels from cru and hru data, for the lon and lat which is not
present in ndvi data. Import D_df02 tables
# from the "corr_analysis_spei6_sci" script. Make sure the tables have lon
and lat in them.
D_df02_cru_r <- D_df02_cru[103:3264,]
D_df02_hru_r <- D_df02_hru[103:3264,]
D_df02_cru_r <- D_df02_cru_r[order(D_df02_cru_r[,1]),]
D_df02_hru_r <- D_df02_hru_r[order(D_df02_hru_r[,1]),]
D_df02_cru_r <- D_df02_cru_r[32:3131,]
D_df02_hru_r <- D_df02_hru_r[32:3131,]
D_df02_cru_r <- D_df02_cru_r[order(D_df02_cru_r[,2]),]
D_df02_hru_r <- D_df02_hru_r[order(D_df02_hru_r[,2]),]
# removing lon and lat from the data frames for further processing
Var1 <-D_df02_hru_r$lon
Var2 <-D_df02_hru_r$lat
drops <- c("lon","lat")
dim(D_df02_hru_r)
D_df02_hru_r <- D_df02_hru_r[ , !(names(D_df02_hru_r) %in% drops)]
dim(D_df02_hru_r)
#drop the first timescale - 1 layers due to time scale
D_df02_hru_r<-D_df02_hru_r[,6:420]
dim(D_df02_hru_r)
D_df03_hru_r <- as.data.frame(t(D_df02_hru_r))
D_df04_hru_r <- D_df03_hru_r
Var1 <-D_df02_cru_r$lon
Var2 <-D_df02_cru_r$lat
drops <- c("lon","lat")
#D_df02 <- D_df03
dim(D_df02_cru_r)
D_df02_cru_r <- D_df02_cru_r[ , !(names(D_df02_cru_r) %in% drops)]
dim(D_df02_cru_r)
#drop the first timescale - 1 layers due to time scale
D_df02_cru_r<-D_df02_cru_r[,6:420]
dim(D_df02_cru_r)
D_df03_cru_r <- as.data.frame(t(D_df02_cru_r))
D_df04_cru_r <- D_df03_cru_r
Var1 <-df_ndvi_order$xyzlist.x
Var2 <-df_ndvi_order$xyzlist.y
drops <- c("xyzlist.x","xyzlist.y")
#D_df02 <- D_df03
dim(df_ndvi_order)
df_ndvi_order <- df_ndvi_order[ , !(names(df_ndvi_order) %in% drops)]
```

```
dim(df_ndvi_order)
#import the standardize_nvi function before using the following code
t_df_ndvi_std <- standardize(df_ndvi_order)
df_ndvi_std <- as.data.frame(t(t_df_ndvi_std))
df2_ndvi_std <- df_ndvi_std[,6:420]
#df2_ndvi_std <- df_ndvi_std
dim(df2_ndvi_std)
df3_ndvi_std <- as.data.frame(t(df2_ndvi_std))
dim(df3_ndvi_std)
#---------------------------------- Temporal Soil Moisture // Hru spei ---
------------------------------
correlation_hrusm <- data.frame(NA)
data <- data.frame(hru="" ,ndvi="")
colnames(data) <- c("hru","ndvi")
for(i in seq(1,3100,1)){
j=3100-i
data <- data.frame(hru = D_df04_hru_r[,i],ndvi = df3_ndvi_std[,i])
data <- data[is.finite(rowSums(data)),]
if(nrow(data) != 0){
r <- rcorr(data[,1],data[,2],type=c("pearson"))
if( r$P[2]<0.05){
correlation_hrusm[i,] <- r$r[2]
}
else{
correlation_hrusm[i,] <-NA
}
}
else{
correlation_hrusm[i,] <-NA
}
}
#Add longitude and latitude back to the correlation dataframe
spatial_correlation <- data.frame(lat=Var2,lon=Var1,correlation_hrusm)
NDVI_hru_corr_raster <- Raster_it(spatial_correlation)
crs(NDVI_hru_corr_raster) <- "+proj=longlat +datum=WGS84 +no_defs"
setwd("C:/Thesis2020/corr_analysis/Results/Greenland/ndvi")
writeRaster(NDVI_hru_corr_raster,"NDVI_hru_corr_raster.tif",format="GTiff")
corr_hrusm_array2 <- array(spatial_correlation$NA., dim=c(100,31))
dim(corr_hrusm_array2)
# quick map correlation
cutpts <- seq(-1,1,length.out=9)
grid <- data.frame(lon=Var1,lat=Var2)
#image(lon_sm,lat_sm,corr_hrusm_array2, data=grid, at=cutpts, cuts=10,
pretty=T,
# col.regions=(rev(brewer.pal(10,"RdBu"))), main="Correlation (r)")
NDVI_hru_spei_6_corr<-levelplot(corr_hrusm_array2 ~ lon * lat, data=grid,
at=cutpts, cuts=10, pretty=T,
col.regions=(rev(brewer.pal(10,"RdYlBu")))) + CA2
#---------------------------------- Temporal Soil Moisture // Cru spei ---
------------------------------
correlation_crusm <- data.frame(NA)
data <- data.frame(cru="" ,ndvi="")
colnames(data) <- c("cru","ndvi")
for(i in seq(1,3100,1)){
data <- data.frame(cru = D_df04_cru_r[,i],sm = df3_ndvi_std[,i])
data <- data[is.finite(rowSums(data)),]
if(nrow(data) != 0){
r <- rcorr(data[,1],data[,2],type=c("pearson"))
```

```
if( r$P[2]<0.05){
correlation_crusm[i,] <- r$r[2]
}
else{
correlation_crusm[i,] <-NA
}
}
else{
correlation_crusm[i,] <-NA
}
}
#Add longitude and latitude back to the correlation dataframe
spatial_correlation2 <- data.frame(lat=Var2,lon=Var1,correlation_crusm)
NDVI_cru_corr_raster <- Raster_it(spatial_correlation2)
crs(NDVI_cru_corr_raster) <- "+proj=longlat +datum=WGS84 +no_defs"
setwd("C:/Thesis2020/corr_analysis/Results/Greenland/ndvi")
writeRaster(NDVI_cru_corr_raster,"NDVI_cru_corr_raster.tif",format="GTiff")
corr_crusm_array2 <- array(spatial_correlation2$NA., dim=c(100,31))
dim(corr_crusm_array2)
# quick map correlation
cutpts <- seq(-1,1,length.out=9)
grid <- data.frame(lon=Var1,lat=Var2)
#image(lon_sm,lat_sm,corr_hrusm_array2, data=grid, at=cutpts, cuts=10,
pretty=T,
#col.regions=(rev(brewer.pal(10,"RdBu"))), main="Correlation (r)")
NDVI_cru_spei_6_corr<-levelplot(corr_crusm_array2 ~ lon * lat, data=grid,
at=cutpts, cuts=10, pretty=T,
col.regions=(rev(brewer.pal(10,"RdYlBu")))) + CA2
grid.arrange(NDVI_hru_spei_6_corr,NDVI_cru_spei_6_corr,ncol=2)
#------------------------------------------ area mean correlation --------
-------------------------
df2_ndvi_std <- df_ndvi_std
area_mean<-data.frame(NA)
for(i in seq(1,420,1)){
area_mean[i,] <- mean(na.omit(df2_ndvi_std[,i]))
}
area_mean_ndvi <- area_mean
#correlation between area mean soil moisture per year and spei 1 hru and
cru
area_mean_1 <- data.frame(NA)
for(i in seq(1,dim(D_df02_cru_r)[2],1)){
interim_data <- data.frame(hru = D_df02_hru_r[,i],cru = D_df02_cru_r[,i])
interim_data <- interim_data[is.finite(rowSums(interim_data)),]
area_mean_1[i,1] <- mean(interim_data$hru)
area_mean_1[i,2] <- mean(interim_data$cru)
}
colnames(area_mean_1) <- c("hru","cru")
rcorr(area_mean_1[,1],area_mean_ndvi[,1],type=c("pearson"))
rcorr(area_mean_1[,2],area_mean_ndvi[,1],type=c("pearson"))
#correlation between area mean soil moisture per year and spei 3 hru and
cru
area_mean_3 <- data.frame(NA)
for(i in seq(1,dim(D_df02_cru_r)[2],1)){
interim_data <- data.frame(hru = D_df02_hru_r[,i],cru = D_df02_cru_r[,i])
interim_data <- interim_data[is.finite(rowSums(interim_data)),]
area_mean_3[i,1] <- mean(interim_data$hru)
area_mean_3[i,2] <- mean(interim_data$cru)
}
```

```
colnames(area_mean_3) <- c("hru","cru")
rcorr(area_mean_3[,1],area_mean_ndvi[3:420,1],type=c("pearson"))
rcorr(area_mean_3[,2],area_mean_ndvi[3:420,1],type=c("pearson"))
#correlation between area mean soil moisture per year and spei 6 hru and
cru
area_mean_6 <- data.frame(NA)
for(i in seq(1,dim(D_df02_cru_r)[2],1)){
interim_data <- data.frame(hru = D_df02_hru_r[,i],cru = D_df02_cru_r[,i])
interim_data <- interim_data[is.finite(rowSums(interim_data)),]
area_mean_6[i,1] <- mean(interim_data$hru)
area_mean_6[i,2] <- mean(interim_data$cru)
}
colnames(area_mean_6) <- c("hru","cru")
rcorr(area_mean_6[,1],area_mean_ndvi[6:420,1],type=c("pearson"))
rcorr(area_mean_6[,2],area_mean_ndvi[6:420,1],type=c("pearson"))
#correlation between area mean soil moisture per year and spei 9 hru and
cru
area_mean_9 <- data.frame(NA)
for(i in seq(1,dim(D_df02_cru_r)[2],1)){
interim_data <- data.frame(hru = D_df02_hru_r[,i],cru = D_df02_cru_r[,i])
interim_data <- interim_data[is.finite(rowSums(interim_data)),]
area_mean_9[i,1] <- mean(interim_data$hru)
area_mean_9[i,2] <- mean(interim_data$cru)
}
colnames(area_mean_9) <- c("hru","cru")
rcorr(area_mean_9[,1],area_mean_ndvi[9:420,1],type=c("pearson"))
rcorr(area_mean_9[,2],area_mean_ndvi[9:420,1],type=c("pearson"))
#correlation between area mean soil moisture per year and spei 12 hru and
cru
area_mean_12 <- data.frame(NA)
for(i in seq(1,dim(D_df02_cru_r)[2],1)){
interim_data <- data.frame(hru = D_df02_hru_r[,i],cru = D_df02_cru_r[,i])
interim_data <- interim_data[is.finite(rowSums(interim_data)),]
area_mean_12[i,1] <- mean(interim_data$hru)
area_mean_12[i,2] <- mean(interim_data$cru)
}
colnames(area_mean_12) <- c("hru","cru")
rcorr(area_mean_12[,1],area_mean_ndvi[12:420,1],type=c("pearson"))
rcorr(area_mean_12[,2],area_mean_ndvi[12:420,1],type=c("pearson"))
#correlation between area mean soil moisture per year and spei 24 hru and
cru
area_mean_24 <- data.frame(NA)
for(i in seq(1,dim(D_df02_cru_r)[2],1)){
interim_data <- data.frame(hru = D_df02_hru_r[,i],cru = D_df02_cru_r[,i])
interim_data <- interim_data[is.finite(rowSums(interim_data)),]
area_mean_24[i,1] <- mean(interim_data$hru)
area_mean_24[i,2] <- mean(interim_data$cru)
}
colnames(area_mean_24) <- c("hru","cru")
rcorr(area_mean_24[,1],area_mean_ndvi[24:420,1],type=c("pearson"))
rcorr(area_mean_24[,2],area_mean_ndvi[24:420,1],type=c("pearson"))
#correlation between area mean soil moisture per year and spei 36 hru and
cru
area_mean_36 <- data.frame(NA)
for(i in seq(1,dim(D_df02_cru_r)[2],1)){
interim_data <- data.frame(hru = D_df02_hru_r[,i],cru = D_df02_cru_r[,i])
interim_data <- interim_data[is.finite(rowSums(interim_data)),]
area_mean_36[i,1] <- mean(interim_data$hru)
```

```
area_mean_36[i,2] <- mean(interim_data$cru)
}
colnames(area_mean_36) <- c("hru","cru")
rcorr(area_mean_36[,1],area_mean_ndvi[36:420,1],type=c("pearson"))
rcorr(area_mean_36[,2],area_mean_ndvi[36:420,1],type=c("pearson"))
#correlation between area mean soil moisture per year and spei 48 hru and
cru
area_mean_48 <- data.frame(NA)
for(i in seq(1,dim(D_df02_cru_r)[2],1)){
interim_data <- data.frame(hru = D_df02_hru_r[,i],cru = D_df02_cru_r[,i])
interim_data <- interim_data[is.finite(rowSums(interim_data)),]
area_mean_48[i,1] <- mean(interim_data$hru)
area_mean_48[i,2] <- mean(interim_data$cru)
}
colnames(area_mean_48) <- c("hru","cru")
rcorr(area_mean_48[,1],area_mean_ndvi[48:420,1],type=c("pearson"))
rcorr(area_mean_48[,2],area_mean_ndvi[48:420,1],type=c("pearson"))
#----------------------plot for time scale 6 months----------------------
dates <- seq(as.Date("1980/1/1"), by = "month", length.out = 432)
plot(dates[6:420],area_mean_ndvi$NA.[6:420],type =
'l',col='green',ylab="area mean",xlab="date",lwd=2,ylim=c(-2,2))
lines(dates[6:420],area_mean_6$hru,type = 'l',col='red',lwd=2)
lines(dates[6:420],area_mean_6$cru,type = 'l',col='blue',lwd=2)
text(locator(1),"SPEI-HR and NDVI:R = 0.26 \nSPEI-CRU and NDVI:R = 0.30",
cex=0.75)
legend(locator(2), legend=c("NDVI", "SPEI-HR","SPEI-CRU"),col=c("green",
"red","blue"), lty=1, cex=0.75)
#-------------------- CA2 outline-------------------------
library(sf)
library(gridExtra)
library(sp)
shp_path <- "E:/Thesis_2020/Analysis/Preprocessing/centralasia
shapefile/2nd CA shape file from
Ye/BoundariesofCA_No_riverandsea/BoundariesofCA_No_riverandsea/"
shp_name <- "BoundariesofCA_No_riverandsea.shp"
shp_file <- paste(shp_path, shp_name, sep="")
# read the shapefile
CA_2_shp <- read_sf(shp_file)
CA_2_outline <- as(st_geometry(CA_2_shp), Class="Spatial")
CA2<-latticeExtra::layer(sp.lines(CA_2_outline, col="black", lwd=1.0))
#-----------------------------levelplot for Rasters----------------------
-----
library(rasterVis)
NDVI_cru_spei6_corr_raster <-
raster("C:/Thesis2020/corr_analysis/Results/Greenland/ndvi/NDVI_cru_corr_raster.tif"
)
cutpts <- seq(-1,1,length.out=9)
NDVI_cru_lvlplt <- levelplot(NDVI_cru_corr_raster,at=cutpts, cuts=10,
pretty=T,
col.regions=(rev(brewer.pal(10,"RdYlBu"))),margin=FALSE) + CA2
NDVI_cru_lvlplt[["y.limits"]]<- c(34.0, 50.5)
NDVI_hru_lvlplt <- levelplot(NDVI_hru_corr_raster,at=cutpts, cuts=10,
pretty=T,
col.regions=(rev(brewer.pal(10,"RdYlBu"))),margin=FALSE) + CA2
NDVI_hru_lvlplt[["y.limits"]]<- c(34.0, 50.5)
grid.arrange(NDVI_hru_lvlplt,NDVI_cru_lvlplt,ncol=2)
png("C:/Thesis2020/corr_analysis/Results/Greenland/ndvi/area_mean_res300_rev04.png",width=210,height=
297/3,units="mm",res=300,bg="white"
```

```
)
dates <- seq(as.Date("1980/1/1"), by = "month", length.out = 432)
plot(dates[6:420],area_mean_ndvi$NA.[6:420],type =
'l',col='green',ylab="Area mean",xlab="Date",lwd=2,ylim=c(-2,2))
lines(dates[6:420],area_mean_6$hru,type = 'l',col='red',lwd=2)
lines(dates[6:420],area_mean_6$cru,type = 'l',col='blue',lwd=2)
text(l1,"SPEI-HR and NDVI:R = 0.26 \nSPEI-CRU and NDVI:R = 0.30", cex=0.75)
legend(l2, legend=c("NDVI", "NDVI-HR","NDVI-CRU"),col=c("green",
"red","blue"), lty=1, cex=0.75)
dev.off()
```

xi. standardize_ndvi_sci

```
standardize<-function(DF_soilmoisture){
D_df02_sm<-DF_soilmoisture
t_D_df02<-as.data.frame(t(D_df02_sm))
#ini_try<-data.frame(matrix(NA,nrow = 420,ncol = 2))
#q=0
for(i in seq(1,3100,1)){
pixel<-t_D_df02[,i]
pixel <- na.omit(pixel)
#q=q+1
if(length(pixel) != 0){
#pixel <- data
ts_pixel <- ts(pixel,start = c(1981,1),end = c(2015,12), frequency =
12)
months <- data.frame(NA)
for (c in (1:12)) {
f <- which(cycle(ts_pixel) == c)
f <- f[!is.na(ts_pixel[f])]
#numeric <- as.data.frame(sort(ts_corr[f]))
#numeric <- sort(ts_pixel[f])
numeric<-(ts_pixel[f])
for(k in (1:length(numeric))){
months[k,c]<-numeric[k]
}
}
months2<-months
#for(k in seq(1,6,1)){
#months2[2:38,k]<-months2[1:37,k]
#months2[1,k]<- NA
#}
for(j in seq(1,12,1)){
m<-mean(months2[,j])
s_dev<-sd(months2[,j])
for(x in seq(1,35,1)){
#months2[x,j] <- ((months2[x,j]-
mean(na.omit(months2[,j])))/sd(na.omit(months2[,j])))
if(!is.na(months2[x,j])){
months2[x,j] <- ((months2[x,j]-m)/s_dev)
}
else{
months2[x,j] <- NA
}
}
}
t_months <- t(months2)
vec_tmonths <- as.vector(t_months)
mat_tmonths <- as.matrix(vec_tmonths,nrow=456,ncol=1)
#ini_try[,q] <- data.frame(mat_tmonths)
t_D_df02[,i] <- data.frame(mat_tmonths)
}
```

```
#else{
#ini_try[,q] <- pixel
#t_D_df02[,i] <- pixel
#}
}
return(t_D_df02)
}
#---------------------------------------------------------------- x -----------------------------------------
```

xii. standardize_soilmoisture_sci

```
standardize<-function(DF_soilmoisture){
D_df02_sm<-DF_soilmoisture
t_D_df02<-as.data.frame(t(D_df02_sm))
#ini_try<-data.frame(matrix(NA,nrow = 456,ncol = 2))
#q=0
for(i in seq(1,3366,1)){
pixel<-t_D_df02[,i]
pixel <- na.omit(pixel)
#q=q+1
if(length(pixel) != 0){
#pixel <- data
ts_pixel <- ts(pixel,start = c(1981,1),end = c(2018,12), frequency =
12)
months <- data.frame(NA)
for (c in (1:12)) {
f <- which(cycle(ts_pixel) == c)
f <- f[!is.na(ts_pixel[f])]
#numeric <- as.data.frame(sort(ts_corr[f]))
#numeric <- sort(ts_pixel[f])
numeric<-(ts_pixel[f])
for(k in (1:length(numeric))){
months[k,c]<-numeric[k]
}
}
months2<-months
#for(k in seq(1,6,1)){
#months2[2:38,k]<-months2[1:37,k]
#months2[1,k]<- NA
#}
for(j in seq(1,12,1)){
m<-mean(months2[,j])
s_dev<-sd(months2[,j])
for(x in seq(1,38,1)){
#months2[x,j] <- ((months2[x,j]-
mean(na.omit(months2[,j])))/sd(na.omit(months2[,j])))
if(!is.na(months2[x,j])){
months2[x,j] <- ((months2[x,j]-m)/s_dev)
}
else{
months2[x,j] <- NA
}
}
}
t_months <- t(months2)
vec_tmonths <- as.vector(t_months)
mat_tmonths <- as.matrix(vec_tmonths,nrow=456,ncol=1)
#ini_try[,q] <- data.frame(mat_tmonths)
t_D_df02[,i] <- data.frame(mat_tmonths)
}
#else{
#ini_try[,q] <- pixel
```

```
#t_D_df02[,i] <- pixel
#}
}
return(t_D_df02)
}
#------------------------------------------------------------------ x -----------------------------------------
```

xiii. Functions

1. NDVI

```
standardize<-function(DF_soilmoisture){
  D_df02_sm<-DF_soilmoisture
  t_D_df02<-as.data.frame(t(D_df02_sm))
  #ini_try<-data.frame(matrix(NA,nrow = 420,ncol = 2))
  #q=0
  for(i in seq(1,3100,1)){
    pixel<-t_D_df02[,i]
    pixel <- na.omit(pixel)
    #q=q+1
    if(length(pixel) != 0){
      #pixel <- data
      ts_pixel <- ts(pixel,start = c(1981,1),end = c(2015,12), frequency = 12)
      months <- data.frame(NA)
      for (c in (1:12)) {
        f <- which(cycle(ts_pixel) == c)
        f <- f[!is.na(ts_pixel[f])]
        #numeric <- as.data.frame(sort(ts_corr[f]))
        #numeric <- sort(ts_pixel[f])
        numeric<-(ts_pixel[f])
        for(k in (1:length(numeric))){
          months[k,c]<-numeric[k]
        }
      }
      months2<-months
      #for(k in seq(1,6,1)){
      #months2[2:38,k]<-months2[1:37,k]
      #months2[1,k]<- NA
      #}
      for(j in seq(1,12,1)){
        m<-mean(months2[,j])
        s_dev<-sd(months2[,j])
        for(x in seq(1,35,1)){
          #months2[x,j] <- ((months2[x,j]-mean(na.omit(months2[,j])))/sd(na.omit(months2[,j])))
          if(!is.na(months2[x,j])){
            months2[x,j] <- ((months2[x,j]-m)/s_dev)
          }
          else{
            months2[x,j] <- NA
          }
        }
      }

      t_months <- t(months2)
      vec_tmonths <- as.vector(t_months)
      mat_tmonths <- as.matrix(vec_tmonths,nrow=456,ncol=1)
      #ini_try[,q] <- data.frame(mat_tmonths)
      t_D_df02[,i] <- data.frame(mat_tmonths)
    }
    #else{
```

```
      #ini_try[,q] <- pixel
      #t_D_df02[,i] <- pixel
    #}
  }

  return(t_D_df02)
}
#---------------------------------------------------------------- x -------------------------------------------
```

2. Soil Moisture

```
standardize<-function(DF_soilmoisture){
  D_df02_sm<-DF_soilmoisture
  t_D_df02<-as.data.frame(t(D_df02_sm))
  #ini_try<-data.frame(matrix(NA,nrow = 456,ncol = 2))
  #q=0
  for(i in seq(1,3366,1)){
    pixel<-t_D_df02[,i]
    pixel <- na.omit(pixel)
    #q=q+1
    if(length(pixel) != 0){
      #pixel <- data
      ts_pixel <- ts(pixel,start = c(1981,1),end = c(2018,12), frequency = 12)
      months <- data.frame(NA)
      for (c in (1:12)) {
        f <- which(cycle(ts_pixel) == c)
        f <- f[!is.na(ts_pixel[f])]
        #numeric <- as.data.frame(sort(ts_corr[f]))
        #numeric <- sort(ts_pixel[f])
        numeric<-(ts_pixel[f])
        for(k in (1:length(numeric))){
          months[k,c]<-numeric[k]
        }
      }
      months2<-months
      #for(k in seq(1,6,1)){
      #months2[2:38,k]<-months2[1:37,k]
      #months2[1,k]<- NA
      #}
      for(j in seq(1,12,1)){
        m<-mean(months2[,j])
        s_dev<-sd(months2[,j])
        for(x in seq(1,38,1)){
          #months2[x,j] <- ((months2[x,j]-mean(na.omit(months2[,j])))/sd(na.omit(months2[,j])))
          if(!is.na(months2[x,j])){
            months2[x,j] <- ((months2[x,j]-m)/s_dev)
          }
          else{
            months2[x,j] <- NA
          }
        }
      }

      t_months <- t(months2)
      vec_tmonths <- as.vector(t_months)
      mat_tmonths <- as.matrix(vec_tmonths,nrow=456,ncol=1)
      #ini_try[,q] <- data.frame(mat_tmonths)
```

```
      t_D_df02[,i] <- data.frame(mat_tmonths)
    }
    #else{
      #ini_try[,q] <- pixel
      #t_D_df02[,i] <- pixel
    #}
  }

  return(t_D_df02)
}
#------------------------------------------------------------------ x ------------------------------------------
```
